# Intermolecular Interactions of Large Systems: Boron Nitrides, Acenes, and Coronenes

**DOI:** 10.1021/acs.jpca.6c01896

**Published:** 2026-08-26

**Authors:** Vladimir Fishman, Jan M. L. Martin, A. Daniel Boese

**Affiliations:** † Department of Molecular Chemistry and Materials Science, 34976 Weizmann Institute of Science, Reḥovot 7610001, Israel; ‡ Department of Chemistry, 27267 University of Graz, Heinrichstrasse 28/IV, Graz 8010, Austria

## Abstract

In a recent contribution [Fishman et al., *J. Chem. Theory Comput.* **2025**, 21, 2311–2324], we introduced another approach to benchmarking noncovalent interactions by not benchmarking interaction energies of different dimers but by considering the evolution of interaction energies with increasing system size. Here, we extend the benchmark set to more species, such as electrostatically bound borazine dimers as well as the minimum-energy structures of parallel displaced acene and coronene dimers. While the minimum structures of the parallel displaced acene dimers yield similar results to previously published sandwich-structured acenes, the borazine dimers behave vastly differently, yielding a yet more complete picture of noncovalent interactions and their scalability. In contrast, the polycyclic aromatic hydrocarbon structures—coronenes sandwich-stacked and coronenes parallel displaced—give results consistent with those obtained for both types of the polyacene series, resulting in an updated estimate for the coronene dimer energy. By doing so, we are able to break down various approximations made by state-of-the-art coupled-cluster methods.

## Graphical abstract

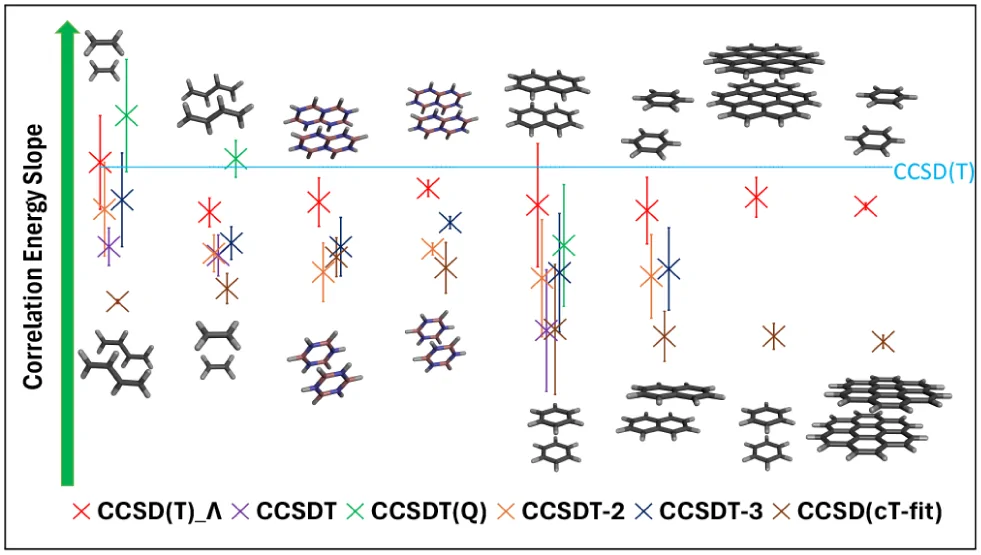

## Introduction

Noncovalent, also called intermolecular interactions, are at the forefront of modern science, as they determine the properties of many chemical systems, including molecular crystals,^1,2^ solvents,^3^ polymers,^4,5^ proteins, and nucleotides.^6^ These interactions also control the self-assembly of nanomaterials,^7^ drug docking processes,^8^ liquids, and the binding of reaction partners in supramolecular catalysis.^9^ Noncovalent interactions are weak contacts compared to covalent bonds and thus very sensitive to the environment. Therefore, their strength and nature should be predicted using robust, accurate quantum chemical methods with as few errors as possible.

In a paper from 2021,^10^ a large discrepancy of 25% was found between two reference methods solving the Schrödinger equation differently, namely fixed-node diffusion Monte Carlo (FN-DMC),^11^ which is strongly used in solid-state physics (but also substantially in molecular quantum chemistry^12^), and coupled-cluster (CC) theory including singles, doubles, and perturbative triples CCSD­(T)^13,14^ —which had been termed the “gold standard” in gas-phase quantum chemistry when utilized at its basis set limit.^15^ We have to note that, although CCSD­(T) was employed, it was done so within its local natural orbital (LNO) approximation, investigating a series of cutoff values. The systems that showed these deviations were, however, too large for other, more elaborate methods that might yield further insights than the methods used in ref 10.

We previously suggested another approach for benchmarking noncovalent interactions by considering the evolution of the correlation binding energy with respect to the number of subunits of dimer interactions.^16^ We do not look at mere interaction energies but at the deviations from the interaction energy slopes of increasing cluster sizes, which scale linearly to ever larger systems. Thus, we are able to extrapolate these deviations to huge systems, specifically at the sandwich-stacked polyacene series and at two types of geometries of alkapolyene dimers, assuming that the errors increase linearly as well.

For the sandwich-stacked polyacene series, which we show below to be closely related to the coronene dimer, where some of the discrepancies between CCSD­(T) and FN-DMC were found, we were able to perform extremely accurate rank-reduced^17^ CCSDT­(Q)^18^ calculations. CCSDT­(Q) is going beyond CCSD­(T), taking further excitation terms in the coupled cluster approximation such as full triples and perturbative quadruples into account. CCSDT­(Q) has a much steeper scaling with system size and is also very close to the exact solution of the electronic Schrödinger equation for closed-shell systems without much multireference character. By assuming that the CCSDT­(Q) estimate is close to the near-exact solution for intermolecular interactions,^19^ we arrived in ref 16 at a maximum deviation of 4.5% from CCSD­(T) and not 25% for the buckycatcher, for which the largest discrepancy to FN-DMC and LNO-CCSD­(T) had been reported in ref 10. Furthermore, for the coronene *parallel displaced* dimer, we arrived at a maximum deviation of 3.5% between CCSDT­(Q) and CCSD­(T) and not the 12% difference between LNO-CCSD­(T) and FN-DMC. Summarizing, whereas CCSD­(T) indeed tends to overestimate the dissociation energies of π-stacks, it does not do so with the large magnitude suggested by FN-DMC. Similar results have also been obtained for several polyaromatic hydrocarbon stacks, including benzene, using a simple underlying PPP Hamiltonian;^20^ here, however, the CCSDT­(Q) interaction energies exhibit larger dissociation energies than CCSD­(T), which is in contrast to the results when a HF wave function is utilized for CCSDT­(Q).

Despite these results, all polyacenes and polyaromatic hydrocarbon stacks were investigated in our last contribution^16^ at their sandwich, i.e., transition state structures, because we were able to utilize their high symmetry.^21^ One may argue that especially higher-order dispersion terms might be canceled at high symmetries,^22^ whereas at lower symmetries, they might not, which is also discussed in ref 23. For this purpose, we investigate **parallel-displaced** polyacene dimers that are global minimum structures for the polyacene series for naphthalene dimers and larger and nearly isoenergetic^24^ with the slanted T-shaped local minimum structure^25^ for the benzene dimer. Other types of geometries of polyacenes, specifically g-like, cross, and X, have been described in the most recent paper of Sharapa and coworkers.^26^ Additionally, we explore sandwich-structured and parallel-displaced coronene dimers. Furthermore, we considered two sandwich-like borazine dimer structures: one where the BN moiety is stacked toward NB, which exhibits a large electrostatic attraction, and the other one with the BN moiety stacked toward BN, yielding electrostatic repulsion.

This contribution is hence a direct continuation of our previous paper,^16^ introducing new species with (essentially perfect) lines to interpolate. By using DFT-based symmetry-adapted perturbation theory (DFT-SAPT), we distinguish these species and group them into different categories.

## Computational Details

Electronic structure calculations have been performed using various software packages, depending on the method employed. Geometries of the investigated systems in the paper have been optimized by density functional theory (DFT)^27,28^ utilizing the ω-B97M-V^29^ functional with the def2-QZVPP^30^ basis set (here, we omitted diffuse functions because of linear dependencies) using ORCA 5.0.3.^31^ Full molecular symmetry has imposed.

To achieve highly accurate single-point energy calculations for the optimized structures, various forms of coupled-cluster (CC) theory have been employed:

- The “gold-standard” CCSD­(T) (Coupled-Cluster with Single, Double, and Perturbative Triple Excitations),^13,14^ including Resolution of Identity (RI), also called Density Fitting (DF),^32,33^ for large systems, performed by the MRCC^34^ program package (August 2023 and later versions).
- LNO (local natural orbitals)-CCSD­(T)^35,36^ using the normal, tight, very tight (denoted vTight from hereon), and very very tight (vvTight) threshold settings with localcc = 2021.
- DLPNO (Domain-Based Local Pair Natural Orbital)-CCSD­(T_1_)^37–40^ using the TightPNO threshold setting with two different values for the Pair Natural Orbital (PNO) truncation (TCutPNO), in which orbitals with an occupation number less than TCutPNO are neglected. The TCutPNO parameters used are 10^–6^ and 10^–7^, respectively, and DLPNO-CCSD­(T_1_) calculations are done with ORCA 5.0.4.^31^
- PNO (Pair Natural Orbital Local)-CCSD­(T)^41^ with Domopt Tight, Domopt vTight, and Pairopt Tight truncation settings was performed by means of MOLPRO 2024.3.^42^
- The CCSD­(T)_λ_ method^43–46^ —which treats the triples as a perturbation to CCSD—was performed by the CFOUR program software^47^ (on a side note, the CCSD­(T) equations can be derived^48^ by approximating the Λ vector as the transpose of the doubles amplitudes vector *T*_2_).
- Post-CCSD­(T) methods, specifically CCSDT-2 and CCSDT-3,^49^ for the species with a maximum of two aromatic rings were performed using CFOUR.^47^ Both methods go beyond CCSD­(T) by introducing iterative *T*_3_, and the first formal increment beyond CCSD­(T) is the fifth-order TQ term. Then, the difference between CCSDT-2 and CCSDT-3 appears only at the sixth order of perturbation theory. It arises from the contribution of the effect on the *T*_3_ amplitudes equation of single excitations, which is neglected in CCSDT-2 but included in CCSDT-3 (see ref 50 for more details).

For more approximate single-point energy calculations, we have employed WFT (wave function theory)-based methods, including MP2 (second-order Møller–Plesset perturbation theory)^51^ using the MRCC^34^ software and MP3 (third-order Møller–Plesset perturbation theory) with its RI implementation^52^ in the Q-Chem 6.1.0 program package.^53^ Additionally, DFT-based methods are utilized, specifically the dRPA (direct random-phase approximation) as implemented in MRCC^34^ (August 2023) and DFT-SAPT (symmetry-adapted perturbation theory) as available in MOLPRO[Bibr ref42] 2022.2. These methods provide a lower-cost alternative for estimating correlation effects while maintaining a reasonable level of accuracy.

All single-point calculations have been performed using the correlation-consistent^54^ and augmented correlation-consistent^55^ polarized valence X-ζ (X = D, T, Q, 5) (aug-)­cc-pVXZ basis sets. These will be abbreviated henceforth with DZ, TZ, QZ, and 5Z, as well as aDZ, aTZ, aQZ, and a5Z, respectively.

## Results and Discussion

### Strong Correlation Diagnostics

To determine if our systems are indeed well-described by single determinant methods, we evaluate a strong correlation diagnostic^56^ for all series based on total atomization energy (TAE) calculations obtained with the TZ basis set using the TPSS^57^ functional:

1 $$\%{TAE}_{X}\left[\mathrm{KS}-\mathrm{HF}\left(\mathrm{TPSS}\right)\right]=100\%\times \frac{({\mathrm{TAE}}_{X}\left[\mathrm{TPSS}@\mathrm{TPSS}\right]-{\mathrm{TAE}}_{X}\left[\mathrm{TPSS}@\mathrm{HF}\right]}{TAE\left[\mathrm{CCSD}\left(T\right)\right]}$$

Thus, the correlation diagnostic is calculated by the difference in the total atomization energies between the TPSS exchange using a TPSS exchange wave function (*TAE*_*X*_[*TPSS*@*TPSS*]) and TPSS exchange using a Hartree–Fock exchange wave function (*TAE*_*X*_[*TPSS*@*HF*]) divided by the total atomization energy of CCSD­(T). The results obtained by eq 1 show small static correlation around 5% *TAE*_*X*_ for the polyacene PD series and negligible static correlation, less than 2%, for the borazine series, which, most importantly and interestingly, do not become much larger when the number of rings increases. Another (*TAE*_*X*_[(*T*)]) diagnostic from ref 56 (Table 1, Cluster 3) confirms these observations.

**Table 1:** Different DFT-SAPT Components (kJ/mol), Induction to Dispersion, and Electrostatic to Dispersion Ratios Using the cc-pV5z Basis Set

Dimer	*E*_total_	$E_{\mathrm{elec}}^{\left(1\right)}$	$E_{\mathrm{ex}}^{\left(1\right)}$	*E*_ind_	*E*_disp_	$\frac{E_{\mathrm{ind}}}{E_{\mathrm{disp}}}$	$\frac{E_{\mathrm{elec}}^{\left(1\right)}}{E_{\mathrm{disp}}}$
Benzene (sandwich)	–7.4	0.3	12.6	–0.8	–19.6	0.04	–0.02
Benzene (parallel displaced)	–11.8	–5.8	23.2	–2.6	–26.8	0.10	0.22
Ethylene	–0.4	1.1	1.0	–0.1	–2.4	0.04	–0.46
Borazine (syn)	–7.8	–2.0	15.2	–0.5	–20.5	0.02	0.10
Borazine (anti)	–13.0	–16.3	38.7	–2.2	–33.3	0.06	0.49
Naphthalene (sandwich)	–17.1	–3.0	28.1	–1.3	–40.8	0.03	0.07
Naphthalene (parallel displaced)	–26.0	–15.5	50.6	–5.2	–56.0	0.09	0.28
Coronene (sandwich)	–57.8	–20.1	87.9	–1.9	–123.2	0.02	0.16
Coronene (parallel displaced)	–78.1	–36.6	114.1	–3.0	–145.3	0.02	0.25
Tran*sec*-Butadiene (relaxed)	–3.4	1.3	6.3	–0.5	–10.5	0.05	–0.12
Tran*sec*-Butadiene (fixed)	–2.5	1.8	1.7	–0.2	–5.7	0.04	–0.31
BN Naphthalene (syn)	–14.4	–4.3	29.5	–0.8	–38.9	0.02	0.11
BN Naphthalene (anti)	–25.3	–36.4	80.7	–4.8	–65.0	0.07	0.56

Moreover, commonly used rule of thumb for the default *T*_1_ and *D*_1_ diagnostics, ≤0.02 ^58,59^ and ≤0.05,^60^ respectively, reaffirm that a single-determinant reference should provide adequate results for a single-reference coupled-cluster treatment for all investigated in our study systems.

Meanwhile, the HOMO–LUMO gap decreases progressively with the increase in the number of subunits.

For more details, see the SI on pages 81–87.

### Different Species Investigated

In order to distinguish the different species, we explore the type of their intermolecular interactions by the use of symmetry-adapted perturbation theory, in which the total energy is expanded as:

2 $$E_{total}=E_{elec}^{\left(1\right)}+E_{ex}^{\left(1\right)}+E_{ind}^{\left(2\right)}+E_{in\mathrm{d‐e}x}^{\left(2\right)}+E_{disp}^{\left(2\right)}+E_{dis\mathrm{p‐e}x}^{\left(2\right)}+E_{\Delta HF}$$

The induction $E_{\mathrm{ind}}=E_{\mathrm{ind}}^{\left(2\right)}+E_{\mathrm{ind‐ex}}^{\left(2\right)}+E_{\Delta \mathrm{HF}}$ and electrostatic $E_{\mathrm{elec}}^{\left(1\right)}$ energies have been obtained from DFT-SAPT, and their ratios to dispersion $E_{\mathrm{disp}}=E_{\mathrm{disp}}^{\left(2\right)}+E_{\mathrm{disp‐ex}}^{\left(2\right)}$ ,^61^ displayed in Table 1, have been found to be most instructive when trying to separate dispersion-dominated from induction-dominated systems.

A more detailed inspection would analyze the percentage of the mean contribution of a fitted leading-order dispersion *C*_6_ coefficient in the SAPT dispersion contribution, which is very small for induction-dominated systems.^62^

Based on the ratios reported in Table 1, four distinct types of systems can be distinguished. As noted in ref 16, the π-stacks and alkapolyene stacks behave quite differently when compared to each other. This can be seen especially in the electrostatic to dispersion ratio, which is negative for the ethylene and trans-butadiene dimers, while it is positive for benzene and naphthalene (with the exception of sandwiched benzene, which is very slightly negative). Since benzene and naphthalene have sizable quadrupole moments, this is visible in the electrostatic interaction between these species. The borazine and BN-naphthalene dimers are distinctly different again, as a partial charge can be found on the nitrogen (negative) and boron (positive) atoms. This is especially visible in the anti configuration, where the largest electrostatic contribution of all dimers has been found. The syn configurations exhibit a more repulsive interaction, which is, however, in its electrostatic component, countered by the quadrupole and higher moments, making this configuration different again. We can already hypothesize that the alkapolyene stacks, π-stacks, borazine stacks (syn), and borazine stacks (anti) all behave quite differently, giving some insight about the nature of their intermolecular interactions.

### Basis Set Effects

In order to evaluate different basis sets, we compare results obtained at the LNO-CCSD­(T) level of theory with a Tight threshold. These are probably best suited, as calculations for 6-ring systems are feasible for this method even in the a5Z basis set. The correlation part of π-stacked molecules scales linearly with system size, yielding straight lines with *R* ^2^ > 0.99 (Figure 1).

**Figure 1:**
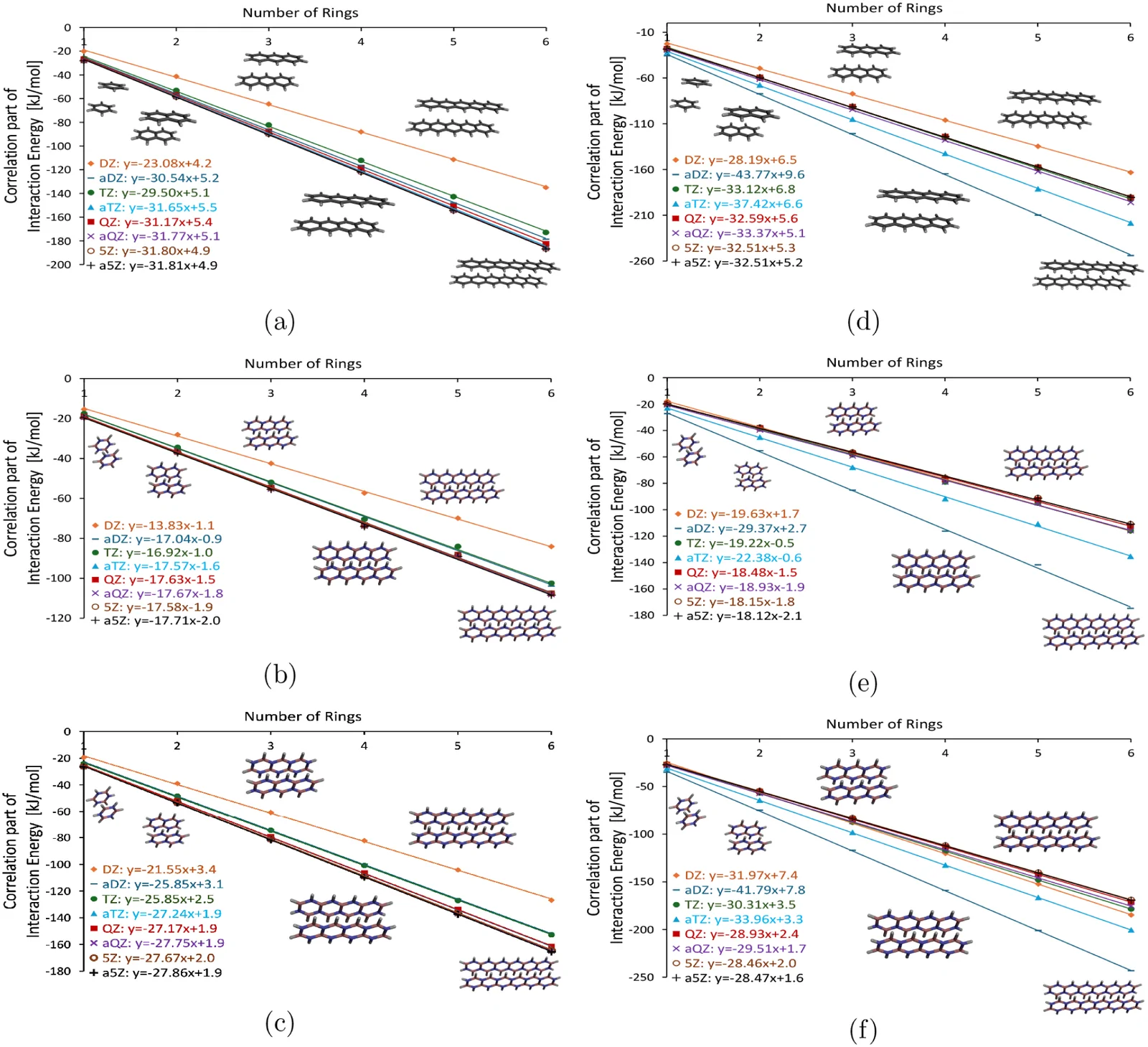
Correlation energies in kJ/mol vs number of rings using the cc-pV*n*Z and aug-cc-pV*n*Z (*n* = D-5) basis sets at the LNO-CCSD­(T) level with Tight threshold, including MP2 corrections: (a) cp-corr energies for acenes PD; (b) cp-corr energies for BN*_x_* ···NB*_x_*; (c) cp-corr energies for BN*_x_* ···BN*_x_*; (d) cp-uncorr energies for acenes PD; (e) cp-uncorr energies for BN*_x_* ···NB*_x_*; (f) cp-uncorr energies for BN*_x_* ···BN*_x_*.

Counter-poise (cp) corrected and counter-poise uncorrected slopes of each series have different behavior of convergence to the slope that is obtained from complete basis set limit noncovalent interaction energies. For instance, despite that both cp-corr and cp-uncorr {Q,5}­Z slopes of parallel displaced polyacene series give the same result, cp-uncorr *n*Z slopes do not converge smoothly to this result, whereas cp-corr *n*Z slopes do. In contrast, in the BN_*x*_···NB_*x*_ series, we can observe an opposite situation: cp-uncorr slopes exhibitconvergence to {Q,5}­Z, while cp-corr slopes do not. For BN_*x*_···BN_*x*_, we makea highly unusual observation: cp-corr *n*Z and a*n*Z slopes converge toward cp-uncorr {Q,5}­Z ; cp-uncorr *n*Z and a*n*Z slopes converge toward cp-corr {Q,5}­Z.

Intercepts are much more sensitive to the basis set in all types of investigated systems, especially cp-uncorrected intercepts. Interestingly, the aDZ cp-uncorrected intercepts are even worse than the cp-uncorrected DZ ones (see Figure 1d–f). Thus, for the aDZ basis set, there seems to be some imbalance introduced by the diffuse functions.

Based on Figure 1a–c and corresponding cp-corrected slopes, we can summarize that the QZ basis set appears as the best compromise to get accurate slope values for a larger series of parallel displaced polyacenes and BN_*x*_···BN_*x*_. Practically, from a computational cost point of view, even the TZ basis set slopes are satisfactory enough, because the deviation cp-corrected TZ slope from the cp-corrected {Q,5}­Z slope is only 5–8% (namely, 5% for polyacene PD dimers and for BN_*x*_···NB_*x*_, and 8% for BN_*x*_···BN_*x*_). To reach the sub-kJ/mol accuracy, further corrections are required. Optimally, if the computing resource allows, it is better to use QZ because its slope deviation to CBS is less than 4% for all systems.

### Local Correlation

Having established the basis set sensitivity, we turn to estimating the canonical CCSD­(T) results at the complete basis set limit. With the advent of local correlation methods in coupled-cluster theory and extrapolating their cutoff parameters, calculations of large systems become feasible, and we can compare local methods to canonical CCSD­(T) results. For this purpose, we approximate canonical CCSD­(T) by several local coupled-cluster methods with the small DZ basis sets (both cp-corrected and cp-uncorrected DZ; see Figure 2), where we can compute canonical CCSD­(T) for all systems. Again, perfectly linear relationships are observed for all variants of the CCSD­(T).

**Figure 2:**
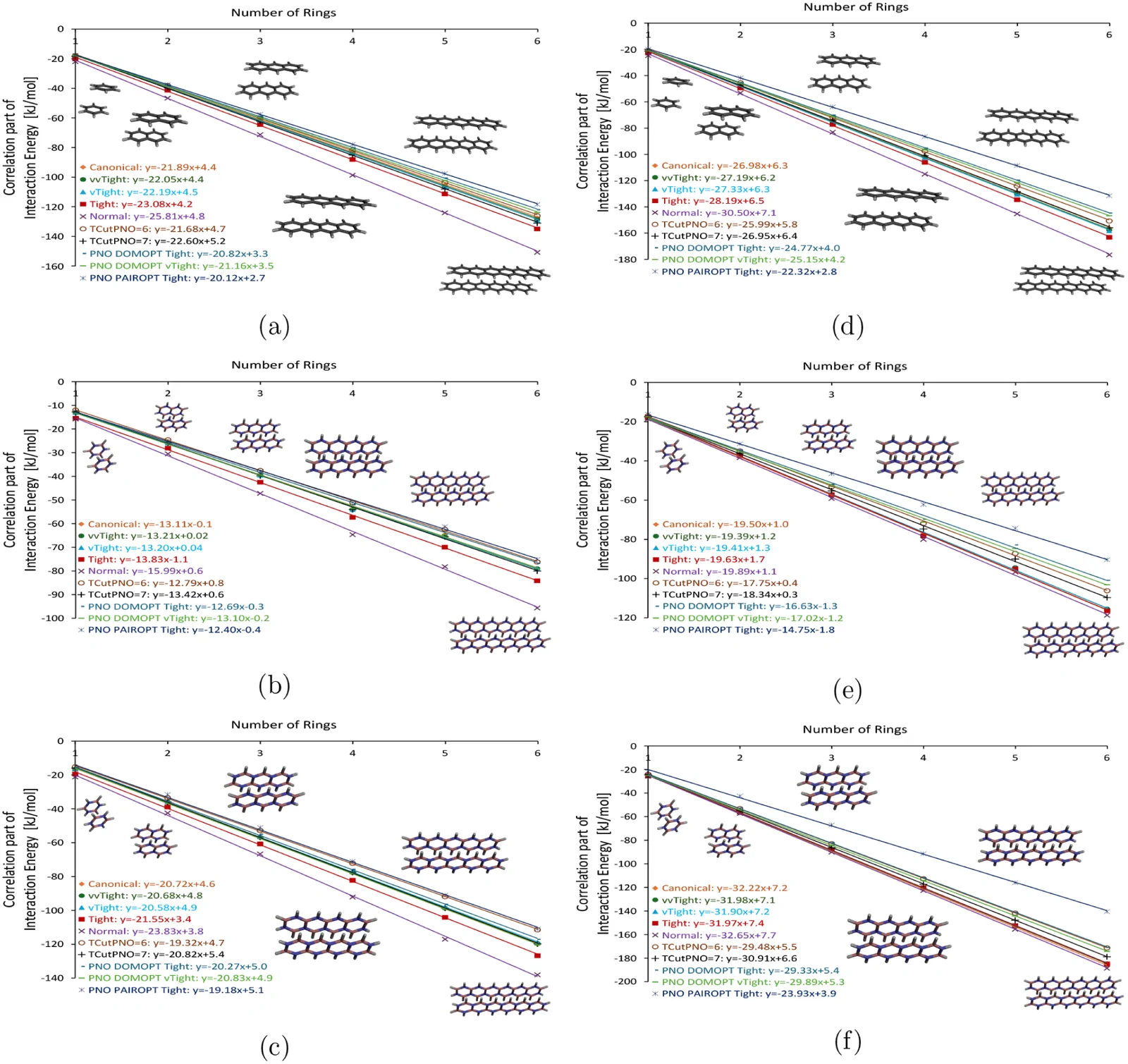
Correlation part of interaction energies (kJ/mol) derived from various coupled cluster methods as a function of system size per number of rings: (a) cp-corr DZ energies for acenes PD; (b) cp-corr DZ energies for BN*_x_* ···NB*_x_*; (c) cp-corr DZ energies for BN*_x_* ···BN*_x_*; (d) cp-uncorr DZ energies for acenes PD; (e) cp-uncorr DZ energies for BN*_x_* ···NB*_x_*; (f) cp-uncorr DZ energies for BN*_x_* ···BN*_x_*.

The first local variant investigated is local natural orbital (LNO) coupled-cluster, where the LNO cutoff threshold setting increases as Normal → Tight → vTight → vvTight. Here, the slope converges toward canonical CCSD­(T) most of the time. When a cp-corrected DZ basis set is applied, the successive differences in slopes for polyacene PD systems when going from Normal to Tight to vTight to vvTight LNO thresholds to canonical CCSD­(T) are 2.73, 0.89, 0.14, and 0.16 kJ/mol per subunit, respectively, showing a systematic convergence. For the BN_*x*_···BN_*x*_ series, no systematic convergence in the slope differences is observed, as the slopes change by 2.28, 0.97, −0.10, and −0.04 kJ/mol per subunit. Despite the fact that, as expected, LNO-CCSD­(T)-vvTight gives the closest result to canonical-CCSD­(T), the slope differences for different CCSD­(T) methods somewhat unexpectedly increase along Normal → Tight → canonical → vvTight → vTight. For the BN_*x*_···NB_*x*_ series, the differences in slopes are 2.16, 0.63, −0.01, and 0.10 kJ/mol per subunit, implying that the LNO-CCSD­(T)-vTight slope value is closer to canonical-CCSD­(T) than LNO-CCSD­(T)-vvTight.

It should be pointed out that in our previous paper,^16^ the last vTight point at the cp-corrected aTZ value was unfortunately incorrect, resulting in an erroneous regression line in Figure 2c for LNO-CCSD­(T)-vTight. The corresponding LNO-CCSD­(T)-vTight line, hence, changes from *y* = −22.05 × *x* – 1.60 kJ/mol to *y* = −23.78 × *x* + 2.4 kJ/mol. The corrected Figure 2c for ref 16 is provided in the Supporting Information of the present paper.

Based on the foregoing analysis, LNO-CCSD­(T) **underestimates** canonical-CCSD­(T) in the calculation of noncovalent interaction energy for the polyacene PD and BN_*x*_···NBx series (as well as for sandwich-structured polyacenes, as described in ref 16). For the more electrostatically bound BN_*x*_···BN_*x*_ series, LNO **overestimates** canonical-CCSD­(T).

Using the same DZ cp-corrected basis set for the second tested local method, Pair Natural Orbital Local CCSD­(T) [PNO-LCCSD­(T)], when going from the Pairopt-Tight threshold to Domopt-Tight to Domopt-vTight to canonical CCSD­(T), the slopes change by −0.70, −0.34, and −0.73 kJ/mol per subunit for the polyacene PD series, respectively. Thus, PNO-LCCSD­(T) is showing a systematic convergence, but with a negative sign, unlike the LNO approximation. For the PNO approximation, a similar trend of convergence is observed for the BN_*x*_···NB_*x*_ series, where the slopes change by −0.29, −0.41, and −0.01 kJ/mol per subunit. In contrast, for the BN_*x*_···BN_*x*_ series, a nonsystematic convergence of slopes is observed, changing by −1.09, −0.56, and +0.11 kJ/mol per subunit. For all types of local methods applied, the PNO-LCCSD­(T) approximation gives the closest slope to canonical CCSD­(T) with the Domopt-vTight parameter.

As a third method, Domain-Based Local Pair Natural Orbital Coupled-Cluster [DLPNO-CCSD­(T_1_)] has been utilized. Here—even when employing counterpoise corrections—we cannot make a definitive conclusion regarding the optimal TCutPNO parameter for the PNO space of the DLPNO approximation. For the polyacene PD series, the TCutPNO parameter of 10^–6^ yields a slope closer in value to canonical CCSD­(T) than a parameter of 10^–7^. For the BN_*x*_···BN_*x*_ series, 10^–7^ yields a closer result, while for the BN_*x*_···NB_*x*_ series both cutoff parameters are equally far from the canonical CCSD­(T) slope.

As discussed in the previous section, the deviation of counterpoise (cp)-corrected TZ slopes is not as far from CBS as DZ ones. Moreover, the Natural Orbital Approximations compress DZ less than the larger basis sets, and thus the DZ basis might not be fully representative. Hence, the analysis of results obtained with the TZ basis is giving a more complete picture, although we have fewer species to compare to. As far as canonical-CCSD­(T)/TZ calculations are feasible for at least four systems in each series, we can consider the convergence for different local coupled-cluster theories to canonical-CCSD­(T) for the 1→4 TZ slope. See relevant Figure S1 on page 36 in SI. The convergence of the slope in the PNO-LCCSD­(T) approximation has the trend Pairopt-Tight → Domopt-Tight → Domopt-VTight → canonical for all systems.

For LNO-CCSD­(T) in combination with the cp-corrected TZ basis set, the thresholds converge as expected with increasing cutoffs: Normal → Tight → vTight → vvTight → canonical. The same is happening for the cp-uncorrected TZ basis set with the exception of the BN_*x*_···BN_*x*_ series, in which, interestingly, the Tight threshold reproduces the canonical result with the lowest deviation.

For the DLPNO approximation, the BN_*x*_···NB_*x*_ series is not as convergent for the different cutoffs in the case of the cp-corrected TZ basis set: the TCutPNO parameter of 10^–6^ overestimates canonical results, while the TCutPNO parameter of 10^–7^ underestimates it, but for larger systems (with 3 or 4 rings), TCutPNO = 10^–7^ results are reliable. This behavior of DLPNO convergence may be affected not only by the TCutPNO parameter but also by TCutDO, which dominantly controls the length of the interaction and can lead to different convergence directions: tightening TCutDO converges the DLPNO energy toward the canonical limit from below, whereas tightening other thresholds can converge from above.^63,64^ In all other cases, each series converges from 10^–6^ → 10^–7^ → canonical.

Related to this, LNO-CCSD­(T) (vTight and vvTight threshold settings) and PNO-LCCSD­(T) (DOMOPT = vTight setting) are both equally reliable and have provided similar deviations from canonical CCSD­(T). However, in our further analysis, we focus on the LNO approximation as far as it has more predictable convergence threshold-to-threshold error behavior.^65^

### Electronic Structure Methods

Having estimated the slopes of the canonical basis set limit CCSD­(T) and thus its performance for large molecules, we evaluate the efficiency of more approximate electronic structure methods. Here (Figure 3), we investigate several post-Hartree–Fock methods, such as MP2, MP2.5, and MP3; the dispersion contribution of DFT-SAPT, as well as dRPA. To determine the degree of agreement of the approximate methods with the “gold standard” canonical CCSD­(T), we compare absolute values of slopes obtained using these approximate methods, ultimately determining their quality within this metric. These approximate methods can help us to understand which physical aspects—second-order dispersion, third-order perturbative corrections, connected doubles, perturbative triples, dynamical screening, electrostatics, induction, and dispersion—are actually responsible for the interaction trend.

**Figure 3:**
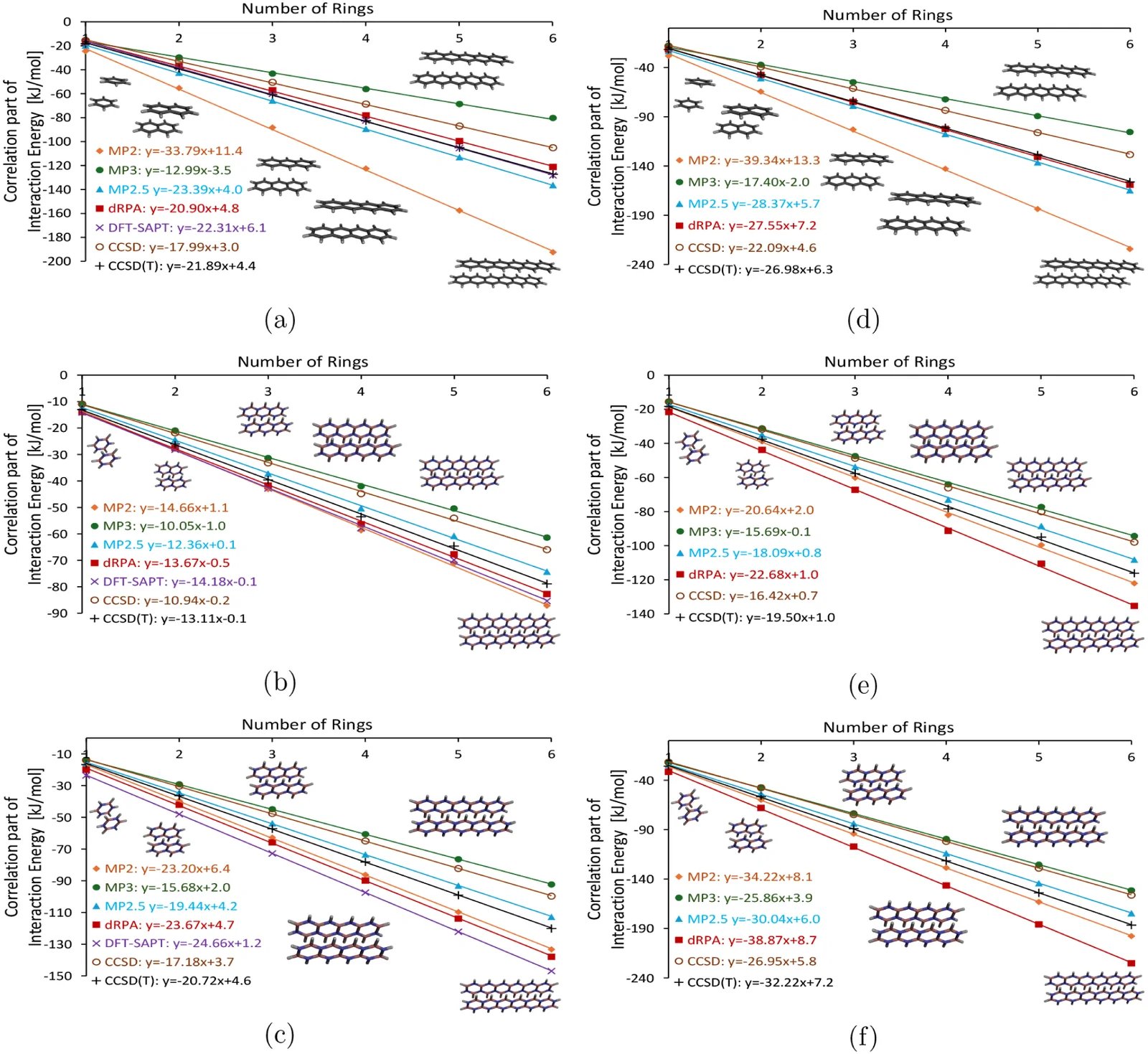
Correlation part of interaction energies derived from various post-Hartree–Fock (and DFT) methods in kJ/mol vs number of rings: (a) cp-corr DZ energies for acenes PD; (b) cp-corr DZ energies for BN*_x_* ···NB*_x_*; (c) cp-corr DZ energies for BN*_x_* ···BN*_x_*; (d) cp-uncorr DZ energies for acenes PD; (e) cp-uncorr DZ energies for BN*_x_* ···NB*_x_*; (f) cp-uncorr DZ energies for BN*_x_* ···BN*_x_*.

Slope values for the parallel displaced polyacene systems exhibit the trend MP3 < CCSD < dRPA ≈ CCSD­(T) ≈ DFT-SAPT < MP2.5 < MP2 for cp-corrected and cp-uncorrected (excluding DFT-SAPT for the latter, as DFT-SAPT is only defined when using cp-corrections). As was described earlier for sandwich polyacene dimers (see section “Electronic Structure Methods” in ref 16), for the parallel displaced polyacene dimers, MP2 also significantly overestimates interaction energies, whereas MP3 significantly underestimates them, and, which is as well important to note, DFT-SAPT and dRPA are closer to CCSD­(T) than the post-Hartree–Fock methods investigated. The polyacene series has a strongly size-dependent post-MP2 regime in which finite-order Møller–Plesset perturbation theory is unreliable.

An identical trend for cp-corrected slope values is observed for the BN_*x*_···NB_*x*_ dimers. Here, deviations of MP2 and MP3 slopes are not as large from CCSD­(T) (12% and 23%, respectively), while for polyacene dimers the same deviations are as big as 50%. BN_*x*_···NB_*x*_ still has non-negligible post-MP2 correlation, but the positive CCSD/MP3-type correction is partially canceled by the negative triples contribution. The relatively small final MP2 error is thus probably due to error cancellation effects. For BN_*x*_···NB_*x*_, dRPA demonstrates the best approximation to CCSD­(T). dRPA reproducing CCSD­(T) indicates that screened correlation/ring correlation is sufficient to describe the total size-dependent trend. In the case of cp-uncorrected slope values for the BN_*x*_···NB_*x*_ dimers, the MP2 and MP2.5 methods are the best approximation to CCSD­(T), while MP3 still underestimates interaction energies.

In contrast to the polyacene PD series, the slopes of the BN_*x*_···BN_*x*_ systems exhibit a very different behavior: MP3 < CCSD < MP2.5 < CCSD­(T) < MP2 < dRPA < DFT-SAPT. Here, DFT-SAPT and dRPA are, interestingly, inferior to the MP2.5 and MP2 methods, whereas MP3 still noticeably underestimates interaction energies. The apparent success of MP2 or MP2.5 in the BN_*x*_···BN_*x*_ series is most likely due to favorable error cancellation as far as the MP2–MP3 difference is large and systematic, not because the underlying physics is purely second-order.

The larger post-MP2 sensitivity observed for polyacenes is consistent with its substantially smaller HOMO–LUMO gap (see SI on pages 86–87) that indicates lower-energy electronic excitations and enhanced polarizability. Since the MP2 correlation energy depends inversely on occupied–virtual energy denominators, smaller gaps can amplify second-order amplitudes and make higher-order screening and triples effects essential.

### Best Estimates of Slopes

Unfortunately, many calculations using the “gold-standard” canonical CCSD­(T) method are doable only with the DZ basis set. Thus, we can either calculate each system that belongs to the investigated sequence with this (or another) rather small basis set or increase the basis set size and calculate a lesser number of systems, thus adding an additional dimension to the problem. Unfortunately, CCSDT and CCSDT­(Q) calculations for the parallel-displaced naphthalene dimer or diborazine-fused sandwich dimers are currently computationally not feasible even with a “truncated” DZ basis set with no *p*-functions on hydrogen. Thus, we consider the post-CCSD­(T) methods CCSDT-2 and CCSDT-3 ^49^ instead of CCSDT, as they scale as *O*(*N* ^7^) rather than *O*(*N* ^8^) with the number of electrons. Deviations of CCSDT­(Q) from CCSD­(T) can be estimated using CCSD­(T)_λ_, as the correlation part of CCSD­(T)_λ_ seems to be somewhat closer to the correlation part of CCSDT­(Q) than CCSD­(T) (see Figure 4, SI of ref 16 and ref 22).

**Figure 4:**
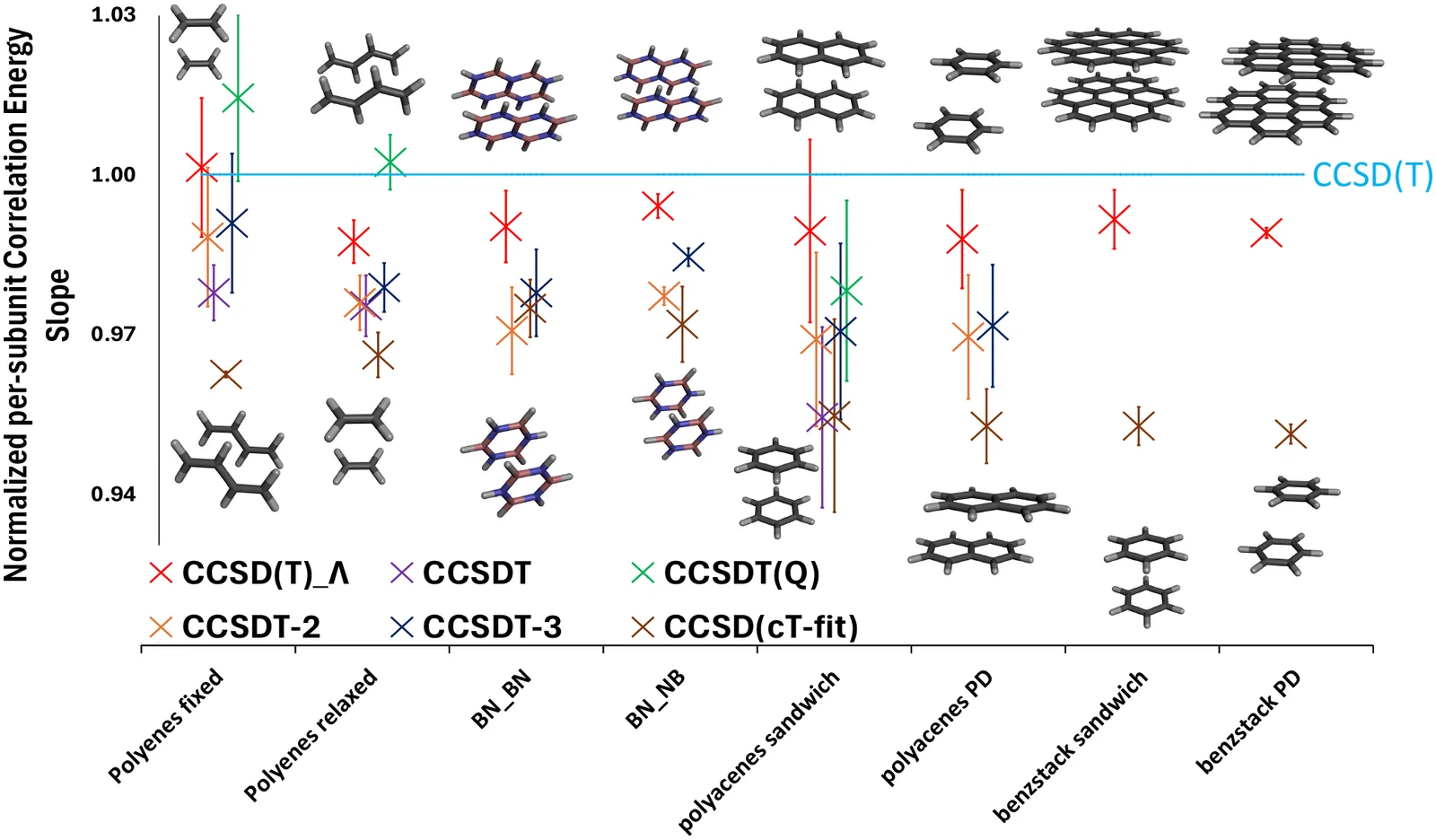
Relative deviations of correlation energies from the reference CCSD­(T) method. The first two dimers (e.g., benzene and naphthalene dimer) of each series are displayed in the relevant columns.

An anomaly is seen for the MP2 correlation energy of the coronene dimer when diffuse functions are added to the basis set. Ordinarily, it is well-known that adding diffuse functions accelerates basis set convergence in covalent bond energies and noncovalent interactions alike.^66^ However, for this extended planar system, we observe the opposite: in particular, the “spread” between cp-corrected and cp-uncorrected calculations is much larger for aug-cc-pV­{*n* – 1,*n*}­Z extrapolations than for their cc-pV­{*n* – 1,*n*}­Z counterparts. The root cause is numerical rather than quantum mechanical: diffuse functions cause serious near-linear dependence in the basis set, as reflected in the smallest eigenvalues of the overlap matrix *S* dipping into the 10^–11^–10^–12^ region for aug-cc-pV*n*Z (vs 10^–6^–10^–8^ for cc-pV*n*Z). As a result, the SCF in the former case becomes numerically ill-conditioned: the remedy of codes like MRCC is to discard all eigenvectors of *S* below a certain cutoff (by default 10^–7^). Alas, in this case there are many more eigenvectors to be discarded in the dimer than the two sets of monomer “discards”, which causes a size-consistency issue in the cp-uncorrected calculation. (The cp-corrected is not affected in the same manner, as *S* is the same for dimer and monomer+ghost).

Thus, in this study, we use the cc-pV­{Q,5}­Z basis set extrapolation instead of the aug-cc-pV­{Q,5}­Z one. The slopes of the correlation energy, extrapolated to cp-uncorrected and cp-corrected complete basis set limits, for all investigated series are summarized in Table 2, while Table 3 summarizes the slopes of the full interaction energies obtained by different WFT-based methods.

**Table 2:** Best Available cc-pV­{Q,5}­Z Correlation Energy Slopes (kJ/mol Per Subunit)^a^

Method										
CCSD(T)							post-CCSD(T)			
System	MP2	CCSD	Tight	vTight	canonical	CCSD(T)_λ_ ^b^	CCSDT-2^b^ ^b^	CCSDT-3^b^ ^b^	CCSDT^b^,^c^	CCSDT(Q)^b^,^c^
BN_*x*_···NB_*x*_	–18.90	–13.64	–17.53	–17.90	–17.66	–17.58	–17.29	–17.42		
BN_*x*_···BN_*x*_	–30.83	–22.86	–28.20	–27.98	–28.26	–28.02	–27.51	–27.71		
Acene PD	–45.40	–24.86	–32.45	–31.75	–31.51	–31.21	–30.71	–30.77		
Acenes^d^	–34.22	–18.41	–24.89	–24.43	–23.13	–22.90	–22.44	–22.47	–22.08	–22.64
Polyene Relax.	–11.02	–7.06			–8.75	–8.64	–8.55	–8.57	–8.55	–8.78
Polyene Fixed	–4.87	–3.06			–3.82	–3.78	–3.73	–3.74	–3.72	–3.82
Coronene PD	–31.96	–16.59	–22.27	–21.48	–20.97	–20.78			–20.25	–20.76
Coronene Sandwich	–28.52	–15.10	–20.46	–19.72	–19.04	–18.90				
BN_*x*_···NB_*x*_	–19.35	–14.42	–17.81	–17.49	–17.79	–17.66	–17.35	–17.48		
BN_*x*_···BN_*x*_	–30.64	–22.63	–27.98	–27.67	–27.95	–27.64	–27.05	–27.25		
Acene PD	–45.34	–24.49	–32.44	–31.57	–31.09	–30.63	–29.98	–30.05		
Acenes^d^	–34.96	–19.16	–24.86	–24.44	–23.98	–23.71	–23.21	–23.25	–22.88	–23.44
Polyene Relax.	–11.04	–7.00			–8.68	–8.57	–8.46	–8.49	–8.45	–8.69
Polyene Fixed	–4.86	–3.01			–3.83	–3.88	–3.83	–3.84	–3.76	–3.94
Coronene PD	–32.15	–16.76	–22.29	–21.59	–21.01	–20.74			–19.95	–20.58
Coronene Sandwich	–28.66	–15.10	–20.43	–19.68	–18.87	–18.69				

a All numbers are estimates, except LNO-CCSD­(T)-Tight. The top part of the table shows the cp-corrected results, and the cp-uncorrected ones are in the bottom part. Detailed tables are given in the SI on pages 61–66.

b The post-CCSD­(T) and CCSD­(T)_λ_ slopes have been scaled by a factor of 1.4 to account for the small cc-pVDZ basis set size—see discussion in ref 16 and in its Supporting Information on pages 83–89.

c CCSDT and CCSDT­(Q) slopes for coronene PD have been estimated according to the respective differences with CCSD­(T) for the hexacene sandwich-structured dimer; see discussion below.

d Re-estimated values for sandwich-structured acene series with the most recent MRCC version.

**Table 3:** Best Available cc-pV­{Q,5}­Z Energy Slopes, This Time Including Hartree–Fock (kJ/mol Per Subunit), Similar to Table 2 ^a^

Method										
CCSD(T)							post-CCSD(T)			
System	MP2	CCSD	Tight	VeryTight	canonical	CCSD(T)_λ_ ^b^	CCSDT-2^b^	CCSDT-3^b^	CCSDT^b^ ^,^ ^c^	CCSDT(Q)^b^ ^,^ ^c^
BN_*x*_···NB_*x*_	–8.36	–3.10	–6.99	–7.36	–7.11	–7.03	–6.75	–6.88		
BN_*x*_···BN_*x*_	–19.84	–11.87	–17.20	–16.99	–17.26	–17.03	–16.51	–16.72		
Acene PD	–30.97	–10.44	–18.02	–17.33	–17.08	–16.78	–16.28	–16.34		
Acenes^d^	–22.27	–6.47	–12.95	–12.48	–11.18	–10.96	–10.49	–10.53	–10.14	–10.70
Polyene Relax.	–5.72	–1.76			–3.45	–3.35	–3.25	–3.28	–3.25	–3.48
Polyene Fixed	–3.38	–1.56			–2.33	–2.28	–2.23	–2.25	–2.23	–2.33
Coronene PD	–23.54	–8.17	–13.85	–13.06	–12.55	–12.36			–11.83	–12.34
Coronene Sandwich	–18.87	–5.45	–10.81	–10.07	–9.39	–9.25				
BN_*x*_···NB_*x*_	–8.49	–3.56	–6.95	–6.63	–6.93	–6.80	–6.49	–6.62		
BN_*x*_···BN_*x*_	–19.06	–11.04	–16.40	–16.08	–16.37	–16.06	–15.47	–15.67		
Acene PD	–30.64	–9.79	–17.74	–16.86	–16.39	–15.93	–15.27	–15.35		
Acenes^d^	–23.02	–7.22	–12.92	–12.50	–12.04	–11.77	–11.27	–11.32	–10.94	–11.50
Polyene Relax.	–5.63	–1.59			–3.27	–3.16	–3.05	–3.08	–3.04	–3.28
Polyene Fixed	–3.29	–1.45			–2.26	–2.32	–2.27	–2.28	–2.20	–2.38
Coronene PD	–23.55	–8.16	–13.69	–12.99	–12.41	–12.14			–11.35	–11.98
Coronene Sandwich	–18.86	–5.30	–10.63	–9.87	–9.07	–8.88				

a All numbers are estimates, except LNO-CCSD­(T)-Tight. The top part of the table shows the cp-corrected results, and the cp-uncorrected ones are in the bottom part.

b The post-CCSD­(T) and CCSD­(T)_λ_ slopes have been scaled by a factor of 1.4 to account for the small cc-pVDZ basis set size—see discussion in ref 16 and in its Supporting Information on pages 83–89.

c CCSDT and CCSDT­(Q) slopes for coronene PD have been estimated according to the respective differences with CCSD­(T) for the hexacene sandwich-structured dimer.

d Re-estimated values for sandwich-structured acene series with the most recent MRCC version.

The CCSD­(T) CBS lines for all our systems up to the hexacene analogues (indicated by 1 → 6) are obtained as follows:

y=(−7.0±0.1kJ/mol)×x−0.4±0.4kJ/mol

per ring including HF for the BN_*x*_···NB_*x*_ series,

y=(−16.8±0.4kJ/mol)×x+3.4±0.4kJ/mol

per ring including HF for the BN_*x*_···BN_*x*_ series, and

y=(−16.7±0.3kJ/mol)×x+6.2±0.1kJ/mol

per ring including HF for the parallel displaced polyacene series.

The best estimate CCSD­(T)_λ_/cc-pV­{Q,5}­Z interaction energies for the benzene and naphthalene parallel displaced dimers are −11.4 ± 0.3 and −26.3 ± 0.8 kJ/mol, respectively, resulting in a slope value of −14.9 kJ/mol per subunit, similar to the one discussed above.

It is possible to arrange the systems according to the strength of correlation: polyacenes pd > BN···BN > polyacenes sandwich > coronene pd > coronene sandwich > BN···NB > polyene relaxed > polyene fixed. The parallel displaced acene series has the strongest correlation, while polyenes are weakly bound. Thus, slope value (kJ/mol per subunit) may be considered as a correlation indicator in any correlated-consistent basis set.

### Coronene Dimer

Of special interest is the radially fused polycyclic aromatic hydrocarbon series going from Benzene → Coronene → Circumcoronene, as the coronene dimer was heavily investigated in the past.

Following the same recipe of slope and intercept estimation as above (see also SI on pages 76–78), we are able to estimate the interaction energy of the parallel displaced coronene dimer. Table 4 and Figure 5 show the interaction energy of the parallel displaced coronene dimer at different levels of computational theory with various estimation techniques. Here, our CCSD­(T)/CBS result is close to the results reported by previous groups, with LNO-CCSD­(T) overestimating the canonical interaction energy for hydrocarbons.

**Table 4:** Interaction Energies for the Coronene Parallel Displaced Dimer at Different Levels of Theory in the Complete Basis Set Limit^a^ ^c^

Level of theory	Interaction energy	Reference	Basis
MP2	–161.1 ± 2.1	Table 1 in ref 67	Plane Wave
MP2	–159.3	Table S1 in ref 68	CBS
MP2	–161.2 ± 0.2	this work	CBS {Q,5} extrap.
MP2	–156.7 ± 2.5	this work	CBS a{Q,5} extrap.
CCSD	–56.1 ± 2.1	Table 1 in ref 67	Plane Wave
CCSD	–55.4 ± 0.4	this work	CBS {Q,5} extrap.
DLPNO-CCSD(T_0_)	–87.6 ± 1.8	Table 1 in ref 68	CBS
PNO-LCCSD(T)-F12 (domopt = tight)	–83.6 (cp-corr)	Table 10 in ref 41	C:aug-cc-pVTZ; H:cc-pVTZ
PNO-LCCSD(T)-F12 (domopt = tight+)	–80.8 (cp-corr)	Table 6 in ref 69	C:aug-cc-pVTZ; H:cc-pVTZ
PNO-LCCSD(T*)-F12 (domopt = tight+)	–83.7 (cp-corr)	Table 6 in ref 69	C:aug-cc-pVTZ; H:cc-pVTZ
LNO-CCSD(T) (Tight/vTight extrap.)	–86.2 ± 2.5	Table 1 in ref 10	cc-pV{Q,5}Z
LNO-CCSD(T)-vTight	–90.0 ± 0.6	this work	CBS {Q,5} extrap.
LNO-CCSD(T) (Tight/vTight extrap.)	–87.5 ± 0.4	this work	CBS {Q,5} extrap.
CCSD(T)	–86.4	Tables 4, 5 in ref 70	CBS
CCSD(T)	–88.3 ± 2.1	Table 1 in ref 67	Plane Wave
CCSD(T)	–86.6 ± 0.8	this work	CBS {Q,5} extrap.
CCSD(T)_λ_	–84.9 ± 1.1	this work	CBS {Q,5} extrap.
CCSDT(Q)^b^	–83.1 ± 1.3	this work	CBS {Q,5} extrap.
FN-DMC	–75.7 ± 3.3	Table 1 in ref 10	
FN-DMC	–73.4 ± 0.1	Table 1 in ref 71	def2-QZVP
CCSD(cT)	–80.8 ± 2.1	Table 1 in ref 67	Plane Wave
CCSDT^b^	–79.1 ± 1.7	this work	CBS {Q,5} extrap.

a All values are reported in kJ/mol.

b CCSDT and CCSDT­(Q) results have been estimated according to the respective difference with CCSD­(T) for the hexacene sandwich-structured dimer.

c Values for this work can also be found in Table S222 on page 77 of the SI.

**Figure 5:**
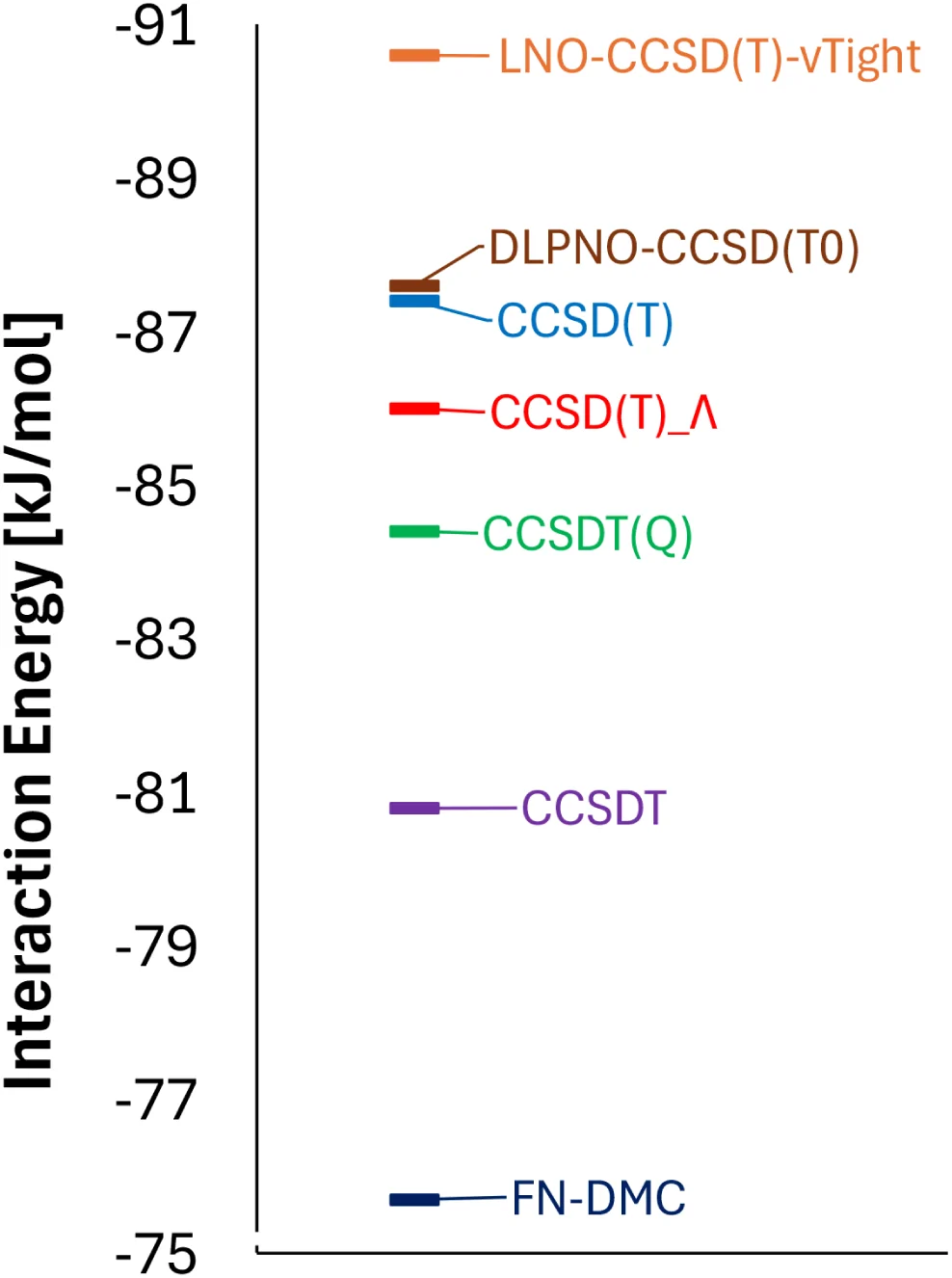
Interaction energies in kJ/mol for the coronene parallel displaced dimer at the different levels of theory. The CCSDT and CCSDT­(Q) values are estimates from the similarly behaving acene sandwich interaction energies.

During the reevaluation of the interaction energies for the sandwich-structured polyacene series, we noticed that the [(*T*)_λ_ – (*T*)]/CBS­(cp-corr) difference for the sandwich-structured hexacene dimer, which is 1.34 kJ/mol, is surprisingly close to the [(*T*)_λ_ – (*T*)]/CBS­(cp-corr) difference for the parallel displaced coronene dimer of 1.37 kJ/mol. Assuming a similar behavior of other post-CCSD­(T) corrections for π-conjugated aromatic systems, we estimate the (*Q*) – (*T*) difference for coronene PD to be expressed mathematically through a (*Q*) – (*T*) difference for sandwich-structured hexacene, such as:

3 $${[(Q)-(T)]}_{\mathrm{coronene}\;\mathrm{PD}}\approx {[{\left(T\right)}_{\lambda }-\left(T\right)]}_{\mathrm{coronene}\;\mathrm{PD}}\times {[(Q)-(T)]}_{\mathrm{hexacene}}/{[{\left(T\right)}_{\lambda }-\left(T\right)]}_{\mathrm{hexacene}}$$

This results in an estimate of [(*Q*) – (*T*)]_coronene PD_ ≈ 1.37 × 2.87/1.34 = 2.95 kJ/mol. Thus, the CCSDT­(Q)/CBS­(cp-corr) interaction energy for the parallel displaced coronene dimer is estimated as −84.4 kJ/mol. The same estimation for the *T*_3_ – (*T*) contribution yields an interaction energy of −80.8 kJ/mol at the CCSDT/CBS­(cp-corr) level. The same formula for counterpoise uncorrected CCSDT­(Q) and CCSDT interaction energies at the complete basis set provides −81.8 and −77.5 kJ/mol.

Additionally, we can estimate the interaction energy for the even larger sandwich-stacked circumcoronene (*C*_54_ *H*_18_) dimer, where each monomer contains 19 benzene rings.

The CCSD­(T) slope and intercept for the sandwich-structured radially fused polycyclic aromatic hydrocarbons is

y=−9.2×x+2.3kJ/mol

per ring for cp-corrected interaction energy, resulting in an interaction energy of −172.5 kJ/mol for the circumcoronene dimer.

If we extrapolate only the CCSD­(T) correlation energy for the sandwich-structured coronene series and then add the Hartree–Fock energy of the circumcoronene dimer, we obtain (−19.0 ± 0.1) × 19 + (−2.7 ± 0.2) + (165.7 ± 1.4) = (−197.2 ± 3.2) kJ/mol as the cp-corrected result. Meanwhile, the LNO-CCSD­(T)-Tight correlation energy aligns well with a linear trend (see Figure S5 on page 80 in SI), yielding a coefficient of determination *R* ^2^ > 0.999. Our best estimate interaction energy for the sandwich-stacked circumcoronene–circumcoronene dimer at the CCSD­(T)_λ_ level of theory is thus −194.0 ± 3.6 kJ/mol, which is close to the CCSD­(T)/CBS result.

Finally, our CCSD­(T) is very close to the original LNO-CCSD­(T) estimate of ref 10, for which the discrepancy with FN-DMC was originally reported. In ref 16, we estimated the CCSDT­(Q)–CCSD­(T) difference, which comes from higher-order cluster excitations, with a maximum of 3.2 kJ/mol. Here, we can confirm this value with a difference of 3.4 kJ/mol between CCSD­(T) and CCSDT­(Q), estimating it from the deviation of CCSD­(T) to CCSD­(T)_λ_. When going from CCSDT­(Q) to FN-DMC, we still have 7.4 kJ/mol left. Here, FN-DMC is probably underestimating the result because of the fixed-node approximation.^72–75^ This implies that our best estimate for the coronene dimer holds, as our best estimate is in between canonical CCSD­(T) and FN-DMC but somewhat closer to CCSD­(T).

## Conclusions

Extending upon the correlation energy slopes of the alkapolyene (fixed and relaxed geometries) and sandwich-structured polyacene series, we investigated five more series looking at noncovalent interactions. The new series include the minimum, parallel-displaced polyacene structures, as well as the two BN analogues of the sandwich polyacenes and both parallel-displaced and sandwich coronene series structures. With these, we are able to draw a more complete picture of the nature of noncovalent interactions beyond small model systems as their behavior when going to larger sizes is explored. All of the computed series exhibit a near-linear behavior.

By considering local correlation thresholds, basis sets, and different methods, we can draw conclusions about their scaling behavior. For local orbital approximations of the coupled-cluster method, only the tightest threshold settings (vvTight for LNO-CCSD­(T) and DOMOPT = vTight for PNO-LCCSD­(T)) achieve near-canonical-CCSD­(T) accuracy. Overall, the basis set limit for the slopes is much more quickly accomplished than for the intercepts, making calculations of slopes computationally less demanding.

In general, the polyacene and coronene systems behave quite similarly, even for the latter, as the system size grows quadratically (specifically, 3*x* ^2^ – 3*x* + 1) like the number of rings in radially fused polycyclic aromatic hydrocarbons, viz., 1 → 7 → 19 → 37.

Finally, we are able to show that for the parallel-displaced coronene dimer, the higher-order triples are antibonding, which brings CCSDT interaction energies closer to DMC, whereas CCSDT­(Q) tends to get back closer to the CCSD­(T) method, with the final value being closer to the CCSD­(T) than the DMC value, reconfirming our previous results.

## Supplementary Material





## Notes

Full raw data, including additional discussions, extra methods used, and data for further polyaromatic systems (PDF)
Cartesian coordinate (xyz) files of the structures used in this paper (ZIP)
