# Supplementary material for: Intermolecular Interactions of Large Systems: Boron Nitrides, Acenes, and Coronenes
Source: J Phys Chem A. 2026 Aug 26;130(36):7086–98. doi: 10.1021/acs.jpca.6c01896 (PMC13573612; doi:10.1021/acs.jpca.6c01896)
Supplement: Supplementary file 1 [file jp6c01896_si_001.pdf]

Supporting Information: Intermolecular Interactions of Large Systems: Boron Nitrides, Acenes, and Coronenes

Vladimir Fishman,[†] Jan M. L. Martin,[†] and A. Daniel Boese[*,‡]

†*Department of Molecular Chemistry and Materials Science, Weizmann Institute of Science, 7610001 Reḥovot, Israel*

‡*Department of Chemistry, University of Graz, Heinrichstrasse 28/IV, 8010 Graz, Austria*

E-mail: adrian_daniel.boese@uni-graz.at

Contents

Raw CCSD(T) Correlation Data: Basis Set Effects and Local Correlation **S-3**

Raw Hartree-Fock, RPA and DFT-SAPT Data: Basis Set Effects **S-37**

Electronic Structure Methods **S-46**

Assessment of the Results when extrapolating to Larger Molecules **S-61**

Results for Coronene Systems **S-71**

Strong Correlation Diagnostic Dimers **S-79**

HOMO-LUMO gaps for all systems **S-85**

Revised Figure 2c for Previous Paper **S-87**

Examples of input files **S-88**

Raw CCSD(T) Correlation Data: Basis Set Effects and Local Correlation

In this section, all CCSD, CCSD(T), as well as LNO-CCSD, LNO-CCSD(T), DLPNO-CCSD, DLPNO-CCSD(T_1), PNO-LCCSD and PNO-LCCSD(T) raw data for various basis sets cc-pVXZ and aug-cc-pVXZ with X=(D,T,Q,5) is displayed.

Table S1: CCSD(T) correlation part of the interaction energies for different acene parallel displaced dimers for the cc-pVDZ basis set in kJ/mol (cp-corrected).

DZ		CCSD(T) cor	LNO-CCSD(T) cor				DLPNO-CCSD(T_1) Tight		PNO-LCCSD(T)		
System	Rings	canonical	vvTight	vTight	Tight	Normal	TcutPNO=1e-6	TcutPNO=1e-7	DOMOPT Tight	DOMOPT vTight	PAIROPT Tight
c_c	1	-18.28	-18.36	-18.42	-20.03	-21.93	-17.89	-18.36	-17.83	-18.05	-17.60
c2_c2	2	-39.03	-39.35	-39.54	-41.21	-46.58	-38.12	-39.47	-38.15	-38.62	-37.48
c3_c3	3	-60.61	-61.20	-61.55	-64.44	-71.23	-60.04	-62.10	-58.91	-59.73	-57.42
c4_c4	4	-82.74	-83.40	-83.91	-87.93	-98.61	-81.55	-84.88	-80.05	-81.16	-77.85
c5_c5	5	-105.15	-105.94	-106.51	-111.24	-123.96	-103.69	-107.94	-100.54	-102.20	-97.43
c6_c6	6	-127.42	-128.33	-129.10	-134.89	-150.68	-125.99	-130.92	-121.88	-123.77	-118.37

Table S2: CCSD(T) correlation part of the interaction energies for different acene parallel displaced dimers for the aug-cc-pVDZ basis set in kJ/mol (cp-corrected).

aDZ		CCSD(T) cor	LNO-CCSD(T) cor				PNO-LCCSD(T)		
System	Rings	canonical	vvTight	vTight	Tight	Normal	DOMOPT Tight	DOMOPT vTight	PAIROPT Tight
c_c	1	-25.90	-25.94	-25.99	-26.35	-27.82	-25.28	-25.53	-24.40
c2_c2	2	-54.19	-54.35	-54.54	-55.44	-58.87	-52.91	-53.50	-50.07
c3_c3	3	-83.35	-83.80	-84.19	-85.76	-91.40	-80.99	-81.88	-76.52
c4_c4	4	-112.98	-113.55	-113.85	-116.30	-124.41	-110.12	-111.31	-103.37
c5_c5	5			-144.53	-147.95	-158.33			
c6_c6	6			-174.67	-178.51	-192.35			

Table S3: CCSD(T) correlation part of the interaction energies for different acene parallel displaced dimers for the cc-pVTZ basis set in kJ/mol (cp-corrected).

TZ		CCSD(T) cor	LNO-CCSD(T) cor				DLPNO-CCSD(T_1) Tight		PNO-LCCSD(T)		
System	Rings	canonical	vvTight	vTight	Tight	Normal	TcutPNO=1e-6	TcutPNO=1e-7	DOMOPT Tight	DOMOPT vTight	PAIROPT Tight
c_c	1	-23.87	-24.17	-24.22	-26.03	-27.36	-27.14	-27.46	-23.44	-23.61	-23.36
c2_c2	2	-50.53	-51.14	-51.30	-53.00	-58.31	-48.82	-50.37	-49.36	-49.82	-48.69
c3_c3	3	-78.08	-79.19	-79.45	-82.17	-90.44	-75.86	-78.51	-75.92	-76.65	-75.18
c4_c4	4	-106.10	-107.56	-107.98	-112.03	-123.22	-103.33	-107.05	-103.48	-104.42	-101.73
c5_c5	5			-136.75	-142.58	-157.22		-136.06			
c6_c6	6			-165.81	-172.79	-190.64		-164.78			

Table S4: CCSD(T) correlation part of the interaction energies for different acene parallel displaced dimers for the aug-cc-pVTZ basis set in kJ/mol(cp-corrected).

aTZ		CCSD(T) cor	LNO-CCSD(T) cor			
System	Rings	canonical	vvTight	vTight	Tight	Normal
c_c	1	-26.98	-27.11	-27.12	-27.45	-28.81
c2_c2	2	-56.09	-56.47	-56.64	-57.33	-60.71
c3_c3	3			-87.11	-88.53	-94.04
c4_c4	4			-117.99	-120.32	-128.46
c5_c5	5				-153.14	-162.87
c6_c6	6			-181.66	-185.16	-198.22

Table S5: CCSD(T) correlation part of the interaction energies for different acene parallel displaced dimers for the cc-pVQZ basis set in kJ/mol(cp-corrected).

QZ		CCSD(T) cor	LNO-CCSD(T) cor				DLPNO-CCSD(T_1) Tight	
System	Rings	canonical	vvTight	vTight	Tight	Normal	TcutPNO=1e-6	TcutPNO=1e-7
c_c	1	-26.03	-26.20	-26.28	-26.83	-28.88	-25.33	-25.79
c2_c2	2	-54.57	-54.92	-55.15	-56.64	-61.30	-52.76	
c3_c3	3			-85.01	-87.37	-93.86		
c4_c4	4			-115.38	-118.62	-128.03		
c5_c5	5			-146.70	-150.69	-163.12		
c6_c6	6			-177.44	-182.36	-197.65		

Table S6: CCSD(T) correlation part of the interaction energies for different acene parallel displaced dimers for the aug-cc-pVQZ basis set in kJ/mol(cp-corrected).

aQZ		CCSD(T) cor	LNO-CCSD(T) cor			
System	Rings	canonical	vvTight	vTight	Tight	Normal
c_c	1	-27.28	-27.51	-27.56	-27.83	-29.42
c2_c2	2		-57.33	-57.15	-58.09	-61.73
c3_c3	3			-87.67	-89.36	-95.26
c4_c4	4			-118.79	-121.28	-129.42
c5_c5	5				-153.98	-164.84
c6_c6	6				-186.29	-200.18

Table S7: CCSD(T) correlation part of the interaction energies for different acene parallel displaced dimers for the cc-pV5Z basis set in kJ/mol(cp-corrected).

5Z		CCSD(T) cor	LNO-CCSD(T) cor			
System	Rings	canonical	vvTight	vTight	Tight	Normal
c_c	1	-26.97	-27.11	-27.15	-27.76	-29.51
c2_c2	2		-56.54	-56.77	-58.38	-62.00
c3_c3	3			-87.32	-89.64	-95.70
c4_c4	4			-118.38	-121.66	-130.16
c5_c5	5				-154.14	-164.81
c6_c6	6				-186.47	-200.21

Table S8: CCSD(T) correlation part of the interaction energies for different acene parallel displaced dimers for the aug-cc-pV5Z basis set in kJ/mol(cp-corrected).

a5Z		CCSD(T) cor	LNO-CCSD(T) cor			
System	Rings	canonical	vvTight	vTight	Tight	Normal
c_c	1	-27.36	-27.51	-27.61	-27.99	-29.42
c2_c2	2		-57.15	-57.41	-58.42	-61.72
c3_c3	3			-88.05	-89.67	-95.36
c4_c4	4			-119.39	-121.79	-130.16
c5_c5	5				-154.27	-165.07
c6_c6	6				-186.72	-200.54

Table S9: CCSD correlation part of the interaction energies for different acene parallel displaced dimers for the cc-pVDZ basis set in kJ/mol(cp-corrected).

DZ		CCSD cor	LNO-CCSD cor				DLPNO-CCSD Tight		PNO-LCCSD		
System	Rings	canonical	vvTight	vTight	Tight	Normal	TcutPNO=1e-6	TcutPNO=1e-7	DOMOPT Tight	DOMOPT vTight	PAIROPT Tight
c_c	1	-15.55	-15.74	-15.79	-16.94	-35.04	-15.82	-15.93	-15.33	-15.44	-15.23
c2_c2	2	-32.77	-33.33	-33.42	-34.27	-50.52	-33.74	-34.10	-32.51	-32.74	-32.14
c3_c3	3	-50.55	-51.48	-51.64	-53.08	-53.80	-53.29	-53.67	-50.13	-50.49	-49.18
c4_c4	4	-68.72	-69.82	-70.01	-71.55	-73.83	-72.75	-73.62	-68.04	-68.52	-66.60
c5_c5	5	-87.08	-88.39	-88.54	-90.40	-92.51	-92.80	-93.83	-85.75	-86.33	-83.59
c6_c6	6	-105.28	-106.86	-107.04	-109.24	-112.07	-112.93	-114.03	-103.81	-104.55	-101.48

Table S10: CCSD correlation part of the interaction energies for different acene parallel displaced dimers for the aug-cc-pVDZ basis set in kJ/mol(cp-corrected).

aDZ		CCSD cor	LNO-CCSD cor				PNO-LCCSD		
System	Rings	canonical	vvTight	vTight	Tight	Normal	DOMOPT Tight	DOMOPT vTight	PAIROPT Tight
c_c	1	-21.53	-21.79	-21.90	-21.91	-21.60	-21.26	-21.38	-20.69
c2_c2	2	-44.57	-45.17	-45.37	-45.50	-45.08	-44.19	-44.46	-42.22
c3_c3	3	-68.16	-69.24	-69.55	-69.79	-69.42	-67.49	-67.89	-64.37
c4_c4	4	-92.08	-93.43	-93.56	-94.03	-94.02	-91.70	-92.22	-86.84
c5_c5	5			-118.39	-119.18	-118.96			
c6_c6	6			-142.75	-143.34	-143.78			

Table S11: CCSD correlation part of the interaction energies for different acene parallel displaced dimers for the cc-pVTZ basis set in kJ/mol(cp-corrected).

TZ		CCSD cor	LNO-CCSD cor				DLPNO-CCSD Tight		PNO-LCCSD		
System	Rings	canonical	vvTight	vTight	Tight	Normal	TcutPNO=1e-6	TcutPNO=1e-7	DOMOPT Tight	DOMOPT vTight	PAIROPT Tight
c_c	1	-19.77	-20.29	-20.37	-23.15	-21.11	-25.45	-25.42	-19.68	-19.70	-19.72
c2_c2	2	-41.42	-42.51	-42.63	-43.32	-44.60	-42.46	-42.52	-41.19	-41.28	-40.90
c3_c3	3	-63.65	-65.48	-65.65	-66.74	-68.45	-66.20	-66.36	-63.24	-63.39	-63.04
c4_c4	4	-86.20	-88.62	-88.88	-90.43	-92.79	-90.45	-90.71	-86.04	-86.20	-85.12
c5_c5	5			-112.24	-114.83	-118.06		-115.47			
c6_c6	6			-135.71	-138.74	-143.08		-140.30			

Table S12: CCSD correlation part of the interaction energies for different acene parallel displaced dimers for the aug-cc-pVTZ basis set in kJ/mol(cp-corrected).

aTZ		CCSD cor	LNO-CCSD cor			
System	Rings	canonical	vvTight	vTight	Tight	Normal
c_c	1	-22.11	-22.52	-22.58	-22.56	-22.09
c2_c2	2	-45.57	-46.51	-46.68	-46.56	-46.11
c3_c3	3			-71.31	-71.34	-70.79
c4_c4	4			-96.18	-96.42	-96.18
c5_c5	5			-121.90	-122.43	-121.78
c6_c6	6			-147.29	-147.67	-147.58

Table S13: CCSD correlation part of the interaction energies for different acene parallel displaced dimers for the cc-pVQZ basis set in kJ/mol(cp-corrected).

QZ		CCSD cor	LNO-CCSD cor				DLPNO-CCSD Tight	
System	Rings	canonical	vvTight	vTight	Tight	Normal	TcutPNO=1e-6	TcutPNO=1e-7
c_c	1	-21.36	-21.82	-21.91	-22.10	-22.01	-21.87	-21.69
c2_c2	2	-44.35	-45.29	-45.47	-45.93	-46.15	-45.60	
c3_c3	3			-69.65	-70.28	-70.63		
c4_c4	4			-94.14	-94.99	-95.86		
c5_c5	5			-119.00	-120.31	-121.52		
c6_c6	6			-143.68	-145.30	-146.93		

Table S14: CCSD correlation part of the interaction energies for different acene parallel displaced dimers for the aug-cc-pVQZ basis set in kJ/mol(cp-corrected).

aQZ		CCSD cor	LNO-CCSD cor			
System	Rings	canonical	vvTight	vTight	Tight	Normal
c_c	1	-22.29	-22.81	-22.88	-22.81	-22.47
c2_c2	2		-47.12	-46.94	-47.00	-46.71
c3_c3	3			-71.58	-71.75	-71.48
c4_c4	4			-96.59	-96.84	-96.86
c5_c5	5				-122.65	-122.97
c6_c6	6				-148.00	-148.79

Table S15: CCSD correlation part of the interaction energies for different acene parallel displaced dimers for the cc-pV5Z basis set in kJ/mol(cp-corrected).

5Z		CCSD cor	LNO-CCSD cor			
System	Rings	canonical	vvTight	vTight	Tight	Normal
c_c	1	-22.05	-22.50	-22.52	-22.60	-22.27
c2_c2	2		-46.46	-46.61	-47.05	-46.75
c3_c3	3			-71.26	-71.75	-71.83
c4_c4	4			-96.22	-96.99	-97.05
c5_c5	5				-122.48	-122.49
c6_c6	6				-147.83	-148.28

Table S16: CCSD correlation part of the interaction energies for different acene parallel displaced dimers for the aug-cc-pV5Z basis set in kJ/mol(cp-corrected).

a5Z		CCSD cor	LNO-CCSD cor			
System	Rings	canonical	vvTight	vTight	Tight	Normal
c_c	1	-22.34	-22.78	-22.89	-22.88	-22.51
c2_c2	2		-46.90	-47.07	-47.28	-46.80
c3_c3	3			-71.77	-71.97	-71.62
c4_c4	4			-96.20	-97.20	-97.34
c5_c5	5				-122.94	-123.06
c6_c6	6				-148.41	-148.78

Table S17: CCSD(T) correlation part of the interaction energies for different BN_NB dimers for the cc-pVDZ basis set in kJ/mol (cp-corrected).

DZ		CCSD(T) cor	LNO-CCSD(T) cor				DLPNO-CCSD(T_1) Tight		PNO-LCCSD(T)		
System	Rings	canonical	vvTight	vTight	Tight	Normal	TcutPNO=1e-6	TcutPNO=1e-7	DOMOPT Tight	DOMOPT vTight	PAIROPT Tight
bn_nb	1	-13.04	-13.12	-13.07	-15.39	-15.78	-12.03	-12.78	-12.94	-13.28	-12.64
bn2_nb2	2	-26.01	-26.01	-25.95	-28.08	-30.52	-24.55	-25.79	-25.39	-26.04	-25.02
bn3_nb3	3	-39.60	-39.59	-39.54	-42.47	-47.20	-37.54	-39.79	-38.68	-39.74	-37.92
bn4_nb4	4	-53.53	-54.12	-54.04	-57.33	-64.64	-51.09	-54.03	-52.03	-53.54	-51.03
bn5_nb5	5	-64.58	-65.49	-65.39	-69.93	-78.18	-62.42	-65.51	-62.67	-64.59	-61.26
bn6_nb6	6	-78.88	-79.01	-78.88	-84.14	-95.63	-76.16	-80.02	-76.74	-79.08	-75.09

Table S18: CCSD(T) correlation part of the interaction energies for different BN_NB dimers for the aug-cc-pVDZ basis set in kJ/mol (cp-corrected).

aDZ		CCSD(T) cor	LNO-CCSD(T) cor				DLPNO-CCSD(T_1) Tight		PNO-LCCSD(T)		
System	Rings	canonical	vvTight	vTight	Tight	Normal	TcutPNO=1e-6	TcutPNO=1e-7	DOMOPT Tight	DOMOPT vTight	PAIROPT Tight
bn_nb	1	-18.05	-17.95	-17.82	-18.14	-19.29	-16.48	-17.40	-17.41	-17.72	-17.11
bn2_nb2	2	-34.68	-34.34	-54.77	-34.69	-36.41	-32.35	-34.00	-33.60	-34.41	-32.83
bn3_nb3	3	-52.02	-51.49	-51.24	-52.08	-54.76	-49.15	-51.82	-50.45	-51.70	-48.84
bn4_nb4	4		-68.94	-68.57	-70.20	-73.74	-66.33	-70.13	-67.66	-69.36	-65.27
bn5_nb5	5			-82.31	-84.34	-89.03	-80.80	-85.22			
bn6_nb6	6			-101.56	-103.98	-109.03	-98.48	-103.78			

Table S19: CCSD(T) correlation part of the interaction energies for different BN_NB dimers for the cc-pVTZ basis set in kJ/mol (cp-corrected).

TZ		CCSD(T) cor	LNO-CCSD(T) cor				DLPNO-CCSD(T_1) Tight		PNO-LCCSD(T)		
System	Rings	canonical	vvTight	vTight	Tight	Normal	TcutPNO=1e-6	TcutPNO=1e-7	DOMOPT Tight	DOMOPT vTight	PAIROPT Tight
bn_nb	1	-17.09	-16.91	-16.78	-17.53	-18.83	-16.02	-16.61	-16.69	-17.01	-16.35
bn2_nb2	2	-33.14	-32.95	-32.82	-34.47	-37.03	-31.42	-32.78	-32.17	-32.90	-31.52
bn3_nb3	3	-49.89	-49.66	-49.55	-51.98	-55.57	-47.07	-49.71	-47.70	-49.05	-46.06
bn4_nb4	4	-67.05	-66.83	-66.72	-70.49	-74.96	-64.16	-67.48	-64.77	-66.38	-63.00
bn5_nb5	5			-80.15	-84.08	-90.33	-77.26				
bn6_nb6	6			-97.85	-102.48	-110.29	-94.34				

Table S20: CCSD(T) correlation part of the interaction energies for different BN_NB dimers for the aug-cc-pVTZ basis set in kJ/mol (cp-corrected).

aTZ		CCSD(T) cor	LNO-CCSD(T) cor				DLPNO-CCSD(T_1) Tight	
System	Rings	canonical	vvTight	vTight	Tight	Normal	TcutPNO=1e-6	TcutPNO=1e-7
bn_nb	1	-19.17	-18.98	-18.89	-19.05	-19.87	-17.78	-18.61
bn2_nb2	2	-36.49	-36.15	-35.99	-36.56	-37.90	-34.05	-35.67
bn3_nb3	3		-54.06	-53.79	-54.40	-56.81	-51.38	-53.96
bn4_nb4	4			-71.90	-73.26	-76.33		-72.31
bn5_nb5	5			-86.12	-88.12	-91.80		-87.42
bn6_nb6	6			-105.14	-107.32	-111.56		

Table S21: CCSD(T) correlation part of the interaction energies for different BN_NB dimers for the cc-pVQZ basis set in kJ/mol (cp-corrected).

QZ		CCSD(T) cor	LNO-CCSD(T) cor				DLPNO-CCSD(T_1) Tight	
System	Rings	canonical	vvTight	vTight	Tight	Normal	TcutPNO=1e-6	TcutPNO=1e-7
bn_nb	1	-18.57	-18.43	-18.27	-18.95	-19.59	-17.29	-17.94
bn2_nb2	2	-35.59	-35.28	-35.16	-36.37	-37.86	-33.15	-34.74
bn3_nb3	3		-52.87	-52.74	-54.60	-57.14	-49.71	-52.21
bn4_nb4	4			-70.81	-73.36	-76.61	-66.88	
bn5_nb5	5			-87.96	-88.15	-92.31		
bn6_nb6	6			-104.29	-107.51	-112.39		

Table S22: CCSD(T) correlation part of the interaction energies for different BN_NB dimers for the aug-cc-pVQZ basis set in kJ/mol (cp-corrected).

aQZ		CCSD(T) cor	LNO-CCSD(T) cor				DLPNO-CCSD(T_1) Tight	
System	Rings	canonical	vvTight	vTight	Tight	Normal	TcutPNO=1e-6	TcutPNO=1e-7
bn_nb	1	-19.32	-19.25	-19.29	-19.34	-19.84	-17.98	-18.82
bn2_nb2	2			-36.68	-36.80	-38.50	-34.27	-35.95
bn3_nb3	3			-54.91	-55.26	-57.60		
bn4_nb4	4			-73.57	-73.86	-77.72		
bn5_nb5	5				-88.77	-92.86		
bn6_nb6	6				-108.15	-113.12		

Table S23: CCSD(T) correlation part of the interaction energies for different BN_NB dimers for the cc-pV5Z basis set in kJ/mol (cp-corrected).

5Z		CCSD(T) cor	LNO-CCSD(T) cor				DLPNO-CCSD(T_1) Tight	
System	Rings	canonical	vvTight	vTight	Tight	Normal	TcutPNO=1e-6	TcutPNO=1e-7
bn_nb	1	-19.11	-18.95	-18.84	-19.24	-20.28	-17.73	-18.52
bn2_nb2	2		-36.40	-36.04	-36.81	-38.70		
bn3_nb3	3			-53.92	-54.93	-58.23		
bn4_nb4	4			-72.21	-73.67	-78.13		
bn5_nb5	5				-88.32	-93.89		
bn6_nb6	6				-107.64	-114.33		

Table S24: CCSD(T) correlation part of the interaction energies for different BN_NB dimers for the aug-cc-pV5Z basis set in kJ/mol (cp-corrected).

a5Z		CCSD(T) cor	LNO-CCSD(T) cor				DLPNO-CCSD(T_1) Tight	
System	Rings	canonical	vvTight	vTight	Tight	Normal	TcutPNO=1e-6	TcutPNO=1e-7
bn_nb	1		-19.31	-19.18	-19.42	-20.36	-18.22	-18.82
bn2_nb2	2			-36.55	-37.20	-38.50		
bn3_nb3	3			-54.50	-55.52	-57.69		
bn4_nb4	4			-73.02	-74.16	-77.26		
bn5_nb5	5				-89.14	-93.71		
bn6_nb6	6				-108.49	-113.00		

Table S25: CCSD correlation part of the interaction energies for different BN_NB dimers for the cc-pVDZ basis set in kJ/mol (cp-corrected).

DZ		CCSD cor	LNO-CCSD cor				DLPNO-CCSD Tight		PNO-LCCSD		
System	Rings	canonical	vvTight	vTight	Tight	Normal	TcutPNO=1e-6	TcutPNO=1e-7	DOMOPT Tight	DOMOPT vTight	PAIROPT Tight
bn1_nb1	1	-11.00	-11.33	-11.26	-12.62	-11.63	-10.63	-11.08	-11.07	-11.26	-10.82
bn2_nb2	2	-21.84	-22.41	-22.18	-22.89	-22.54	-21.78	-22.47	-21.63	-22.06	-21.34
bn3_nb3	3	-33.19	-33.98	-33.62	-34.50	-34.32	-33.38	-34.69	-33.00	-33.68	-32.37
bn4_nb4	4	-44.81	-46.34	-45.85	-46.31	-47.25	-45.48	-47.13	-44.42	-45.37	-43.60
bn5_nb5	5	-54.04	-55.98	-55.30	-56.35	-56.58	-55.73	-57.35	-53.56	-54.75	-52.40
bn6_nb6	6	-65.97	-67.49	-66.66	-67.66	-69.20	-67.99	-70.01	-65.63	-67.07	-64.28

Table S26: CCSD correlation part of the interaction energies for different BN_NB dimers for the aug-cc-pVDZ basis set in kJ/mol (cp-corrected).

aDZ		CCSD cor	LNO-CCSD cor				DLPNO-CCSD Tight		PNO-LCCSD		
System	Rings	canonical	vvTight	vTight	Tight	Normal	TcutPNO=1e-6	TcutPNO=1e-7	DOMOPT Tight	DOMOPT vTight	PAIROPT Tight
bn1_nb1	1	-14.98	-15.22	-15.15	-15.03	-13.77	-14.20	-14.74	-14.57	-14.78	-14.43
bn2_nb2	2	-28.67	-28.99	-46.67	-28.20	-25.80	-27.99	-28.91	-28.13	-28.68	-27.60
bn3_nb3	3	-42.94	-43.36	-42.97	-41.78	-38.53	-42.61	-44.16	-42.26	-43.12	-41.11
bn4_nb4	4		-57.92	-57.27	-56.11	-51.71	-57.63	-59.86	-56.71	-57.87	-54.97
bn5_nb5	5			-68.46	-66.92	-62.10	-70.42	-72.94			
bn6_nb6	6			-84.46	-83.11	-78.00	-85.90	-88.89			

Table S27: CCSD correlation part of the interaction energies for different BN_NB dimers for the cc-pVTZ basis set in kJ/mol (cp-corrected).

TZ		CCSD cor	LNO-CCSD cor				DLPNO-CCSD Tight		PNO-LCCSD		
System	Rings	canonical	vvTight	vTight	Tight	Normal	TcutPNO=1e-6	TcutPNO=1e-7	DOMOPT Tight	DOMOPT vTight	PAIROPT Tight
bn1_nb1	1	-14.10	-14.30	-14.23	-14.38	-13.41	-13.87	-14.07	-13.91	-14.10	-13.69
bn2_nb2	2	-27.25	-27.79	-27.57	-27.84	-26.18	-27.26	-27.94	-26.79	-27.25	-26.35
bn3_nb3	3	-40.96	-41.81	-41.47	-41.81	-39.41	-41.02	-42.47	-39.96	-40.68	-38.76
bn4_nb4	4	-55.01	-56.18	-55.69	-56.44	-53.38	-56.01	-57.81	-54.09	-55.05	-52.81
bn5_nb5	5			-66.76	-67.07	-63.80	-67.67				
bn6_nb6	6			-81.42	-81.64	-77.99	-82.66				

Table S28: CCSD correlation part of the interaction energies for different BN_NB dimers for the aug-cc-pVTZ basis set in kJ/mol (cp-corrected).

aTZ		CCSD cor	LNO-CCSD cor				DLPNO-CCSD Tight	
System	Rings	canonical	vvTight	vTight	Tight	Normal	TcutPNO=1e-6	TcutPNO=1e-7
bn1_nb1	1	-15.68	-15.90	-15.86	-15.61	-14.00	-15.24	-15.69
bn2_nb2	2	-29.79	-30.22	-30.05	-29.43	-26.81	-29.32	-30.13
bn3_nb3	3		-45.11	-44.67	-43.34	-39.74	-44.46	-45.78
bn4_nb4	4			-59.51	-58.06	-53.49		-61.37
bn5_nb5	5			-71.12	-69.51	-64.08		-74.38
bn6_nb6	6			-86.73	-84.59	-77.89		

Table S29: CCSD correlation part of the interaction energies for different BN_NB dimers for the cc-pVQZ basis set in kJ/mol (cp-corrected).

QZ		CCSD cor	LNO-CCSD cor				DLPNO-CCSD Tight	
System	Rings	canonical	vvTight	vTight	Tight	Normal	TcutPNO=1e-6	TcutPNO=1e-7
bn1_nb1	1	-15.20	-15.45	-15.35	-15.43	-14.15	-14.82	-15.09
bn2_nb2	2	-29.04	-29.54	-29.36	-29.32	-26.72	-28.65	-29.40
bn3_nb3	3		-44.19	-43.87	-43.61	-40.23	-43.07	-44.29
bn4_nb4	4			-58.73	-58.36	-53.91	-58.06	
bn5_nb5	5			-77.57	-69.71	-64.40		
bn6_nb6	6			-85.90	-84.97	-78.67		

Table S30: CCSD correlation part of the interaction energies for different BN_NB dimers for the aug-cc-pVQZ basis set in kJ/mol (cp-corrected).

aQZ		CCSD cor	LNO-CCSD cor				DLPNO-CCSD Tight	
System	Rings	canonical	vvTight	vTight	Tight	Normal	TcutPNO=1e-6	TcutPNO=1e-7
bn1_nb1	1	-15.76	-16.08	-16.06	-15.73	-14.15	-15.44	-15.83
bn2_nb2	2			-30.39	-29.56	-27.31	-29.50	-30.35
bn3_nb3	3			-45.33	-44.05	-40.56		
bn4_nb4	4			-60.58	-58.67	-54.61		
bn5_nb5	5				-70.10	-64.55		
bn6_nb6	6				-85.35	-78.95		

Table S31: CCSD correlation part of the interaction energies for different BN_NB dimers for the cc-pV5Z basis set in kJ/mol (cp-corrected).

5Z		CCSD cor	LNO-CCSD cor				DLPNO-CCSD Tight	
System	Rings	canonical	vvTight	vTight	Tight	Normal	TcutPNO=1e-6	TcutPNO=1e-7
bn1_nb1	1	-15.59	-15.84	-15.77	-15.56	-14.44	-15.19	-15.55
bn2_nb2	2		-30.16	-29.99	-29.55	-27.14		
bn3_nb3	3			-44.68	-43.62	-41.03		
bn4_nb4	4			-59.69	-58.38	-54.53		
bn5_nb5	5				-69.72	-64.74		
bn6_nb6	6				-84.83	-79.01		

Table S32: CCSD correlation part of the interaction energies for different BN_NB dimers for the aug-cc-pV5Z basis set in kJ/mol (cp-corrected).

a5Z		CCSD cor	LNO-CCSD cor				DLPNO-CCSD Tight	
System	Rings	canonical	vvTight	vTight	Tight	Normal	TcutPNO=1e-6	TcutPNO=1e-7
bn1_nb1	1		-16.10	-16.03	-15.78	-14.45	-15.60	-15.78
bn2_nb2	2			-30.34	-29.92	-27.58		
bn3_nb3	3			-45.08	-44.18	-40.63		
bn4_nb4	4			-60.25	-58.76	-54.29		
bn5_nb5	5				-70.35	-65.45		
bn6_nb6	6				-85.50	-78.92		

Table S33: CCSD(T) correlation part of the interaction energies for different BN_BN dimers for the cc-pVDZ basis set in kJ/mol (cp-corrected).

DZ		CCSD(T) cor	LNO-CCSD(T) cor				DLPNO-CCSD(T_1) Tight		PNO-LCCSD(T)		
System	Rings	canonical	vvTight	vTight	Tight	Normal	TcutPNO=1e-6	TcutPNO=1e-7	DOMOPT Tight	DOMOPT vTight	PAIROPT Tight
bn1_bn1	1	-16.71	-16.58	-16.40	-19.48	-21.16	-15.19	-15.95	-16.63	-16.97	-16.51
bn2_bn2	2	-36.40	-36.11	-35.86	-38.91	-42.51	-33.57	-35.89	-34.52	-35.97	-31.52
bn3_bn3	3	-57.18	-56.86	-56.52	-60.75	-66.68	-52.83	-56.69	-55.03	-57.01	-51.10
bn4_bn4	4	-78.07	-77.61	-77.14	-82.28	-92.20	-72.21	-77.52	-75.81	-78.23	-71.05
bn5_bn5	5	-99.00	-98.71	-98.13	-104.18	-117.11	-91.93	-98.71	-96.46	-99.32	-91.04
bn6_bn6	6	-120.01	-119.64	-118.96	-126.87	-138.13	-111.53	-119.84	-117.21	-120.52	-111.09

Table S34: CCSD(T) correlation part of the interaction energies for different BN_BN dimers for the aug-cc-pVDZ basis set in kJ/mol (cp-corrected).

aDZ		CCSD(T) cor	LNO-CCSD(T) cor				DLPNO-CCSD(T_1) Tight		PNO-LCCSD(T)		
System	Rings	canonical	vvTight	vTight	Tight	Normal	TcutPNO=1e-6	TcutPNO=1e-7	DOMOPT Tight	DOMOPT vTight	PAIROPT Tight
bn1_bn1	1	-24.03	-23.76	-23.54	-23.42	-24.35	-21.67	-22.88	-23.56	-23.91	-22.45
bn2_bn2	2	-49.09	-48.66	-48.33	-48.13	-49.65	-45.00	-47.62	-46.59	-47.51	-42.04
bn3_bn3	3	-75.15	-74.48	-74.00	-74.16	-76.90	-70.07	-74.18	-72.07	-73.60	-65.41
bn4_bn4	4		-100.10	-99.47	-99.95	-104.36	-94.62	-100.39	-97.54	-99.55	-88.67
bn5_bn5	5			-125.29	-126.15	-131.40	-119.57	-126.71			
bn6_bn6	6			-151.04	-152.43	-158.38	-143.93	-153.76			

Table S35: CCSD(T) correlation part of the interaction energies for different BN_BN dimers for the cc-pVTZ basis set in kJ/mol (cp-corrected).

TZ		CCSD(T) cor	LNO-CCSD(T) cor				DLPNO-CCSD(T_1) Tight		PNO-LCCSD(T)		
System	Rings	canonical	vvTight	vTight	Tight	Normal	TcutPNO=1e-6	TcutPNO=1e-7	DOMOPT Tight	DOMOPT vTight	PAIROPT Tight
bn1_bn1	1	-23.32	-22.99	-22.76	-24.24	-25.55	-21.24	-22.26	-22.83	-23.14	-22.36
bn2_bn2	2	-48.12	-47.63	-47.36	-48.47	-51.64	-44.31	-46.83	-46.49	-47.49	-44.83
bn3_bn3	3	-73.82	-73.41	-73.03	-74.39	-80.49	-68.18	-72.32	-71.89	-73.21	-70.00
bn4_bn4	4	-99.52	-99.11	-98.67	-100.84	-108.46	-91.88	-97.57	-96.88	-98.64	-94.20
bn5_bn5	5			-124.36	-127.06	-136.37	-115.91	-122.94			
bn6_bn6	6			-150.15	-152.78	-164.67	-139.56	-148.12			

Table S36: CCSD(T) correlation part of the interaction energies for different BN_BN dimers for the aug-cc-pVTZ basis set in kJ/mol (cp-corrected).

aTZ		CCSD(T) cor	LNO-CCSD(T) cor				DLPNO-CCSD(T_1) Tight	
System	Rings	canonical	vvTight	vTight	Tight	Normal	TcutPNO=1e-6	TcutPNO=1e-7
bn1_bn1	1	-26.24	-25.98	-25.77	-25.73	-26.57	-23.92	-25.12
bn2_bn2	2	-52.98	-52.48	-52.15	-52.26	-53.47	-48.94	-51.43
bn3_bn3	3		-79.82	-79.25	-79.40	-81.96	-74.54	-78.65
bn4_bn4	4			-106.36	-107.05	-110.77		-105.78
bn5_bn5	5			-133.57	-134.25	-138.81		-133.20
bn6_bn6	6			-161.10	-161.67	-167.33		

Table S37: CCSD(T) correlation part of the interaction energies for different BN_BN dimers for the cc-pVQZ basis set in kJ/mol (cp-corrected).

QZ		CCSD(T) cor	LNO-CCSD(T) cor				DLPNO-CCSD(T_1) Tight	
System	Rings	canonical	vvTight	vTight	Tight	Normal	TcutPNO=1e-6	TcutPNO=1e-7
bn1_bn1	1	-25.55	-24.50	-25.05	-25.99	-26.90	-23.22	-24.39
bn2_bn2	2	-51.98	-51.47	-51.20	-51.96	-54.59	-47.68	-50.27
bn3_bn3	3		-78.51	-78.21	-79.32	-83.09	-72.74	-76.83
bn4_bn4	4			-105.21	-106.41	-111.73	-97.90	
bn5_bn5	5			-132.19	-133.96	-140.37		
bn6_bn6	6			-160.38	-161.58	-169.30		

Table S38: CCSD(T) correlation part of the interaction energies for different BN_BN dimers for the aug-cc-pVQZ basis set in kJ/mol (cp-corrected).

aQZ		CCSD(T) cor	LNO-CCSD(T) cor				DLPNO-CCSD(T_1) Tight	
System	Rings	canonical	vvTight	vTight	Tight	Normal	TcutPNO=1e-6	TcutPNO=1e-7
bn1_bn1	1	-26.67	-26.48	-26.21	-26.29	-27.15	-24.36	-25.73
bn2_bn2	2			-53.04	-53.28	-55.08	-49.71	-52.14
bn3_bn3	3			-80.65	-81.05	-83.70		
bn4_bn4	4			-108.16	-109.07	-112.76		
bn5_bn5	5				-136.92	-141.92		
bn6_bn6	6				-164.74	-170.67		

Table S39: CCSD(T) correlation part of the interaction energies for different BN_BN dimers for the cc-pV5Z basis set in kJ/mol (cp-corrected).

5Z		CCSD(T) cor	LNO-CCSD(T) cor			
System	Rings	canonical	vvTight	vTight	Tight	Normal
bn1_bn1	1	-26.42	-26.25	-26.04	-26.08	-27.12
bn2_bn2	2		-53.24	-52.73	-53.15	-54.85
bn3_bn3	3			-80.01	-80.84	-83.96
bn4_bn4	4			-107.63	-108.65	-112.74
bn5_bn5	5				-136.37	-141.66
bn6_bn6	6				-164.30	-170.86

Table S40: CCSD(T) correlation part of the interaction energies for different BN_BN dimers for the aug-cc-pV5Z basis set in kJ/mol (cp-corrected).

a5Z		CCSD(T) cor	LNO-CCSD(T) cor			
System	Rings	canonical	vvTight	vTight	Tight	Normal
bn1_bn1	1		-26.63	-26.42	-26.42	-27.26
bn2_bn2	2			-53.34	-53.47	-55.25
bn3_bn3	3			-81.06	-81.51	-84.43
bn4_bn4	4			-108.85	-109.52	-113.61
bn5_bn5	5				-137.33	-142.80
bn6_bn6	6				-165.53	-171.35

Table S41: CCSD correlation part of the interaction energies for different BN_BN dimers for the cc-pVDZ basis set in kJ/mol (cp-corrected).

DZ		CCSD cor	LNO-CCSD cor				DLPNO-CCSD Tight		PNO-LCCSD		
System	Rings	canonical	vvTight	vTight	Tight	Normal	TcutPNO=1e-6	TcutPNO=1e-7	DOMOPT Tight	DOMOPT vTight	PAIROPT Tight
bn1_bn1	1	-13.97	-14.14	-14.06	-16.32	-15.94	-13.32	-13.69	-14.12	-14.31	-14.03
bn2_bn2	2	-30.33	-30.70	-30.47	-33.04	-31.89	-29.56	-30.85	-29.86	-30.29	-27.42
bn3_bn3	3	-47.56	-48.23	-47.87	-51.05	-50.05	-46.56	-48.77	-47.25	-47.98	-44.02
bn4_bn4	4	-64.87	-65.79	-65.25	-68.90	-69.21	-63.67	-66.76	-64.86	-65.82	-60.96
bn5_bn5	5	-82.21	-83.57	-82.90	-87.11	-87.92	-81.11	-85.06	-82.37	-83.57	-77.92
bn6_bn6	6	-99.62	-101.22	-100.39	-105.92	-104.06	-98.42	-103.35	-99.96	-101.41	-94.95

Table S42: CCSD correlation part of the interaction energies for different BN_BN dimers for the aug-cc-pVDZ basis set in kJ/mol (cp-corrected).

aDZ		CCSD cor	LNO-CCSD cor				DLPNO-CCSD Tight		PNO-LCCSD		
System	Rings	canonical	vvTight	vTight	Tight	Normal	TcutPNO=1e-6	TcutPNO=1e-7	DOMOPT Tight	DOMOPT vTight	PAIROPT Tight
bn1_bn1	1	-19.95	-20.04	-19.98	-19.58	-18.43	-18.74	-19.38	-19.76	-19.97	-18.96
bn2_bn2	2	-40.62	-40.93	-40.75	-39.83	-37.08	-39.07	-40.49	-39.13	-39.63	-35.61
bn3_bn3	3	-62.08	-62.48	-62.17	-60.98	-57.26	-60.89	-63.11	-60.48	-61.40	-55.33
bn4_bn4	4		-83.80	-83.22	-81.63	-77.31	-82.27	-85.44	-81.83	-83.05	-74.94
bn5_bn5	5			-104.54	-102.93	-97.13	-104.08	-107.92			
bn6_bn6	6			-125.94	-124.31	-117.01	-125.40	-131.04			

Table S43: CCSD correlation part of the interaction energies for different BN_BN dimers for the cc-pVTZ basis set in kJ/mol (cp-corrected).

TZ		CCSD cor	LNO-CCSD cor				DLPNO-CCSD Tight		PNO-LCCSD		
System	Rings	canonical	vvTight	vTight	Tight	Normal	TcutPNO=1e-6	TcutPNO=1e-7	DOMOPT Tight	DOMOPT vTight	PAIROPT Tight
bn1_bn1	1	-19.29	-19.43	-19.37	-20.22	-19.30	-18.47	-18.87	-19.11	-19.26	-18.81
bn2_bn2	2	-39.64	-40.13	-39.91	-40.05	-38.62	-38.54	-39.77	-39.03	-39.44	-37.82
bn3_bn3	3	-60.68	-61.61	-61.25	-61.02	-60.13	-59.43	-61.50	-60.04	-60.76	-58.71
bn4_bn4	4	-81.71	-83.04	-82.56	-82.50	-80.87	-80.11	-82.96	-80.89	-81.85	-78.97
bn5_bn5	5			-103.90	-103.70	-101.58	-101.06	-104.55			
bn6_bn6	6			-125.31	-124.55	-122.78	-121.64	-125.92			

Table S44: CCSD correlation part of the interaction energies for different BN_BN dimers for the aug-cc-pVTZ basis set in kJ/mol (cp-corrected).

aTZ		CCSD cor	LNO-CCSD cor				DLPNO-CCSD Tight	
System	Rings	canonical	vvTight	vTight	Tight	Normal	TcutPNO=1e-6	TcutPNO=1e-7
bn1_bn1	1	-21.56	-21.77	-21.72	-21.41	-19.96	-20.59	-21.14
bn2_bn2	2	-43.42	-43.83	-43.65	-42.92	-39.95	-42.36	-43.44
bn3_bn3	3		-66.50	-66.04	-64.72	-60.87	-64.65	-66.51
bn4_bn4	4			-88.37	-86.98	-81.95		-89.48
bn5_bn5	5			-110.80	-108.89	-102.42		-112.74
bn6_bn6	6			-133.38	-130.86	-123.37		

Table S45: CCSD correlation part of the interaction energies for different BN_BN dimers for the cc-pVQZ basis set in kJ/mol (cp-corrected).

QZ		CCSD cor	LNO-CCSD cor				DLPNO-CCSD Tight	
System	Rings	canonical	vvTight	vTight	Tight	Normal	TcutPNO=1e-6	TcutPNO=1e-7
bn1_bn1	1	-21.01	-21.60	-21.14	-21.57	-20.45	-20.02	-20.57
bn2_bn2	2	-42.60	-43.05	-42.85	-42.70	-40.66	-41.35	-42.50
bn3_bn3	3		-65.45	-65.14	-64.70	-61.51	-63.25	-65.12
bn4_bn4	4			-87.44	-86.58	-82.82	-85.20	
bn5_bn5	5			-109.69	-108.72	-103.77		
bn6_bn6	6			-132.34	-131.05	-125.10		

Table S46: CCSD correlation part of the interaction energies for different BN_BN dimers for the aug-cc-pVQZ basis set in kJ/mol (cp-corrected).

aQZ		CCSD cor	LNO-CCSD cor				DLPNO-CCSD Tight	
System	Rings	canonical	vvTight	vTight	Tight	Normal	TcutPNO=1e-6	TcutPNO=1e-7
bn1_bn1	1	-21.88	-22.16	-22.04	-21.73	-20.50	-21.10	-21.67
bn2_bn2	2			-44.28	-43.63	-41.21	-43.04	-44.03
bn3_bn3	3			-67.07	-65.99	-62.09		
bn4_bn4	4			-89.72	-88.54	-83.84		
bn5_bn5	5				-110.96	-105.04		
bn6_bn6	6				-133.28	-126.14		

Table S47: CCSD correlation part of the interaction energies for different BN_BN dimers for the cc-pV5Z basis set in kJ/mol (cp-corrected).

5Z		CCSD cor	LNO-CCSD cor			
System	Rings	canonical	vvTight	vTight	Tight	Normal
bn1_bn1	1	-21.68	-21.98	-21.89	-21.55	-20.32
bn2_bn2	2		-44.16	-43.98	-43.39	-40.74
bn3_bn3	3			-66.55	-65.69	-62.17
bn4_bn4	4			-89.27	-88.04	-83.45
bn5_bn5	5				-110.29	-104.56
bn6_bn6	6				-132.80	-126.04

Table S48: CCSD correlation part of the interaction energies for different BN_BN dimers for the aug-cc-pV5Z basis set in kJ/mol (cp-corrected).

a5Z		CCSD cor	LNO-CCSD cor			
System	Rings	canonical	vvTight	vTight	Tight	Normal
bn1_bn1	1		-22.25	-22.18	-21.83	-20.70
bn2_bn2	2			-44.44	-43.70	-41.38
bn3_bn3	3			-67.29	-66.34	-62.93
bn4_bn4	4			-90.14	-88.81	-84.20
bn5_bn5	5				-111.17	-105.89
bn6_bn6	6				-133.91	-126.68

Table S49: CCSD(T) correlation part of the interaction energies for different acene parallel displaced dimers for the cc-pVDZ basis set in kJ/mol(cp-uncorrected).

DZ		CCSD(T) cor	LNO-CCSD(T) cor				DLPNO-CCSD(T_1) Tight		PNO-LCCSD(T)		
System	Rings	canonical	vvTight	vTight	Tight	Normal	TcutPNO=1e-6	TcutPNO=1e-7	DOMOPT Tight	DOMOPT vTight	PAIROPT Tight
c_c	1	-21.65	-21.85	-21.90	-22.71	-24.73	-21.06	-21.37	-21.02	-21.22	-19.93
c2_c2	2	-47.30	-47.86	-48.07	-49.39	-53.22	-45.54	-47.11	-45.24	-45.81	-41.67
c3_c3	3	-73.88	-74.70	-75.03	-77.18	-83.12	-71.85	-74.06	-70.10	-71.11	-63.84
c4_c4	4	-101.06	-101.92	-102.43	-106.04	-115.06	-97.46	-100.99	-95.21	-96.49	-86.44
c5_c5	5	-128.78	-129.90	-130.48	-134.57	-145.31	-124.12	-128.42	-119.72	-121.54	-108.47
c6_c6	6	-156.20	-157.54	-158.25	-163.18	-176.58	-150.73	-155.84	-144.73	-146.76	-131.58

Table S50: CCSD(T) correlation part of the interaction energies for different acene parallel displaced dimers for the aug-cc-pVDZ basis set in kJ/mol(cp-uncorrected).

aDZ		CCSD(T) cor	LNO-CCSD(T) cor				PNO-LCCSD(T)		
System	Rings	canonical	vvTight	vTight	Tight	Normal	DOMOPT Tight	DOMOPT vTight	PAIROPT Tight
c_c	1	-35.30	-35.41	-35.43	-35.74	-37.20	-33.23	-33.49	-28.48
c2_c2	2	-75.86	-76.12	-76.30	-77.22	-80.79	-68.82	-69.38	-57.35
c3_c3	3	-118.05	-118.61	-118.95	-120.76	-126.31	-105.17	-106.14	-87.00
c4_c4	4	-160.75	-161.57	-161.96	-164.53	-172.11	-142.16	-143.42	-117.21
c5_c5	5			-206.47	-209.76	-219.79			
c6_c6	6			-250.34	-253.88	-267.26			

Table S51: CCSD(T) correlation part of the interaction energies for different acene parallel displaced dimers for the cc-pVTZ basis set in kJ/mol(cp-uncorrected).

TZ		CCSD(T) cor	LNO-CCSD(T) cor				DLPNO-CCSD(T_1) Tight		PNO-LCCSD(T)		
System	Rings	canonical	vvTight	vTight	Tight	Normal	TcutPNO=1e-6	TcutPNO=1e-7	DOMOPT Tight	DOMOPT vTight	PAIROPT Tight
c_c	1	-26.78	-26.92	-26.96	-27.47	-29.12	-29.56	-29.75	-25.80	-26.00	-24.38
c2_c2	2	-57.31	-57.68	-57.86	-58.99	-62.83	-55.14	-56.31	-54.39	-54.89	-50.29
c3_c3	3	-88.89	-89.45	-89.85	-91.72	-97.89	-85.56	-87.99	-83.52	-84.31	-77.50
c4_c4	4	-120.95	-121.73	-122.34	-124.76	-133.16	-116.06	-119.60	-113.43	-114.46	-104.85
c5_c5	5			-155.52	-159.12	-170.48		-151.91			
c6_c6	6			-188.51	-192.61	-206.65		-183.80			

Table S52: CCSD(T) correlation part of the interaction energies for different acene parallel displaced dimers for the aug-cc-pVTZ basis set in kJ/mol(cp-uncorrected).

aTZ		CCSD(T) cor	LNO-CCSD(T) cor			
System	Rings	canonical	vvTight	vTight	Tight	Normal
c_c	1	-31.53	-31.66	-31.75	-32.01	-33.48
c2_c2	2	-66.30	-66.64	-66.88	-67.60	-71.50
c3_c3	3			-103.42	-104.97	-110.50
c4_c4	4			-140.41	-142.36	-150.50
c5_c5	5			-178.09	-181.05	-191.21
c6_c6	6			-215.50	-218.38	-231.94

Table S53: CCSD(T) correlation part of the interaction energies for different acene parallel displaced dimers for the cc-pVQZ basis set in kJ/mol(cp-uncorrected).

QZ		CCSD(T) cor	LNO-CCSD(T) cor				DLPNO-CCSD(T_1) Tight	
System	Rings	canonical	vvTight	vTight	Tight	Normal	TcutPNO=1e-6	TcutPNO=1e-7
c_c	1	-27.28	-27.42	-27.50	-27.99	-29.82	-26.41	-26.96
c2_c2	2	-57.34	-57.71	-57.99	-59.22	-63.35	-55.42	
c3_c3	3			-89.46	-91.31	-97.14		
c4_c4	4			-121.41	-123.87	-132.45		
c5_c5	5			-154.37	-157.52	-168.90		
c6_c6	6			-186.76	-190.60	-204.84		

Table S54: CCSD(T) correlation part of the interaction energies for different acene parallel displaced dimers for the aug-cc-pVQZ basis set in kJ/mol(cp-uncorrected).

aQZ		CCSD(T) cor	LNO-CCSD(T) cor			
System	Rings	canonical	vvTight	vTight	Tight	Normal
c_c	1	-28.70	-28.85	-28.95	-29.40	-31.04
c2_c2	2		-60.04	-60.32	-61.19	-64.89
c3_c3	3			-92.45	-94.14	-100.06
c4_c4	4			-125.11	-127.62	-136.61
c5_c5	5				-162.02	-173.23
c6_c6	6				-195.79	-209.95

Table S55: CCSD(T) correlation part of the interaction energies for different acene parallel displaced dimers for the cc-pV5Z basis set in kJ/mol(cp-uncorrected).

5Z		CCSD(T) cor	LNO-CCSD(T) cor			
System	Rings	canonical	vvTight	vTight	Tight	Normal
c_c	1	-27.56	-27.69	-27.76	-28.26	-30.00
c2_c2	2		-57.81	-58.13	-59.32	-63.12
c3_c3	3			-89.34	-91.37	-97.29
c4_c4	4			-121.17	-124.52	-132.67
c5_c5	5				-157.31	-168.24
c6_c6	6				-190.44	-204.08

Table S56: CCSD(T) correlation part of the interaction energies for different acene parallel displaced dimers for the aug-cc-pV5Z basis set in kJ/mol(cp-uncorrected).

a5Z		CCSD(T) cor	LNO-CCSD(T) cor			
System	Rings	canonical	vvTight	vTight	Tight	Normal
c_c	1	-27.91	-28.02	-28.10	-28.46	-29.99
c2_c2	2		-58.23	-58.57	-59.37	-62.45
c3_c3	3			-89.85	-91.40	-97.00
c4_c4	4			-121.71	-123.77	-132.55
c5_c5	5				-157.89	-169.77
c6_c6	6				-190.45	-205.07

Table S57: CCSD correlation part of the interaction energies for different acene parallel displaced dimers for the cc-pVDZ basis set in kJ/mol(cp-uncorrected).

DZ		CCSD cor	LNO-CCSD cor				DLPNO-CCSD Tight		PNO-LCCSD		
System	Rings	canonical	vvTight	vTight	Tight	Normal	TcutPNO=1e-6	TcutPNO=1e-7	DOMOPT Tight	DOMOPT vTight	PAIROPT Tight
c_c	1	-18.19	-18.47	-18.50	-18.67	-18.06	-18.48	-18.35	-17.85	-17.96	-17.10
c2_c2	2	-39.35	-40.10	-40.19	-40.09	-39.27	-39.97	-40.35	-38.20	-38.48	-35.41
c3_c3	3	-61.14	-62.24	-62.32	-62.48	-61.18	-63.29	-63.57	-59.12	-59.59	-54.19
c4_c4	4	-83.38	-84.64	-84.74	-85.40	-84.83	-86.15	-86.84	-80.21	-80.78	-73.35
c5_c5	5	-106.02	-107.61	-107.63	-108.12	-107.14	-110.13	-110.92	-101.18	-101.84	-92.27
c6_c6	6	-128.36	-130.26	-130.27	-130.88	-129.95	-134.00	-134.76	-122.23	-123.02	-111.98

Table S58: CCSD correlation part of the interaction energies for different acene parallel displaced dimers for the aug-cc-pVDZ basis set in kJ/mol(cp-uncorrected).

aDZ		CCSD cor	LNO-CCSD cor				PNO-LCCSD		
System	Rings	canonical	vvTight	vTight	Tight	Normal	DOMOPT Tight	DOMOPT vTight	PAIROPT Tight
c_c	1	-29.52	-29.88	-29.92	-29.84	-29.51	-28.00	-28.12	-24.14
c2_c2	2	-63.00	-63.74	-63.90	-64.03	-63.64	-57.57	-57.79	-48.19
c3_c3	3	-97.66	-98.88	-99.11	-99.45	-98.95	-87.83	-88.20	-72.96
c4_c4	4	-132.66	-134.30	-134.47	-134.96	-134.25	-118.61	-119.08	-98.20
c5_c5	5			-171.09	-171.71	-170.93			
c6_c6	6			-207.07	-207.38	-207.12			

Table S59: CCSD correlation part of the interaction energies for different acene parallel displaced dimers for the cc-pVTZ basis set in kJ/mol(cp-uncorrected).

TZ		CCSD cor	LNO-CCSD cor				DLPNO-CCSD Tight		PNO-LCCSD		
System	Rings	canonical	vvTight	vTight	Tight	Normal	TcutPNO=1e-6	TcutPNO=1e-7	DOMOPT Tight	DOMOPT vTight	PAIROPT Tight
c_c	1	-22.10	-22.47	-22.50	-22.41	-22.00	-27.52	-27.35	-21.58	-21.62	-20.50
c2_c2	2	-46.95	-47.81	-47.93	-47.92	-47.29	-47.80	-47.51	-45.32	-45.41	-42.16
c3_c3	3	-72.52	-73.80	-74.02	-74.14	-73.44	-74.59	-74.42	-69.51	-69.65	-64.97
c4_c4	4	-98.43	-100.14	-100.41	-100.45	-99.79	-101.49	-101.51	-94.30	-94.47	-87.74
c5_c5	5			-127.36	-127.86	-127.34		-129.20			
c6_c6	6			-154.12	-154.45	-154.37		-156.71			

Table S60: CCSD correlation part of the interaction energies for different acene parallel displaced dimers for the aug-cc-pVTZ basis set in kJ/mol(cp-uncorrected).

aTZ		CCSD cor	LNO-CCSD cor			
System	Rings	canonical	vvTight	vTight	Tight	Normal
c_c	1	-26.28	-26.69	-26.80	-26.65	-26.18
c2_c2	2	-54.90	-55.79	-55.99	-55.83	-55.55
c3_c3	3			-86.12	-86.15	-85.42
c4_c4	4			-116.54	-116.31	-115.98
c5_c5	5			-147.43	-147.60	-147.00
c6_c6	6			-178.18	-177.75	-177.50

Table S61: CCSD correlation part of the interaction energies for different acene parallel displaced dimers for the cc-pVQZ basis set in kJ/mol(cp-uncorrected).

QZ		CCSD cor	LNO-CCSD cor				DLPNO-CCSD Tight	
System	Rings	canonical	vvTight	vTight	Tight	Normal	TcutPNO=1e-6	TcutPNO=1e-7
c_c	1	-22.33	-22.76	-22.81	-22.96	-22.56	-22.75	-22.65
c2_c2	2	-46.58	-47.50	-47.70	-47.88	-47.71	-47.95	
c3_c3	3			-73.12	-73.36	-72.94		
c4_c4	4			-98.88	-98.99	-98.98		
c5_c5	5			-125.08	-125.59	-125.73		
c6_c6	6			-151.10	-151.69	-152.09		

Table S62: CCSD correlation part of the interaction energies for different acene parallel displaced dimers for the aug-cc-pVQZ basis set in kJ/mol(cp-uncorrected).

aQZ		CCSD cor	LNO-CCSD cor			
System	Rings	canonical	vvTight	vTight	Tight	Normal
c_c	1	-23.57	-24.00	-24.09	-24.17	-23.73
c2_c2	2		-49.54	-49.72	-49.66	-49.13
c3_c3	3			-75.76	-75.79	-75.16
c4_c4	4			-102.11	-102.31	-102.30
c5_c5	5				-129.57	-129.42
c6_c6	6				-156.22	-156.17

Table S63: CCSD correlation part of the interaction energies for different acene parallel displaced dimers for the cc-pV5Z basis set in kJ/mol(cp-uncorrected).

5Z		CCSD cor	LNO-CCSD cor			
System	Rings	canonical	vvTight	vTight	Tight	Normal
c_c	1	-22.53	-22.95	-22.99	-22.99	-22.55
c2_c2	2		-47.49	-47.66	-47.78	-47.38
c3_c3	3			-72.87	-73.07	-72.78
c4_c4	4			-98.43	-99.11	-98.70
c5_c5	5				-124.86	-124.91
c6_c6	6				-150.82	-150.99

Table S64: CCSD correlation part of the interaction energies for different acene parallel displaced dimers for the aug-cc-pV5Z basis set in kJ/mol(cp-uncorrected).

a5Z		CCSD cor	LNO-CCSD cor			
System	Rings	canonical	vvTight	vTight	Tight	Normal
c_c	1	-22.83	-23.23	-23.31	-23.27	-22.90
c2_c2	2		-47.84	-48.04	-47.98	-47.22
c3_c3	3			-73.25	-73.26	-72.76
c4_c4	4			-98.85	-98.62	-98.95
c5_c5	5				-125.57	-126.30
c6_c6	6				-151.14	-151.86

Table S65: CCSD(T) correlation part of the interaction energies for different BNx_NBx dimers for the cc-pVDZ basis set in kJ/mol(cp-uncorrected).

DZ		CCSD(T) cor	LNO-CCSD(T) cor				DLPNO-CCSD(T_1) Tight		PNO-LCCSD(T)		
System	Rings	canonical	vvTight	vTight	Tight	Normal	TcutPNO=1e-6	TcutPNO=1e-7	DOMOPT Tight	DOMOPT vTight	PAIROPT Tight
bn1_nb1	1	-18.34	-18.14	-18.06	-17.79	-18.61	-17.06	-17.46	-17.09	-17.44	-16.08
bn2_nb2	2	-37.54	-37.10	-37.01	-37.36	-38.30	-34.87	-36.13	-34.39	-35.05	-31.27
bn3_nb3	3	-57.77	-57.26	-57.15	-57.17	-59.12	-53.10	-55.24	-51.91	-52.95	-46.52
bn4_nb4	4	-78.47	-77.89	-77.81	-78.15	-80.00	-71.92	-74.65	-69.70	-71.19	-62.24
bn5_nb5	5	-95.00	-94.35	-94.33	-95.01	-96.62	-87.17	-90.01	-82.79	-84.66	-74.32
bn6_nb6	6	-116.24	-115.41	-115.40	-116.43	-118.70	-106.17	-109.65	-100.90	-103.17	-90.36

Table S66: CCSD(T) correlation part of the interaction energies for different BNx_NBx dimers for the aug-cc-pVDZ basis set in kJ/mol(cp-uncorrected).

aDZ		CCSD(T) cor	LNO-CCSD(T) cor				DLPNO-CCSD(T_1) Tight		PNO-LCCSD(T)		
System	Rings	canonical	vvTight	vTight	Tight	Normal	TcutPNO=1e-6	TcutPNO=1e-7	DOMOPT Tight	DOMOPT vTight	PAIROPT Tight
bn1_nb1	1	-27.20	-27.10	-26.98	-27.26	-28.53	-25.42	-26.39	-22.64	-22.98	-20.04
bn2_nb2	2	-55.41	-54.92	-54.77	-55.37	-57.01	-52.08	-53.84	-46.47	-47.36	-39.85
bn3_nb3	3	-85.10	-84.56	-84.33	-85.22	-88.05	-79.86	-82.56	-69.88	-71.26	-59.39
bn4_nb4	4		-115.01	-114.84	-116.34	-119.55	-107.93	-111.57	-93.31	-95.17	-79.55
bn5_nb5	5			-139.92	-141.79	-145.57	-131.59	-135.95			
bn6_nb6	6			-172.01	-174.79	-179.87	-160.30				

Table S67: CCSD(T) correlation part of the interaction energies for different BNx_NBx dimers for the cc-pVTZ basis set in kJ/mol(cp-uncorrected).

TZ		CCSD(T) cor	LNO-CCSD(T) cor				DLPNO-CCSD(T_1) Tight		PNO-LCCSD(T)		
System	Rings	canonical	vvTight	vTight	Tight	Normal	TcutPNO=1e-6	TcutPNO=1e-7	DOMOPT Tight	DOMOPT vTight	PAIROPT Tight
bn1_nb1	1	-19.31	-19.21	-19.03	-19.49	-20.36	-17.56	-18.30	-17.71	-18.04	-16.91
bn2_nb2	2	-38.16	-37.95	-37.80	-38.49	-39.96	-35.14	-36.66	-35.19	-35.89	-32.50
bn3_nb3	3	-57.84	-57.47	-57.31	-58.42	-60.71	-52.81	-55.40	-51.95	-53.26	-47.39
bn4_nb4	4	-77.99	-77.44	-77.33	-79.03	-81.88	-71.55	-75.01	-69.51	-71.23	-63.69
bn5_nb5	5			-93.10	-95.09	-98.34	-85.99				
bn6_nb6	6			-113.65	-115.92	-120.09	-105.01				

Table S68: CCSD(T) correlation part of the interaction energies for different BNx_NBx dimers for the aug-cc-pVTZ basis set in kJ/mol(cp-uncorrected).

aTZ		CCSD(T) cor	LNO-CCSD(T) cor				DLPNO-CCSD(T_1) Tight	
System	Rings	canonical	vvTight	vTight	Tight	Normal	TcutPNO=1e-6	TcutPNO=1e-7
bn1_nb1	1	-23.02	-22.85	-22.74	-22.92	-23.81	-21.62	-22.47
bn2_nb2	2	-44.74	-44.45	-44.31	-45.04	-46.90	-41.86	-43.49
bn3_nb3	3		-67.48	-67.26	-67.95	-70.34	-63.68	-66.07
bn4_nb4	4			-90.48	-91.71	-95.14		-88.59
bn5_nb5	5			-109.20	-110.82	-114.97		-107.25
bn6_nb6	6			-133.23	-135.34	-140.61		

Table S69: CCSD(T) correlation part of the interaction energies for different BNx_NBx dimers for the cc-pVQZ basis set in kJ/mol(cp-uncorrected).

QZ		CCSD(T) cor	LNO-CCSD(T) cor				DLPNO-CCSD(T_1) Tight	
System	Rings	canonical	vvTight	vTight	Tight	Normal	TcutPNO=1e-6	TcutPNO=1e-7
bn1_nb1	1	-19.60	-19.49	-19.34	-19.75	-20.74	-18.22	-18.85
bn2_nb2	2	-37.75	-37.47	-37.37	-38.02	-39.33	-35.12	-36.52
bn3_nb3	3		-56.25	-56.18	-57.24	-59.44	-52.53	-54.82
bn4_nb4	4			-75.42	-76.88	-79.85	-70.39	
bn5_nb5	5			-90.47	-92.31	-96.01		
bn6_nb6	6			-110.90	-112.60	-116.91		

Table S70: CCSD(T) correlation part of the interaction energies for different BNx_NBx dimers for the aug-cc-pVQZ basis set in kJ/mol(cp-uncorrected).

aQZ		CCSD(T) cor	LNO-CCSD(T) cor				DLPNO-CCSD(T_1) Tight	
System	Rings	canonical	vvTight	vTight	Tight	Normal	TcutPNO=1e-6	TcutPNO=1e-7
bn1_nb1	1	-20.47	-19.60	-20.44	-20.54	-21.62	-19.23	-19.96
bn2_nb2	2			-39.18	-39.54	-41.68	-36.90	-38.26
bn3_nb3	3			-58.59	-59.17	-61.72		
bn4_nb4	4			-78.25	-79.04	-82.37		
bn5_nb5	5				-94.90	-99.45		
bn6_nb6	6				-115.84	-120.73		

Table S71: CCSD(T) correlation part of the interaction energies for different BNx_NBx dimers for the cc-pV5Z basis set in kJ/mol(cp-uncorrected).

5Z		CCSD(T) cor	LNO-CCSD(T) cor				DLPNO-CCSD(T_1) Tight	
System	Rings	canonical	vvTight	vTight	Tight	Normal	TcutPNO=1e-6	TcutPNO=1e-7
bn1_nb1	1	-19.60	-19.47	-19.35	-19.74	-20.56	-18.34	-18.93
bn2_nb2	2		-37.42	-37.12	-37.79	-39.69		
bn3_nb3	3			-55.57	-56.54	-59.44		
bn4_nb4	4			-74.43	-75.93	-79.84		
bn5_nb5	5				-91.02	-96.19		
bn6_nb6	6				-110.99	-116.83		

Table S72: CCSD(T) correlation part of the interaction energies for different BNx_NBx dimers for the aug-cc-pV5Z basis set in kJ/mol(cp-uncorrected).

a5Z		CCSD(T) cor	LNO-CCSD(T) cor				DLPNO-CCSD(T_1) Tight	
System	Rings	canonical	vvTight	vTight	Tight	Normal	TcutPNO=1e-6	TcutPNO=1e-7
bn1_nb1	1		-19.71	-19.62	-19.84	-20.69	-18.71	-19.28
bn2_nb2	2			-37.44	-38.19	-39.46		
bn3_nb3	3			-55.80	-56.95	-59.74		
bn4_nb4	4			-74.72	-75.88	-78.74		
bn5_nb5	5				-91.33	-95.36		
bn6_nb6	6				-111.01	-116.15		

Table S73: CCSD correlation part of the interaction energies for different BNx_NBx dimers for the cc-pVDZ basis set in kJ/mol(cp-uncorrected).

DZ		CCSD cor	LNO-CCSD cor				DLPNO-CCSD Tight		PNO-LCCSD		
System	Rings	canonical	vvTight	vTight	Tight	Normal	TcutPNO=1e-6	TcutPNO=1e-7	DOMOPT Tight	DOMOPT vTight	PAIROPT Tight
bn1_nb1	1	-15.59	-15.59	-15.48	-14.52	-13.49	-15.16	-15.21	-14.68	-14.91	-13.85
bn2_nb2	2	-31.76	-31.75	-31.49	-30.22	-27.79	-30.94	-31.57	-29.38	-29.83	-26.83
bn3_nb3	3	-48.78	-48.94	-48.51	-46.31	-42.50	-47.17	-48.28	-44.38	-45.04	-39.91
bn4_nb4	4	-66.20	-66.46	-65.90	-63.21	-57.44	-63.86	-65.27	-59.62	-60.55	-53.41
bn5_nb5	5	-80.14	-80.48	-79.74	-76.66	-68.96	-77.61	-78.89	-70.91	-72.05	-63.83
bn6_nb6	6	-98.01	-98.35	-97.48	-93.95	-85.15	-94.44	-96.05	-86.44	-87.82	-77.61

Table S74: CCSD correlation part of the interaction energies for different BNx_NBx dimers for the aug-cc-pVDZ basis set in kJ/mol(cp-uncorrected).

aDZ		CCSD cor	LNO-CCSD cor				DLPNO-CCSD Tight		PNO-LCCSD		
System	Rings	canonical	vvTight	vTight	Tight	Normal	TcutPNO=1e-6	TcutPNO=1e-7	DOMOPT Tight	DOMOPT vTight	PAIROPT Tight
bn1_nb1	1	-22.96	-23.20	-23.15	-22.96	-21.77	-22.07	-22.64	-19.14	-19.35	-16.94
bn2_nb2	2	-46.62	-46.84	-46.67	-46.09	-43.79	-45.44	-46.35	-39.18	-39.79	-33.53
bn3_nb3	3	-71.57	-72.02	-71.63	-70.55	-67.55	-69.82	-71.22	-58.91	-59.85	-50.00
bn4_nb4	4		-97.90	-97.48	-96.20	-91.41	-94.49	-96.33	-78.65	-79.91	-67.01
bn5_nb5	5			-118.59	-116.92	-111.13	-115.44	-117.63			
bn6_nb6	6			-145.48	-144.50	-139.22	-140.67				

Table S75: CCSD correlation part of the interaction energies for different BNx_NBx dimers for the cc-pVTZ basis set in kJ/mol(cp-uncorrected).

TZ		CCSD cor	LNO-CCSD cor				DLPNO-CCSD Tight		PNO-LCCSD		
System	Rings	canonical	vvTight	vTight	Tight	Normal	TcutPNO=1e-6	TcutPNO=1e-7	DOMOPT Tight	DOMOPT vTight	PAIROPT Tight
bn1_nb1	1	-15.99	-16.21	-16.08	-15.94	-14.31	-15.24	-15.57	-14.81	-14.99	-14.19
bn2_nb2	2	-31.54	-31.96	-31.71	-31.00	-28.18	-30.58	-31.36	-29.37	-29.82	-27.20
bn3_nb3	3	-47.77	-48.35	-47.96	-46.94	-43.08	-46.10	-47.50	-43.60	-44.29	-39.92
bn4_nb4	4	-64.39	-65.10	-64.58	-63.25	-58.42	-62.58	-64.46	-58.32	-59.23	-53.65
bn5_nb5	5			-77.62	-75.77	-69.63	-75.45				
bn6_nb6	6			-94.69	-92.27	-85.12	-92.17				

Table S76: CCSD correlation part of the interaction energies for different BNx_NBx dimers for the aug-cc-pVTZ basis set in kJ/mol(cp-uncorrected).

aTZ		CCSD cor	LNO-CCSD cor				DLPNO-CCSD Tight	
System	Rings	canonical	vvTight	vTight	Tight	Normal	TcutPNO=1e-6	TcutPNO=1e-7
bn1_nb1	1	-19.24	-19.47	-19.40	-19.13	-17.49	-18.83	-19.28
bn2_nb2	2	-37.42	-37.89	-37.69	-37.23	-34.85	-36.70	-37.45
bn3_nb3	3		-57.51	-57.09	-55.82	-51.94	-56.08	-57.12
bn4_nb4	4			-76.66	-75.08	-70.41		-76.64
bn5_nb5	5			-92.46	-90.43	-84.85		-93.01
bn6_nb6	6			-112.76	-110.45	-104.01		

Table S77: CCSD correlation part of the interaction energies for different BNx_NBx dimers for the cc-pVQZ basis set in kJ/mol(cp-uncorrected).

QZ		CCSD cor	LNO-CCSD cor				DLPNO-CCSD Tight	
System	Rings	canonical	vvTight	vTight	Tight	Normal	TcutPNO=1e-6	TcutPNO=1e-7
bn1_nb1	1	-16.06	-16.31	-16.22	-16.03	-15.18	-15.62	-15.87
bn2_nb2	2	-30.88	-31.36	-31.19	-30.62	-27.83	-30.39	-30.96
bn3_nb3	3		-46.98	-46.70	-45.68	-42.00	-45.62	-46.57
bn4_nb4	4			-62.53	-61.09	-56.27	-61.22	
bn5_nb5	5			-74.88	-72.91	-67.08		
bn6_nb6	6			-91.40	-88.94	-81.77		

Table S78: CCSD correlation part of the interaction energies for different BNx_NBx dimers for the aug-cc-pVQZ basis set in kJ/mol(cp-uncorrected).

aQZ		CCSD cor	LNO-CCSD cor				DLPNO-CCSD Tight	
System	Rings	canonical	vvTight	vTight	Tight	Normal	TcutPNO=1e-6	TcutPNO=1e-7
bn1_nb1	1	-16.81	-16.62	-17.08	-16.75	-15.59	-16.55	-16.89
bn2_nb2	2			-32.61	-31.89	-29.77	-31.99	-32.50
bn3_nb3	3			-48.60	-47.35	-43.78		
bn4_nb4	4			-64.75	-63.15	-58.49		
bn5_nb5	5				-75.34	-69.82		
bn6_nb6	6				-91.98	-85.10		

Table S79: CCSD correlation part of the interaction energies for different BNx_NBx dimers for the cc-pV5Z basis set in kJ/mol(cp-uncorrected).

5Z		CCSD cor	LNO-CCSD cor				DLPNO-CCSD Tight	
System	Rings	canonical	vvTight	vTight	Tight	Normal	TcutPNO=1e-6	TcutPNO=1e-7
bn1_nb1	1	-16.02	-16.27	-16.21	-15.95	-14.77	-15.74	-15.91
bn2_nb2	2		-31.02	-30.87	-30.30	-27.85		
bn3_nb3	3			-46.01	-44.89	-41.59		
bn4_nb4	4			-61.51	-60.18	-55.66		
bn5_nb5	5				-71.90	-66.77		
bn6_nb6	6				-87.51	-81.08		

Table S80: CCSD correlation part of the interaction energies for different BNx_NBx dimers for the aug-cc-pV5Z basis set in kJ/mol(cp-uncorrected).

a5Z		CCSD cor	LNO-CCSD cor				DLPNO-CCSD Tight	
System	Rings	canonical	vvTight	vTight	Tight	Normal	TcutPNO=1e-6	TcutPNO=1e-7
bn1_nb1	1		-16.45	-16.39	-16.05	-14.71	-16.11	-16.22
bn2_nb2	2			-31.10	-30.71	-28.23		
bn3_nb3	3			-46.14	-45.24	-42.23		
bn4_nb4	4			-61.66	-60.06	-55.23		
bn5_nb5	5				-72.05	-66.59		
bn6_nb6	6				-87.40	-81.26		

Table S81: CCSD(T) correlation part of the interaction energies for different BNx_BNx dimers for the cc-pVDZ basis set in kJ/mol(cp-uncorrected).

DZ		CCSD(T) cor	LNO-CCSD(T) cor				DLPNO-CCSD(T_1) Tight		PNO-LCCSD(T)		
System	Rings	canonical	vvTight	vTight	Tight	Normal	TcutPNO=1e-6	TcutPNO=1e-7	DOMOPT Tight	DOMOPT vTight	PAIROPT Tight
bn1_bn1	1	-25.82	-25.76	-25.55	-25.34	-25.49	-24.04	-24.49	-24.68	-25.11	-21.94
bn2_bn2	2	-56.72	-56.29	-55.98	-56.07	-57.29	-53.16	-54.78	-52.53	-54.05	-42.46
bn3_bn3	3	-89.11	-88.44	-88.00	-87.95	-90.09	-82.90	-86.29	-82.17	-84.19	-67.01
bn4_bn4	4	-121.54	-120.59	-120.11	-120.22	-122.59	-112.65	-117.36	-111.79	-114.31	-91.41
bn5_bn5	5	-153.98	-152.93	-152.32	-152.38	-155.52	-141.80	-147.81	-141.24	-144.20	-115.96
bn6_bn6	6	-186.53	-185.23	-184.59	-184.88	-188.58	-171.28	-178.82	-170.82	-174.25	-140.49

Table S82: CCSD(T) correlation part of the interaction energies for different BNx_BNx dimers for the aug-cc-pVDZ basis set in kJ/mol(cp-uncorrected).

aDZ		CCSD(T) cor	LNO-CCSD(T) cor				DLPNO-CCSD(T_1) Tight		PNO-LCCSD(T)		
System	Rings	canonical	vvTight	vTight	Tight	Normal	TcutPNO=1e-6	TcutPNO=1e-7	DOMOPT Tight	DOMOPT vTight	PAIROPT Tight
bn1_bn1	1	-35.87	-35.62	-35.41	-35.09	-36.27	-33.33	-34.58	-33.59	-34.01	-25.59
bn2_bn2	2	-75.95	-75.38	-75.05	-74.92	-76.38	-70.99	-73.56	-67.59	-68.64	-50.42
bn3_bn3	3	-117.83	-117.03	-116.55	-117.03	-119.88	-110.66	-114.70	-102.66	-104.38	-78.27
bn4_bn4	4		-159.04	-158.47	-159.06	-163.22	-149.44	-155.07	-137.91	-140.14	-106.57
bn5_bn5	5			-200.56	-201.28	-205.75	-187.66	-194.67	-173.28	-176.01	-134.64
bn6_bn6	6			-242.52	-243.43	-248.99	-225.85	-235.09			

Table S83: CCSD(T) correlation part of the interaction energies for different BNx_BNx dimers for the cc-pVTZ basis set in kJ/mol(cp-uncorrected).

TZ		CCSD(T) cor	LNO-CCSD(T) cor				DLPNO-CCSD(T_1) Tight		PNO-LCCSD(T)		
System	Rings	canonical	vvTight	vTight	Tight	Normal	TcutPNO=1e-6	TcutPNO=1e-7	DOMOPT Tight	DOMOPT vTight	PAIROPT Tight
bn1_bn1	1	-27.72	-27.47	-27.21	-27.30	-28.41	-25.34	-26.47	-26.60	-26.97	-23.96
bn2_bn2	2	-57.24	-56.75	-56.47	-56.78	-58.66	-52.57	-54.87	-53.41	-54.54	-46.29
bn3_bn3	3	-87.82	-87.15	-86.77	-87.26	-90.24	-80.36	-84.40	-81.53	-83.17	-71.28
bn4_bn4	4	-118.36	-117.42	-117.00	-117.76	-121.53	-108.41	-113.74	-109.56	-111.62	-96.15
bn5_bn5	5			-147.26	-148.00	-152.65	-135.77	-142.62			
bn6_bn6	6			-177.64	-178.65	-184.29	-163.38	-171.65			

Table S84: CCSD(T) correlation part of the interaction energies for different BNx_BNx dimers for the aug-cc-pVTZ basis set in kJ/mol(cp-uncorrected).

aTZ		CCSD(T) cor	LNO-CCSD(T) cor				DLPNO-CCSD(T_1) Tight	
System	Rings	canonical	vvTight	vTight	Tight	Normal	TcutPNO=1e-6	TcutPNO=1e-7
bn1_bn1	1	-31.53	-31.28	-31.09	-31.04	-32.00	-29.25	-30.37
bn2_bn2	2	-64.68	-64.27	-64.00	-64.40	-65.81	-60.34	-62.76
bn3_bn3	3		-98.56	-98.05	-98.17	-100.93	-92.47	-96.26
bn4_bn4	4			-132.11	-132.53	-136.54		-129.40
bn5_bn5	5			-166.14	-166.56	-171.59		-162.44
bn6_bn6	6			-200.23	-200.56	-206.41		

Table S85: CCSD(T) correlation part of the interaction energies for different BNx_BNx dimers for the cc-pVQZ basis set in kJ/mol(cp-uncorrected).

QZ		CCSD(T) cor	LNO-CCSD(T) cor				DLPNO-CCSD(T_1) Tight	
System	Rings	canonical	vvTight	vTight	Tight	Normal	TcutPNO=1e-6	TcutPNO=1e-7
bn1_bn1	1	-27.28	-27.05	-26.83	-26.88	-28.32	-24.93	-26.06
bn2_bn2	2	-55.56	-55.06	-54.82	-55.17	-57.19	-51.18	-53.47
bn3_bn3	3		-84.04	-83.82	-84.19	-87.21	-77.94	-81.73
bn4_bn4	4			-112.58	-113.21	-117.22	-104.83	
bn5_bn5	5			-141.44	-142.22	-146.98		
bn6_bn6	6			-171.32	-171.34	-177.20		

Table S86: CCSD(T) correlation part of the interaction energies for different BNx_BNx dimers for the aug-cc-pVQZ basis set in kJ/mol(cp-uncorrected).

aQZ		CCSD(T) cor	LNO-CCSD(T) cor				DLPNO-CCSD(T_1) Tight	
System	Rings	canonical	vvTight	vTight	Tight	Normal	TcutPNO=1e-6	TcutPNO=1e-7
bn1_bn1	1	-28.25	-28.07	-27.86	-27.98	-29.43	-26.12	-27.31
bn2_bn2	2			-56.60	-57.14	-59.14	-53.43	-55.59
bn3_bn3	3			-85.88	-86.70	-89.90		
bn4_bn4	4			-115.02	-116.46	-120.10		
bn5_bn5	5				-145.82	-150.40		
bn6_bn6	6				-175.41	-181.70		

Table S87: CCSD(T) correlation part of the interaction energies for different BNx_BNx dimers for the cc-pV5Z basis set in kJ/mol(cp-uncorrected).

5Z		CCSD(T) cor	LNO-CCSD(T) cor			
System	Rings	canonical	vvTight	vTight	Tight	Normal
bn1_bn1	1	-27.15	-26.94	-26.73	-26.84	-27.82
bn2_bn2	2		-54.77	-54.24	-54.62	-56.38
bn3_bn3	3			-82.44	-83.09	-86.12
bn4_bn4	4			-110.76	-111.76	-115.79
bn5_bn5	5				-140.27	-145.52
bn6_bn6	6				-168.97	-175.38

Table S88: CCSD(T) correlation part of the interaction energies for different BNx_BNx dimers for the aug-cc-pV5Z basis set in kJ/mol(cp-uncorrected).

a5Z		CCSD(T) cor	LNO-CCSD(T) cor			
System	Rings	canonical	vvTight	vTight	Tight	Normal
bn1_bn1	1		-27.21	-27.05	-27.12	-27.97
bn2_bn2	2			-54.60	-55.09	-56.89
bn3_bn3	3			-82.97	-83.66	-86.53
bn4_bn4	4			-111.29	-112.11	-116.34
bn5_bn5	5				-140.66	-145.65
bn6_bn6	6				-169.37	-175.42

Table S89: CCSD correlation part of the interaction energies for different BNx_BNx dimers for the cc-pVDZ basis set in kJ/mol(cp-uncorrected).

DZ		CCSD cor	LNO-CCSD cor				DLPNO-CCSD Tight		PNO-LCCSD		
System	Rings	canonical	vvTight	vTight	Tight	Normal	TcutPNO=1e-6	TcutPNO=1e-7	DOMOPT Tight	DOMOPT vTight	PAIROPT Tight
bn1_bn1	1	-21.80	-21.99	-21.87	-21.04	-18.70	-21.16	-21.12	-21.06	-21.33	-18.82
bn2_bn2	2	-47.70	-47.78	-47.52	-46.36	-43.23	-46.68	-47.35	-45.28	-45.77	-36.96
bn3_bn3	3	-74.78	-74.95	-74.48	-72.70	-68.10	-72.99	-74.67	-70.42	-71.19	-57.86
bn4_bn4	4	-101.89	-102.05	-101.44	-99.27	-92.65	-99.15	-101.58	-95.56	-96.61	-78.64
bn5_bn5	5	-129.02	-129.32	-128.51	-125.65	-117.41	-124.84	-127.96	-120.56	-121.84	-99.56
bn6_bn6	6	-156.23	-156.54	-155.60	-152.46	-142.67	-150.77	-154.85	-145.67	-147.21	-120.46

Table S90: CCSD correlation part of the interaction energies for different BNx_BNx dimers for the aug-cc-pVDZ basis set in kJ/mol(cp-uncorrected).

aDZ		CCSD cor	LNO-CCSD cor				DLPNO-CCSD Tight		PNO-LCCSD		
System	Rings	canonical	vvTight	vTight	Tight	Normal	TcutPNO=1e-6	TcutPNO=1e-7	DOMOPT Tight	DOMOPT vTight	PAIROPT Tight
bn1_bn1	1	-30.29	-30.41	-30.37	-29.75	-28.77	-29.05	-29.66	-28.52	-28.77	-21.69
bn2_bn2	2	-63.91	-64.13	-63.96	-63.11	-60.47	-62.01	-63.21	-57.24	-57.82	-42.72
bn3_bn3	3	-99.06	-99.41	-99.09	-98.21	-94.57	-96.80	-98.70	-86.78	-87.81	-66.22
bn4_bn4	4		-134.99	-134.54	-133.09	-128.46	-130.79	-133.50	-116.49	-117.85	-90.13
bn5_bn5	5			-170.08	-168.37	-161.68	-164.39	-167.69	-146.32	-147.99	-113.81
bn6_bn6	6			-205.63	-203.49	-195.67	-197.92	-202.58			

Table S91: CCSD correlation part of the interaction energies for different BNx_BNx dimers for the cc-pVTZ basis set in kJ/mol(cp-uncorrected).

TZ		CCSD cor	LNO-CCSD cor				DLPNO-CCSD Tight		PNO-LCCSD		
System	Rings	canonical	vvTight	vTight	Tight	Normal	TcutPNO=1e-6	TcutPNO=1e-7	DOMOPT Tight	DOMOPT vTight	PAIROPT Tight
bn1_bn1	1	-22.99	-23.17	-23.06	-22.75	-21.43	-22.03	-22.49	-22.31	-22.49	-20.16
bn2_bn2	2	-47.35	-47.71	-47.47	-46.77	-44.16	-45.75	-46.71	-45.04	-45.43	-39.18
bn3_bn3	3	-72.56	-73.10	-72.70	-71.58	-67.72	-70.06	-71.92	-68.56	-69.21	-60.13
bn4_bn4	4	-97.71	-98.38	-97.86	-96.39	-91.02	-94.51	-96.90	-91.99	-92.87	-80.97
bn5_bn5	5			-122.99	-120.93	-114.18	-118.51	-121.62			
bn6_bn6	6			-148.26	-145.84	-138.02	-142.62	-146.35			

Table S92: CCSD correlation part of the interaction energies for different BNx_BNx dimers for the aug-cc-pVTZ basis set in kJ/mol(cp-uncorrected).

aTZ		CCSD cor	LNO-CCSD cor				DLPNO-CCSD Tight	
System	Rings	canonical	vvTight	vTight	Tight	Normal	TcutPNO=1e-6	TcutPNO=1e-7
bn1_bn1	1	-26.45	-26.66	-26.58	-26.26	-24.75	-25.60	-26.02
bn2_bn2	2	-54.25	-54.73	-54.56	-54.08	-51.06	-53.12	-54.05
bn3_bn3	3		-83.83	-83.38	-82.09	-77.97	-81.59	-82.98
bn4_bn4	4			-112.16	-110.51	-105.28		-111.59
bn5_bn5	5			-140.88	-138.65	-132.09		-140.22
bn6_bn6	6			-169.62	-166.75	-158.85		

Table S93: CCSD correlation part of the interaction energies for different BNx_BNx dimers for the cc-pVQZ basis set in kJ/mol(cp-uncorrected).

QZ		CCSD cor	LNO-CCSD cor				DLPNO-CCSD Tight	
System	Rings	canonical	vvTight	vTight	Tight	Normal	TcutPNO=1e-6	TcutPNO=1e-7
bn1_bn1	1	-22.45	-22.67	-22.59	-22.06	-21.43	-21.50	-21.98
bn2_bn2	2	-45.61	-46.03	-45.82	-45.29	-42.61	-44.41	-45.24
bn3_bn3	3		-70.06	-69.77	-68.62	-64.84	-67.75	-69.27
bn4_bn4	4			-93.55	-92.06	-86.94	-91.20	
bn5_bn5	5			-117.37	-115.39	-108.80		
bn6_bn6	6			-141.46	-138.96	-131.03		

Table S94: CCSD correlation part of the interaction energies for different BNx_BNx dimers for the aug-cc-pVQZ basis set in kJ/mol(cp-uncorrected).

aQZ		CCSD cor	LNO-CCSD cor				DLPNO-CCSD Tight	
System	Rings	canonical	vvTight	vTight	Tight	Normal	TcutPNO=1e-6	TcutPNO=1e-7
bn1_bn1	1	-23.32	-23.58	-23.49	-23.17	-22.30	-22.69	-23.13
bn2_bn2	2			-47.43	-46.94	-44.52	-46.54	-47.25
bn3_bn3	3			-71.69	-70.89	-67.24		
bn4_bn4	4			-95.80	-94.98	-89.84		
bn5_bn5	5				-118.68	-112.14		
bn6_bn6	6				-142.62	-135.31		

Table S95: CCSD correlation part of the interaction energies for different BNx_BNx dimers for the cc-pV5Z basis set in kJ/mol(cp-uncorrected).

5Z		CCSD cor	LNO-CCSD cor			
System	Rings	canonical	vvTight	vTight	Tight	Normal
bn1_bn1	1	-22.30	-22.55	-22.45	-22.11	-20.91
bn2_bn2	2		-45.43	-45.20	-44.59	-41.76
bn3_bn3	3			-68.52	-67.47	-63.53
bn4_bn4	4			-91.83	-90.52	-85.34
bn5_bn5	5				-113.43	-107.22
bn6_bn6	6				-136.45	-129.12

Table S96: CCSD correlation part of the interaction energies for different BNx_BNx dimers for the aug-cc-pV5Z basis set in kJ/mol(cp-uncorrected).

a5Z		CCSD cor	LNO-CCSD cor			
System	Rings	canonical	vvTight	vTight	Tight	Normal
bn1_bn1	1		-22.76	-22.69	-22.33	-21.24
bn2_bn2	2			-45.50	-45.01	-42.48
bn3_bn3	3			-68.88	-68.00	-64.36
bn4_bn4	4			-92.19	-90.86	-85.93
bn5_bn5	5				-113.72	-107.67
bn6_bn6	6				-136.77	-129.33

Table S97: Correlation part of the interaction energies for different Acene PD dimers and (extrapolated) basis sets (cp-corrected) in kJ/mol using LNO-CCSD(T) with Tight criteria.

	DZ	aDZ	TZ	{D,T}Z	aTZ	a{D,T}Z	QZ	{T,Q}Z	aQZ	a{T,Q}Z	5Z	{Q,5}Z	a5Z	a{Q,5}Z
1	-20.03	-26.35	-26.03	-28.56	-27.45	-27.91	-26.83	-27.42	-27.83	-28.12	-27.76	-28.74	-27.99	-28.15
2	-41.21	-55.44	-53.00	-57.96	-57.33	-58.13	-56.64	-59.29	-58.09	-58.64	-58.38	-60.22	-58.42	-58.76
3	-64.44	-85.76	-82.17	-89.63	-88.53	-89.69	-87.37	-91.17	-89.36	-89.98	-89.64	-92.02	-89.67	-89.99
4	-87.93	-116.30	-112.03	-122.18	-120.32	-122.02	-118.62	-123.42	-121.28	-121.99	-121.66	-124.85	-121.79	-122.31
5	-111.24	-147.95	-142.58	-155.77	-153.14	-155.32	-150.69	-156.62	-153.98	-154.59	-154.14	-157.76	-154.27	-154.57
6	-134.89	-178.51	-172.79	-188.75	-185.16	-187.96	-182.36	-189.35	-186.29	-187.11	-186.47	-190.77	-186.72	-187.16

Table S98: Correlation part of the interaction energies for different Acene$_x$ PD dimers and (extrapolated) basis sets (cp-corrected) in kJ/mol using LNO-CCSD(T) with very Tight criteria.

	DZ	aDZ	TZ	{D,T}Z	aTZ	a{D,T}Z	QZ	{T,Q}Z	aQZ	a{T,Q}Z	5Z	{Q,5}Z	a5Z	a{Q,5}Z
1	-18.42	-25.99	-24.22	-26.66	-27.12	-27.60	-26.28	-27.79	-27.56	-27.87	-27.15	-28.07	-27.61	-27.66
2	-39.54	-54.54	-51.30	-56.25	-56.64	-57.52	-55.15	-57.97	-57.15	-57.53	-56.77	-58.48	-57.41	-57.68
3	-61.55	-84.19	-79.45	-86.99	-87.11	-88.35	-85.01	-89.07	-87.67	-88.08	-87.32	-89.74	-88.05	-88.45
4	-83.91	-113.85	-107.98	-118.12	-117.99	-119.73	-115.38	-120.79	-118.79	-119.38	-118.38	-121.51	-119.39	-120.01
5	-106.51	-144.53	-136.75	-149.48	-149.99	-152.29	-146.70	-153.96						
6	-129.10	-174.67	-165.81	-181.26	-181.66	-184.60	-177.44	-185.92						

Table S99: Correlation part of the interaction energies for different BNx_NBx dimers and (extrapolated) basis sets (cp-corrected) in kJ/mol using LNO-CCSD(T) with Tight criteria.

	DZ	aDZ	TZ	{D,T}Z	aTZ	a{D,T}Z	QZ	{T,Q}Z	aQZ	a{T,Q}Z	5Z	{Q,5}Z	a5Z	a{Q,5}Z
1	-15.39	-18.14	-17.53	-18.44	-19.05	-19.43	-18.95	-19.99	-19.34	-19.55	-19.24	-19.54	-19.42	-19.51
2	-28.08	-34.69	-34.47	-37.15	-36.56	-37.34	-36.37	-37.77	-36.80	-36.97	-36.81	-37.26	-37.20	-37.62
3	-42.47	-52.08	-51.98	-55.98	-54.40	-55.38	-54.60	-56.51	-55.26	-55.89	-54.93	-55.27	-55.52	-55.79
4	-57.33	-70.20	-70.49	-76.03	-73.26	-74.55	-73.36	-75.46	-73.86	-74.29	-73.67	-73.99	-74.16	-74.49
5	-69.93	-84.34	-84.08	-90.03	-88.12	-89.72	-88.15	-91.13	-88.77	-89.24	-88.32	-88.49	-89.14	-89.53
6	-84.14	-84.20	-102.48	-110.19	-107.32	-117.06	-107.51	-111.18	-108.15	-108.76	-107.64	-107.77	-108.49	-108.84

Table S100: Correlation part of the interaction energies for different BNx_NBx dimers and (extrapolated) basis sets (cp-corrected) in kJ/mol using LNO-CCSD(T) with very Tight criteria.

	DZ	aDZ	TZ	{D,T}Z	aTZ	a{D,T}Z	QZ	{T,Q}Z	aQZ	a{T,Q}Z	5Z	{Q,5}Z	a5Z	a{Q,5}Z
1	-13.07	-17.82	-16.78	-18.35	-18.89	-19.34	-18.27	-19.35	-19.29	-19.58	-18.84	-19.43	-19.18	-19.06
2	-25.95	-54.77	-32.82	-35.71	-35.99	-28.09	-35.16	-36.87	-36.68	-37.18	-36.04	-36.96	-36.55	-36.42
3	-39.54	-51.24	-49.55	-53.76	-53.79	-54.86	-52.74	-55.07	-54.91	-55.73	-53.92	-55.16	-54.50	-54.07
4	-54.04	-68.57	-66.72	-72.07	-71.90	-73.30	-70.81	-73.79	-73.57	-74.79	-72.21	-73.67	-73.02	-72.44
5	-65.39	-82.31	-80.15	-86.36	-86.12	-87.72	-87.96	-93.66						
6	-78.88	-78.94	-97.85	-105.83	-105.14	-116.17	-104.29	-108.99						

Table S101: Correlation part of the interaction energies for different BNx_BNx dimers and (extrapolated) basis sets (cp-corrected) in kJ/mol using LNO-CCSD(T) with Tight criteria.

	DZ	aDZ	TZ	{D,T}Z	aTZ	a{D,T}Z	QZ	{T,Q}Z	aQZ	a{T,Q}Z	5Z	{Q,5}Z	a5Z	a{Q,5}Z
1	-19.48	-23.42	-24.24	-26.24	-25.73	-26.70	-25.99	-27.26	-26.29	-26.70	-26.08	-26.18	-26.42	-26.56
2	-38.91	-48.13	-48.47	-52.50	-52.26	-53.99	-51.96	-54.50	-53.28	-54.03	-53.15	-54.41	-53.47	-53.67
3	-60.75	-74.16	-74.39	-80.13	-79.40	-81.60	-79.32	-82.92	-81.05	-82.26	-80.84	-82.44	-81.51	-81.99
4	-82.28	-99.95	-100.84	-108.65	-107.05	-110.04	-106.41	-110.48	-109.07	-110.54	-108.65	-111.00	-109.52	-110.00
5	-104.18	-126.15	-127.06	-136.69	-134.25	-137.66	-133.96	-138.99	-136.92	-138.87	-136.37	-138.89	-137.33	-137.77
6	-126.87	-152.43	-152.78	-163.69	-161.67	-165.57	-161.58	-167.99	-164.74	-166.97	-164.30	-167.15	-165.53	-166.37

Table S102: Correlation part of the interaction energies for different BNx_BNx dimers and (extrapolated) basis sets (cp-corrected) in kJ/mol using LNO-CCSD(T) with very Tight criteria.

	DZ	aDZ	TZ	{D,T}Z	aTZ	a{D,T}Z	QZ	{T,Q}Z	aQZ	a{T,Q}Z	5Z	{Q,5}Z	a5Z	a{Q,5}Z
1	-16.40	-23.54	-22.76	-25.44	-25.77	-26.71	-25.05	-26.71	-26.21	-26.53	-26.04	-27.08	-26.42	-26.63
2	-35.86	-48.33	-47.36	-52.20	-52.15	-53.77	-51.20	-54.01	-53.04	-53.68	-52.73	-54.32	-53.34	-53.66
3	-56.52	-74.00	-73.03	-79.98	-79.25	-81.46	-78.21	-81.99	-80.65	-81.67	-80.01	-81.90	-81.06	-81.49
4	-77.14	-99.47	-98.67	-107.73	-106.36	-109.27	-105.21	-109.99	-108.16	-109.47	-107.63	-110.16	-108.85	-109.57
5	-98.13	-125.29	-124.36	-135.40	-133.57	-137.05	-132.19	-137.91						
6	-118.96	-151.04	-150.15	-163.28	-161.10	-165.33	-160.38	-167.85						

Table S103: Correlation part of the interaction energies for different Acene PD dimers and (extrapolated) basis sets (non-cp-corrected) in kJ/mol using LNO-CCSD(T) with Tight criteria.

	DZ	aDZ	TZ	{D,T}Z	aTZ	a{D,T}Z	QZ	{T,Q}Z	aQZ	a{T,Q}Z	5Z	{Q,5}Z	a5Z	a{Q,5}Z
1	-22.71	-35.74	-27.47	-29.48	-32.01	-30.44	-27.99	-28.37	-29.40	-27.50	-28.26	-28.54	-28.46	-27.48
2	-49.39	-77.22	-58.99	-63.03	-67.60	-63.55	-59.22	-59.38	-61.19	-56.51	-59.32	-59.42	-59.37	-57.46
3	-77.18	-120.76	-91.72	-97.84	-104.97	-98.32	-91.31	-91.01	-94.14	-86.24	-91.37	-91.43	-91.40	-88.53
4	-106.04	-164.53	-124.76	-132.63	-142.36	-133.02	-123.87	-123.22	-127.62	-116.87	-124.52	-125.20	-123.77	-119.73
5	-134.57	-209.76	-159.12	-169.46	-181.05	-168.96	-157.52	-156.36	-162.02	-148.14	-157.31	-157.10	-157.89	-153.55
6	-163.18	-253.88	-192.61	-205.01	-218.38	-203.43	-190.60	-189.13	-195.79	-179.31	-190.44	-190.27	-190.45	-184.84

Table S104: Correlation part of the interaction energies for different Acene PD dimers and (extrapolated) basis sets (non-cp-corrected) in kJ/mol using LNO-CCSD(T) with very Tight criteria.

	DZ	aDZ	TZ	{D,T}Z	aTZ	a{D,T}Z	QZ	{T,Q}Z	aQZ	a{T,Q}Z	5Z	{Q,5}Z	a5Z	a{Q,5}Z
1	-21.90	-35.43	-26.96	-29.08	-31.75	-30.20	-27.50	-27.90	-28.95	-26.91	-27.76	-28.03	-28.10	-27.21
2	-48.07	-76.30	-57.86	-61.99	-66.88	-62.92	-57.99	-58.09	-60.32	-55.53	-58.13	-58.27	-58.57	-56.72
3	-75.03	-118.95	-89.85	-96.08	-103.42	-96.88	-89.46	-89.18	-92.45	-84.44	-89.34	-89.21	-89.85	-87.12
4	-102.43	-161.96	-122.34	-130.72	-140.41	-131.34	-121.41	-120.73	-125.11	-113.94	-121.17	-120.92	-121.71	-118.15
5	-130.48	-206.47	-155.52	-166.06	-178.09	-166.14	-154.37	-153.53						
6	-158.25	-250.34	-188.51	-201.26	-215.50	-200.83	-186.76	-185.48						

Table S105: Correlation part of the interaction energies for different BNx_NBx dimers and (extrapolated) basis sets (non-cp-corrected) in kJ/mol using LNO-CCSD(T) with Tight criteria.

	DZ	aDZ	TZ	{D,T}Z	aTZ	a{D,T}Z	QZ	{T,Q}Z	aQZ	a{T,Q}Z	5Z	{Q,5}Z	a5Z	a{Q,5}Z
1	-17.79	-27.26	-19.49	-20.21	-22.92	-21.09	-19.75	-19.94	-20.54	-18.80	-19.74	-19.73	-19.84	-19.10
2	-37.36	-55.37	-38.49	-38.96	-45.04	-40.70	-38.02	-37.67	-39.54	-35.53	-37.79	-37.54	-38.19	-36.76
3	-57.17	-85.22	-58.42	-58.95	-67.95	-60.68	-57.24	-56.37	-59.17	-52.75	-56.54	-55.80	-56.95	-54.62
4	-78.15	-116.34	-79.03	-79.41	-91.71	-81.34	-76.88	-75.31	-79.04	-69.79	-75.93	-74.92	-75.88	-72.57
5	-95.01	-141.79	-95.09	-95.12	-110.82	-97.77	-92.31	-90.28	-94.90	-83.29	-91.02	-89.67	-91.33	-87.59
6	-116.43	-116.45	-115.92	-115.70	-135.34	-143.29	-112.60	-110.18	-115.84	-101.62	-110.99	-109.30	-111.01	-105.94

Table S106: Correlation part of the interaction energies for different BNx_NBx dimers and (extrapolated) basis sets (non-cp-corrected) in kJ/mol using LNO-CCSD(T) with very Tight criteria.

	DZ	aDZ	TZ	{D,T}Z	aTZ	a{D,T}Z	QZ	{T,Q}Z	aQZ	a{T,Q}Z	5Z	{Q,5}Z	a5Z	a{Q,5}Z
1	-18.06	-26.98	-19.03	-19.44	-22.74	-20.96	-19.34	-19.56	-20.44	-18.75	-19.35	-19.36	-19.62	-18.76
2	-37.01	-54.77	-37.80	-38.13	-44.31	-39.91	-37.37	-37.06	-39.18	-35.44	-37.12	-36.85	-37.44	-35.62
3	-57.15	-84.33	-57.31	-57.38	-67.26	-60.08	-56.18	-55.36	-58.59	-52.26	-55.57	-54.92	-55.80	-52.86
4	-77.81	-114.84	-77.33	-77.13	-90.48	-80.22	-75.42	-74.02	-78.25	-69.33	-74.43	-73.39	-74.72	-71.02
5	-94.33	-139.92	-93.10	-92.58	-109.20	-96.26	-90.47	-88.55						
6	-115.40	-115.42	-113.65	-112.91	-133.23	-140.72	-110.90	-108.90						

Table S107: Correlation part of the interaction energies for different BNx_BNx dimers and (extrapolated) basis sets (non-cp-corrected) in kJ/mol using LNO-CCSD(T) with Tight criteria.

	DZ	aDZ	TZ	{D,T}Z	aTZ	a{D,T}Z	QZ	{T,Q}Z	aQZ	a{T,Q}Z	5Z	{Q,5}Z	a5Z	a{Q,5}Z
1	-25.34	-35.09	-27.30	-28.12	-31.04	-29.33	-26.88	-26.58	-27.98	-25.74	-26.84	-26.80	-27.12	-26.21
2	-56.07	-74.92	-56.78	-57.08	-64.40	-59.97	-55.17	-53.99	-57.14	-51.85	-54.62	-54.05	-55.09	-52.94
3	-87.95	-117.03	-87.26	-86.97	-98.17	-90.24	-84.19	-81.95	-86.70	-78.33	-83.09	-81.94	-83.66	-80.47
4	-120.22	-159.06	-117.76	-116.72	-132.53	-121.36	-113.21	-109.89	-116.46	-104.74	-111.76	-110.24	-112.11	-107.55
5	-152.38	-201.28	-148.00	-146.16	-166.56	-151.94	-142.22	-138.00	-145.82	-130.68	-140.27	-138.22	-140.66	-135.25
6	-184.88	-243.43	-178.65	-176.02	-200.56	-182.51	-171.34	-166.01	-175.41	-157.05	-168.97	-166.48	-169.37	-163.04

Table S108: Correlation part of the interaction energies for different BNx_BNx dimers and (extrapolated) basis sets (non-cp-corrected) in kJ/mol using LNO-CCSD(T) with very Tight criteria.

	DZ	aDZ	TZ	{D,T}Z	aTZ	a{D,T}Z	QZ	{T,Q}Z	aQZ	a{T,Q}Z	5Z	{Q,5}Z	a5Z	a{Q,5}Z
1	-25.55	-35.41	-27.21	-27.90	-31.09	-29.27	-26.83	-26.55	-27.86	-25.51	-26.73	-26.62	-27.05	-26.20
2	-55.98	-75.05	-56.47	-56.67	-64.00	-59.34	-54.82	-53.62	-56.60	-51.20	-54.24	-53.63	-54.60	-52.50
3	-88.00	-116.55	-86.77	-86.25	-98.05	-90.26	-83.82	-81.66	-85.88	-77.00	-82.44	-80.99	-82.97	-79.92
4	-120.11	-158.47	-117.00	-115.70	-132.11	-121.02	-112.58	-109.35	-115.02	-102.55	-110.76	-108.85	-111.29	-107.38
5	-152.32	-200.56	-147.26	-145.12	-166.14	-151.64	-141.44	-137.20						
6	-184.59	-242.52	-177.64	-174.72	-200.23	-182.42	-171.32	-166.71						

Table S109: cp-corrected Slopes (in kJ/mol per ring) and Intercepts (in kJ/mol) for Figure 1.

Subfigure		basis	Slope	Intercept
a	cp-corrected	DZ	-23.08	4.2
		aDZ	-30.54	5.2
		TZ	-29.50	5.1
		aTZ	-31.65	5.5
		QZ	-31.17	5.4
		aQZ	-31.77	5.1
		5Z	-31.80	4.9
		a5Z	-31.81	4.9
b	cp-corrected	DZ	-13.83	-1.1
		aDZ	-14.21	-7.5
		TZ	-16.92	-1.0
		aTZ	-17.57	-1.6
		QZ	-17.63	-1.5
		aQZ	-17.67	-1.8
		5Z	-17.58	-1.9
		a5Z	-17.71	-2.0
c	cp-corrected	DZ	-21.55	3.4
		aDZ	-25.85	3.1
		TZ	-25.85	2.5
		aTZ	-27.24	1.9
		QZ	-27.17	1.9
		aQZ	-27.75	1.9
		5Z	-27.67	2.0
		a5Z	-27.86	1.9

Table S110: cp-uncorrected Slopes (in kJ/mol per ring) and Intercepts (in kJ/mol) for Figure 1.

Subfigure		basis	Slope	Intercept
d	cp-uncorrected	DZ	-28.19	6.5
		aDZ	-43.77	9.6
		TZ	-33.12	6.8
		aTZ	-37.42	6.6
		QZ	-32.59	5.6
		aQZ	-33.37	5.1
		5Z	-32.51	5.3
		a5Z	-32.51	5.2
e	cp-uncorrected	DZ	-19.63	1.7
		aDZ	-21.04	-16.8
		TZ	-19.22	-0.5
		aTZ	-22.38	-0.6
		QZ	-18.48	-1.5
		aQZ	-18.93	-1.9
		5Z	-18.15	-1.8
		a5Z	-18.12	-2.1
f	cp-uncorrected	DZ	-31.97	7.4
		aDZ	-42.13	9.4
		TZ	-30.31	3.5
		aTZ	-33.96	3.3
		QZ	-28.93	2.4
		aQZ	-29.51	1.7
		5Z	-28.46	2.0
		a5Z	-28.47	1.6

Table S111: cp-corrected Slopes (in kJ/mol per ring) and Intercepts (in kJ/mol) for Figure 2.

Subfigure		CCSD(T) method	Slope	Intercept
a	DZ cp-corrected	Canonical	-21.89	4.4
		LNO vvTight	-22.05	4.4
		LNO vTight	-22.19	4.5
		LNO Tight	-23.08	4.2
		LNO Normal	-25.81	4.8
		DLPNO Tcut=1e-6	-21.68	4.7
		DLPNO Tcut=1e-7	-22.60	5.2
		PNO DOMOPT Tight	-20.82	3.3
		PNO DOMOPT vTight	-21.16	3.5
		PNO PAIROPT Tight	-20.12	2.7
b	DZ cp-corrected	Canonical	-13.11	-0.1
		LNO vvTight	-13.21	0.02
		LNO vTight	-13.20	0.04
		LNO Tight	-13.83	-1.1
		LNO Normal	-15.99	0.6
		DLPNO Tcut=1e-6	-12.79	0.8
		DLPNO Tcut=1e-7	-13.42	0.6
		PNO DOMOPT Tight	-12.69	-0.3
		PNO DOMOPT vTight	-13.10	-0.2
		PNO PAIROPT Tight	-12.40	-0.4
c	DZ cp-corrected	Canonical	-20.72	4.6
		LNO vvTight	-20.68	4.8
		LNO vTight	-20.58	4.9
		LNO Tight	-21.55	3.4
		LNO Normal	-23.83	3.8
		DLPNO Tcut=1e-6	-19.32	4.7
		DLPNO Tcut=1e-7	-20.82	5.4
		PNO DOMOPT Tight	-20.27	5.0
		PNO DOMOPT vTight	-20.83	4.9
		PNO PAIROPT Tight	-19.18	5.1

Table S112: cp-uncorrected Slopes (in kJ/mol per ring) and Intercepts (in kJ/mol) for Figure 2.

Subfigure		CCSD(T) method	Slope	Intercept
d	DZ cp-uncorrected	Canonical	-26.98	6.3
		LNO vvTight	-27.19	6.2
		LNO vTight	-27.33	6.3
		LNO Tight	-28.19	6.5
		LNO Normal	-30.50	7.1
		DLPNO Tcut=1e-6	-25.99	5.8
		DLPNO Tcut=1e-7	-26.95	6.4
		PNO DOMOPT Tight	-24.77	4.0
		PNO DOMOPT vTight	-25.15	4.2
		PNO PAIROPT Tight	-22.32	2.8
e	DZ cp-uncorrected	Canonical	-19.50	1.0
		LNO vvTight	-19.39	1.2
		LNO vTight	-19.41	1.3
		LNO Tight	-19.63	1.7
		LNO Normal	-19.89	1.1
		DLPNO Tcut=1e-6	-17.75	0.4
		DLPNO Tcut=1e-7	-18.34	0.3
		PNO DOMOPT Tight	-16.63	-1.3
		PNO DOMOPT vTight	-17.02	-1.2
		PNO PAIROPT Tight	-14.75	-1.8
f	DZ cp-uncorrected	Canonical	-32.22	7.2
		LNO vvTight	-31.98	7.1
		LNO vTight	-31.90	7.2
		LNO Tight	-31.97	7.4
		LNO Normal	-32.65	7.7
		DLPNO Tcut=1e-6	-29.48	5.5
		DLPNO Tcut=1e-7	-30.91	6.6
		PNO DOMOPT Tight	-29.33	5.4
		PNO DOMOPT vTight	-29.89	5.3
		PNO PAIROPT Tight	-23.93	3.9

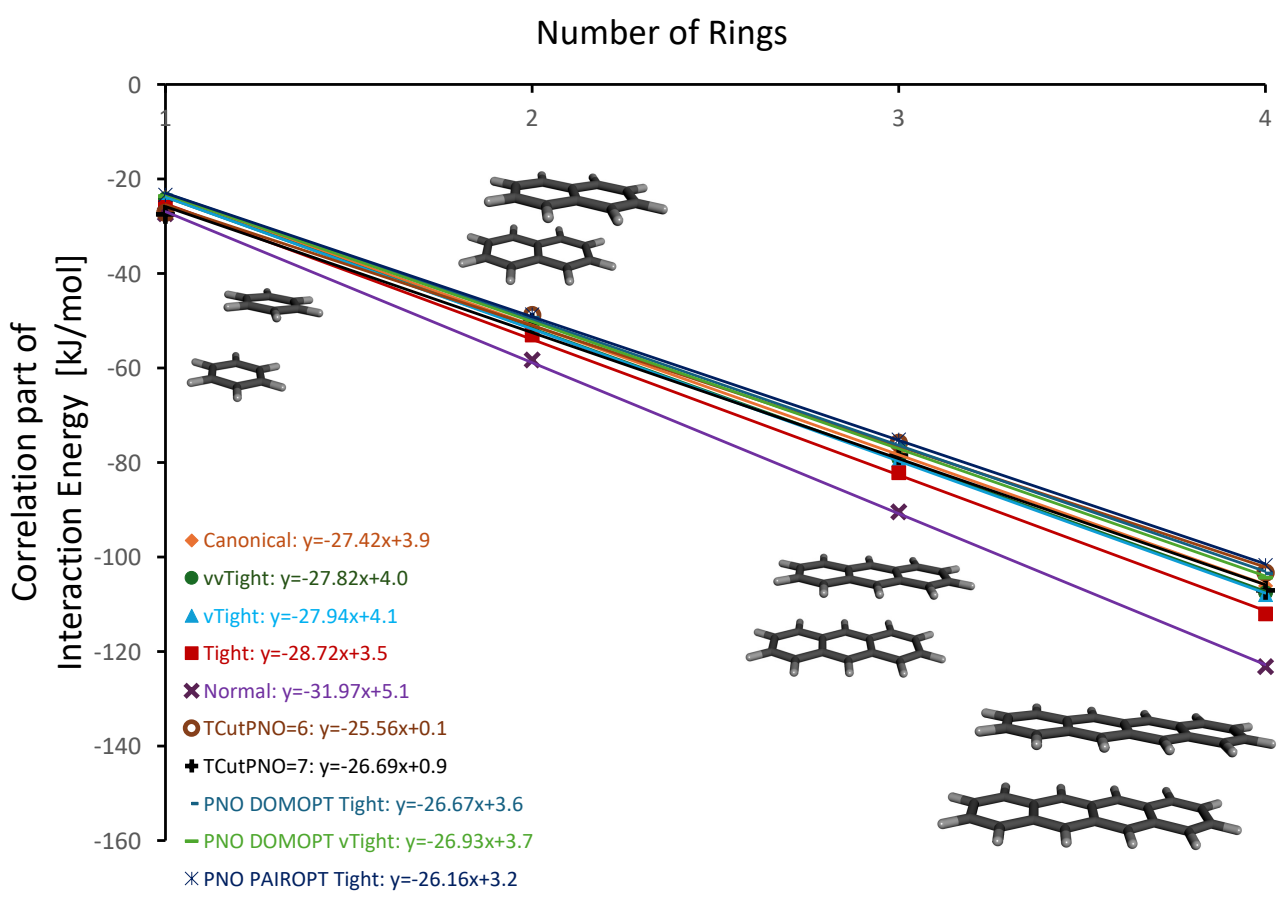

(a) cp-corr TZ energies for acenes PD

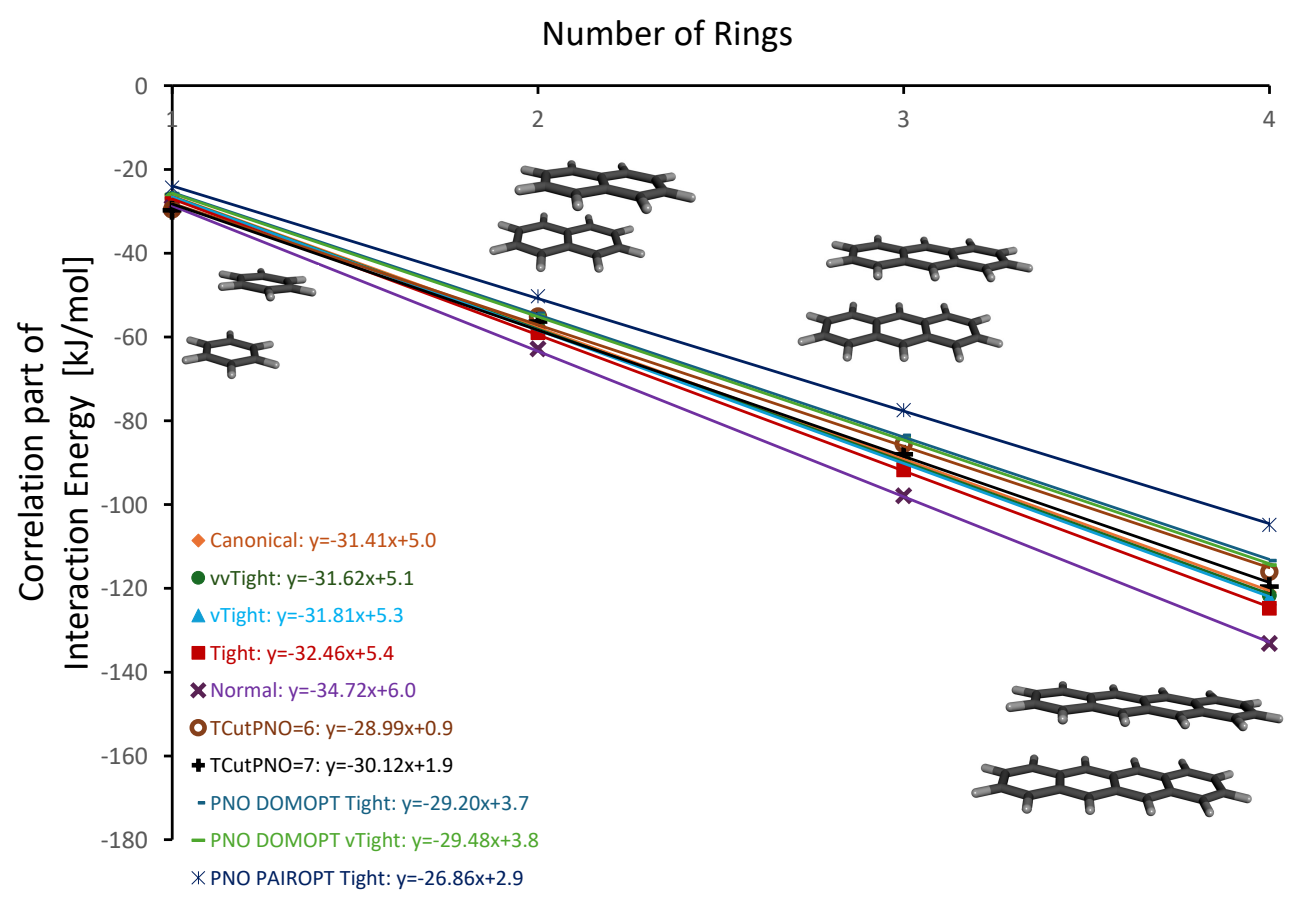

(d) cp-uncorr TZ energies for acenes PD

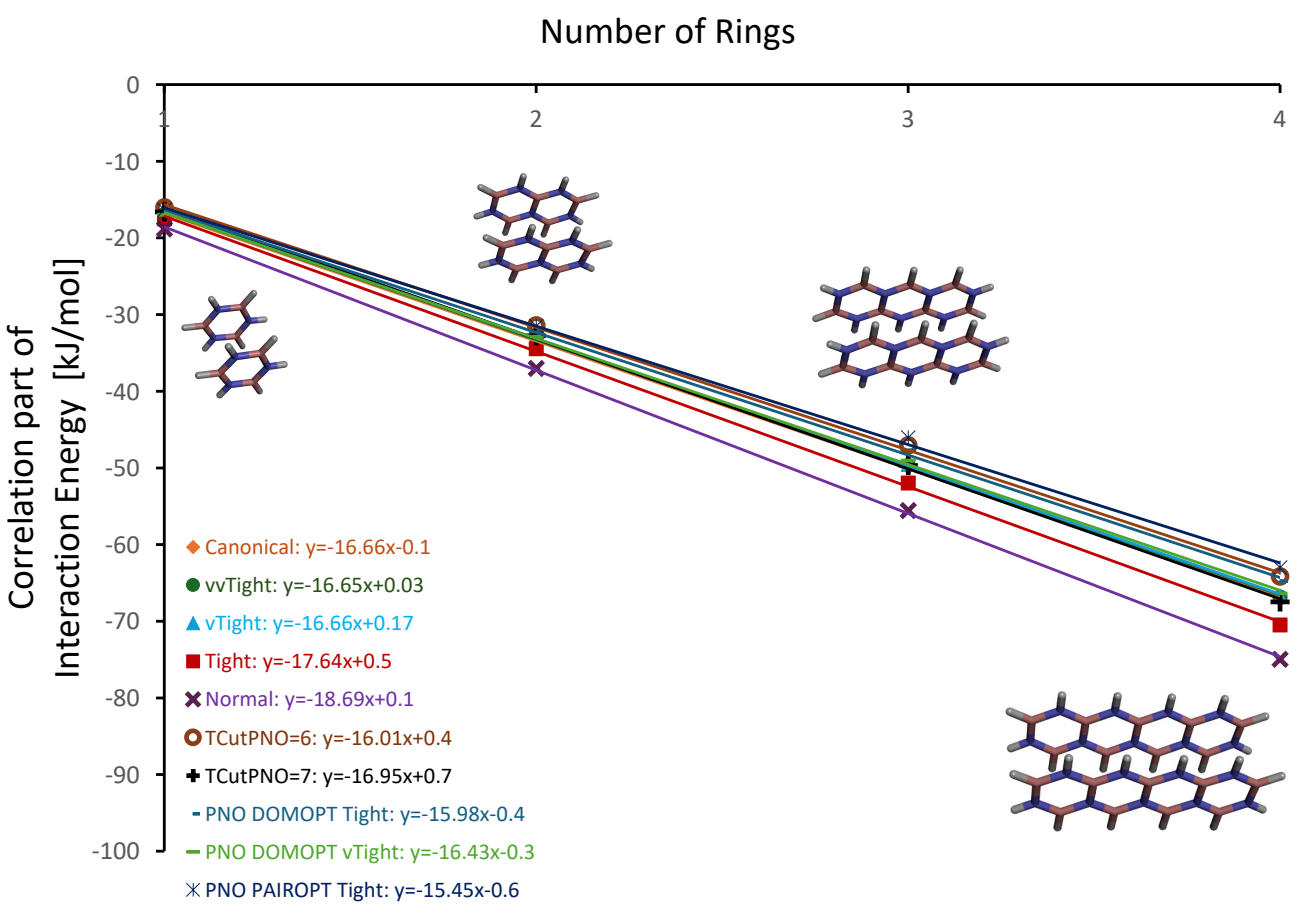

(b) cp-corr TZ energies for BNx ⋯ NBx

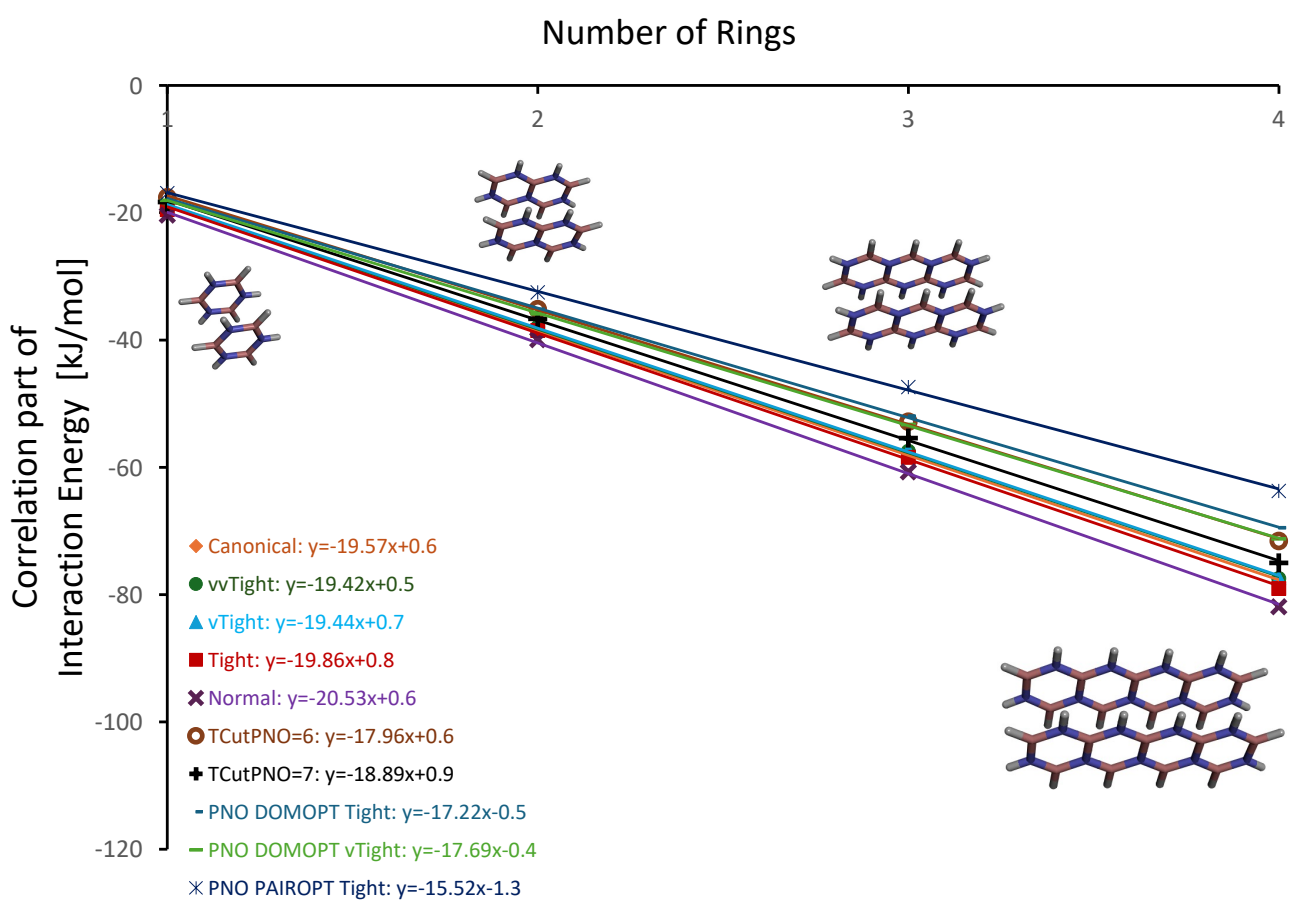

(e) cp-uncorr TZ energies for BNx ⋯ NBx

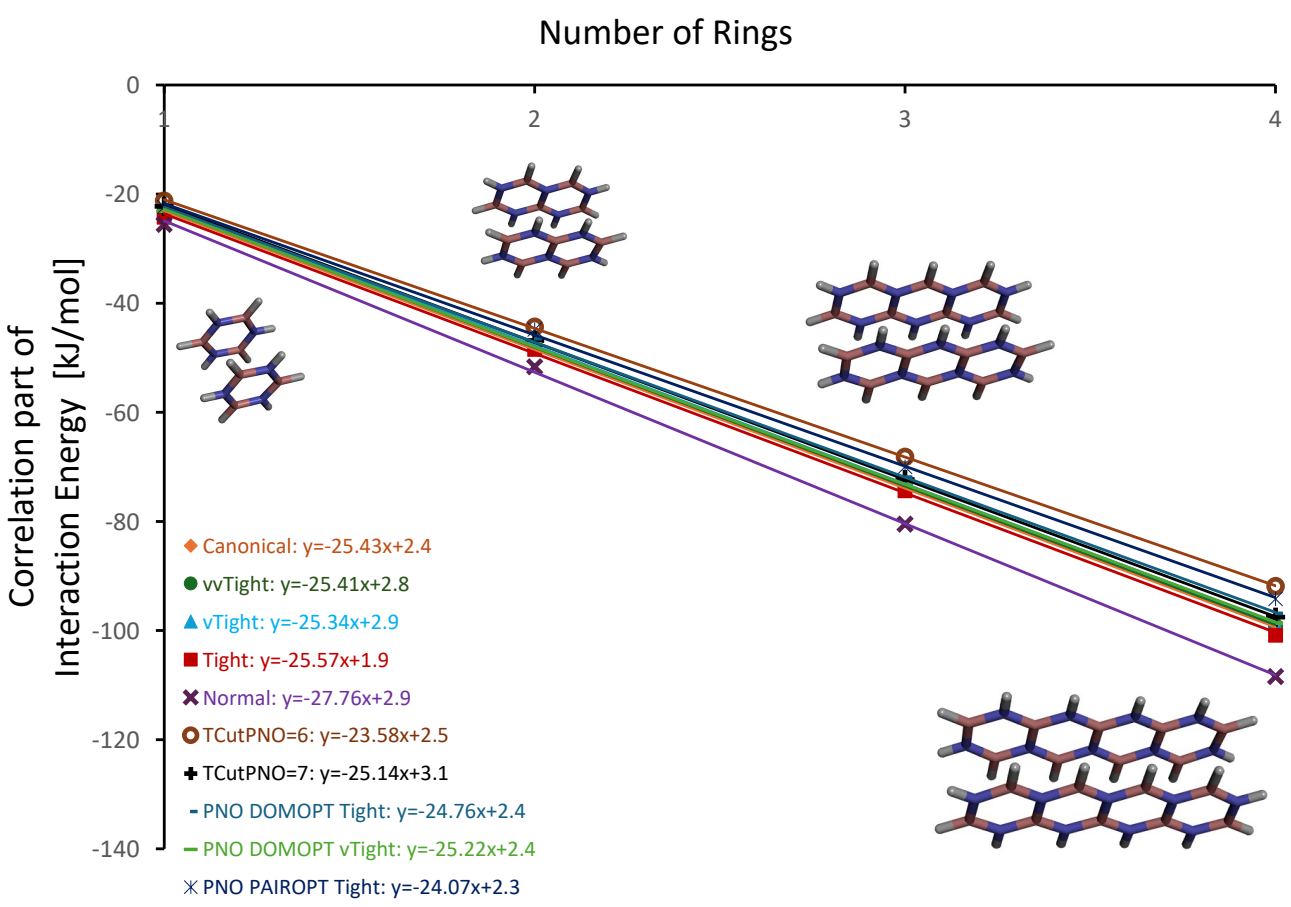

(c) cp-corr TZ energies for BNx ⋯ BNx

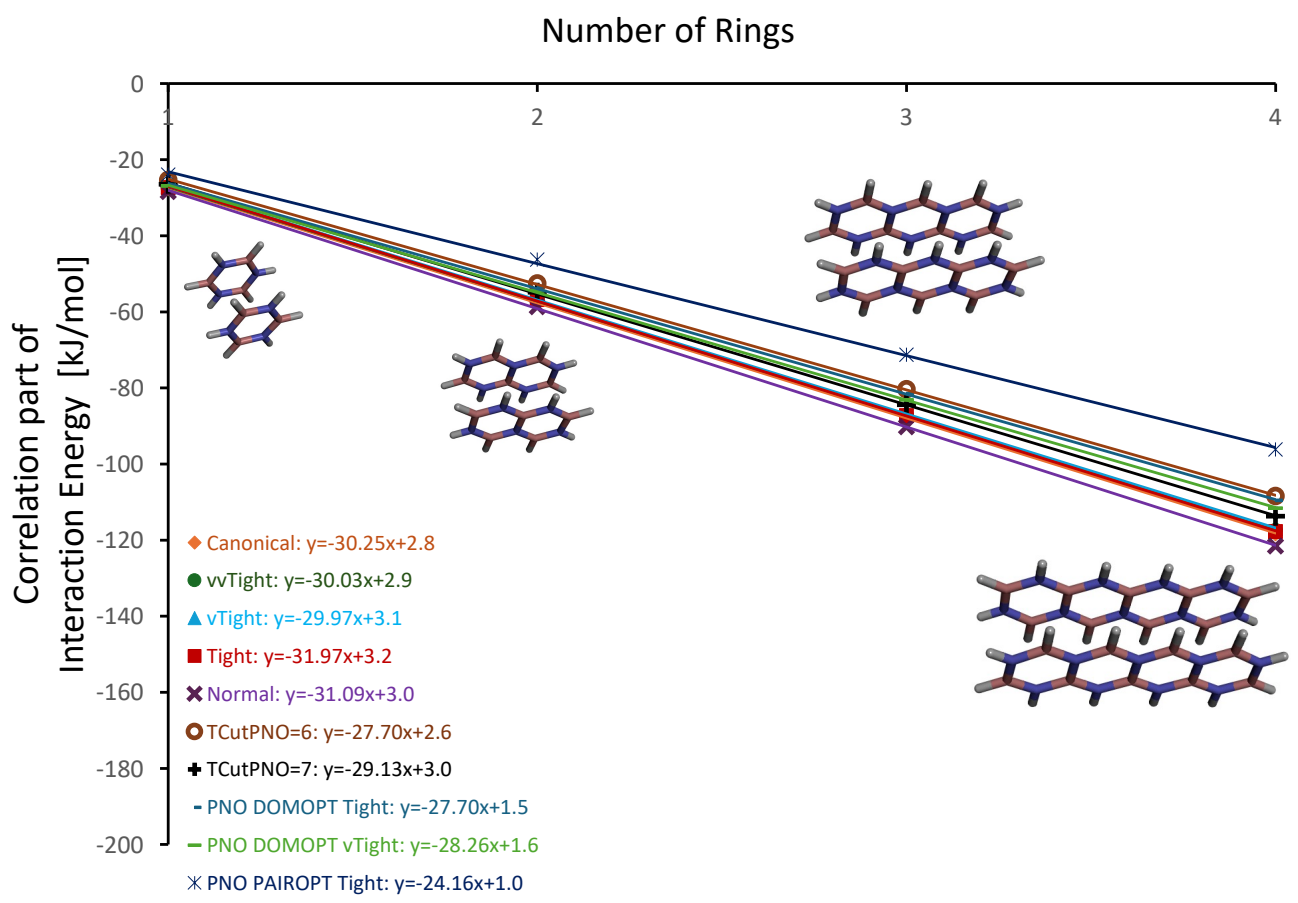

(f) cp-uncorr TZ energies for BNx ⋯ BNx

Figure S1: Correlation part of interaction energies (kJ/mol) derived from various coupled cluster methods in as a function of system size per number of rings using cc-pVTZ basis set.

Raw Hartree-Fock, RPA and DFT-SAPT Data: Basis Set Effects

All raw Hartree-Fock, RPA (which uses the PBE exchange functional), as well as DFT-SAPT exchange part are reported in this section. Concerning DFT-SAPT, we are using the PBE0-AC functional with electrostatic+exchange+induction+dHF contributions, whereas the asymptotic correction for the PBE0 functional has been determined by PBE0 ionization potentials and orbital energies of the individual monomers.

Table S113: Hartree-Fock, RPA, DFT-SAPT(electrostatic+exchange+induction) part of the interaction energies in kJ/mol, cp-corrected cc-pVDZ basis set for the acene PD series.

DZ	method		
System	HF	DFT-SAPT ele	RPA exch
c1_c1_pd	16.66	15.41	16.82
c2_c2_pd	30.90	30.55	31.15
c3_c3_pd	44.75	45.78	45.20
c4_c4_pd	58.65	61.14	59.21
c5_c5_pd	72.88	77.12	73.87
c6_c6_pd	86.76	92.75	88.34

Table S114: Hartree-Fock, RPA, DFT-SAPT(electrostatic+exchange+induction) part of the interaction energies in kJ/mol, cp-corrected cc-pVTZ basis set for the acene PD series.

TZ	method		
System	HF	DFT-SAPT ele	RPA exch
c1_c1_pd	16.32	15.10	16.84
c2_c2_pd	31.01	30.38	31.97
c3_c3_pd	45.39	45.82	46.94
c4_c4_pd	59.70	61.42	61.93
c5_c5_pd	74.55	77.68	77.65
c6_c6_pd	89.06	93.60	93.16

Table S115: Hartree-Fock, RPA, DFT-SAPT(electrostatic+exchange+induction) part of the interaction energies in kJ/mol, cp-corrected cc-pVQZ basis set for the acene PD series.

QZ	method		
System	HF	DFT-SAPT ele	RPA exch
c1_c1_pd	16.24	15.04	16.84
c2_c2_pd	30.89	30.27	32.03
c3_c3_pd	45.26	45.65	47.05
c4_c4_pd	59.54	61.20	62.09
c5_c5_pd	74.36	77.39	77.83
c6_c6_pd	88.84		93.39

Table S116: Hartree-Fock, RPA, DFT-SAPT(electrostatic+exchange+induction) part of the interaction energies in kJ/mol, cp-corrected cc-pV5Z basis set for the acene PD series.

5Z	method		
System	HF	DFT-SAPT ele	RPA exch
c1_c1_pd	16.12	14.88	16.69
c2_c2_pd	30.71	30.00	
c3_c3_pd	45.04	45.26	
c4_c4_pd	59.29	60.69	
c5_c5_pd	74.07		
c6_c6_pd	88.51		

Table S117: Hartree-Fock part of the interaction energies in kJ/mol, cp-corrected aug-cc-pVnZ basis set for the acene PD series.

System	aDZ	aTZ	aQZ	a5Z
c1_c1_pd	16.20	16.12	16.11	16.11
c2_c2_pd	30.84	30.72	30.71	30.70
c3_c3_pd	45.23	45.05	45.04	45.03
c4_c4_pd	59.57	59.31	59.29	59.28
c5_c5_pd	74.40	74.09	74.07	74.05
c6_c6_pd	88.92	88.54	88.52	88.50

Table S118: Hartree-Fock, RPA, DFT-SAPT(electrostatic+exchange+induction) part of the interaction energies in kJ/mol, cp-corrected cc-pVDZ basis set for the BNx_NBx series.

DZ	method		
System	HF	DFT-SAPT ele	RPA exch
bn1_nb1	11.46	12.63	13.75
bn2_nb2	21.66	24.27	25.46
bn3_nb3	33.17	37.31	38.49
bn4_nb4	45.20	51.02	52.14
bn5_nb5	52.22	59.64	60.26
bn6_nb6	64.94	74.31	74.73

Table S119: Hartree-Fock, RPA, DFT-SAPT(electrostatic+exchange+induction) part of the interaction energies in kJ/mol, cp-corrected cc-pVTZ basis set for the BNx_NBx series.

TZ	method		
System	HF	DFT-SAPT ele	RPA exch
bn1_nb1	11.73	12.74	14.04
bn2_nb2	22.01	24.44	25.91
bn3_nb3	33.49	37.46	38.99
bn4_nb4	45.48	51.15	52.65
bn5_nb5	52.45	59.69	60.80
bn6_nb6	65.11	74.32	75.27

Table S120: Hartree-Fock, RPA, DFT-SAPT(electrostatic+exchange+induction) part of the interaction energies in kJ/mol, cp-corrected cc-pVQZ basis set for the BNx_NBx series.

QZ	method		
System	HF	DFT-SAPT ele	RPA exch
bn1_nb1	11.77	12.73	14.13
bn2_nb2	22.09	24.45	26.07
bn3_nb3	33.58	37.48	39.17
bn4_nb4	45.56	51.17	52.85
bn5_nb5	52.49	59.67	60.98
bn6_nb6	65.13		75.47

Table S121: Hartree-Fock, RPA, DFT-SAPT(electrostatic+exchange+induction) part of the interaction energies in kJ/mol, cp-corrected cc-pV5Z basis set for the BNx_NBx series.

5Z	method	
System	HF	DFT-SAPT ele
bn1_nb1	11.81	12.75
bn2_nb2	22.12	24.48
bn3_nb3	33.60	37.50
bn4_nb4	45.57	
bn5_nb5	52.47	
bn6_nb6	65.10	

Table S122: Hartree-Fock part of the interaction energies in kJ/mol, cp-corrected aug-cc-pVnZ basis set for the BNx_NBx series.

System	aDZ	aTZ	aQZ	a5Z
bn1_nb1	11.93	11.81	11.81	11.81
bn2_nb2	22.14	22.11	22.13	22.12
bn3_nb3	33.54	33.59	33.61	33.60
bn4_nb4	45.43	45.55	45.58	45.57
bn5_nb5	52.19	52.45	52.48	52.47
bn6_nb6	64.94	65.07	65.11	65.10

Table S123: Hartree-Fock, RPA, DFT-SAPT(electrostatic+exchange+induction) part of the interaction energies in kJ/mol, cp-corrected cc-pVDZ basis set for the BNx_BNx series.

DZ	method		
System	HF	DFT-SAPT ele	RPA exch
bn1_bn1	13.99	22.10	19.82
bn2_bn2	26.51	42.18	36.92
bn3_bn3	38.24	61.22	53.90
bn4_bn4	49.55	79.76	70.46
bn5_bn5	60.78	98.32	86.84
bn6_bn6	72.07	117.05	103.26

Table S124: Hartree-Fock, RPA, DFT-SAPT(electrostatic+exchange+induction) part of the interaction energies in kJ/mol, cp-corrected cc-pVTZ basis set for the BNx_BNx series.

TZ	method		
System	HF	DFT-SAPT ele	RPA exch
bn1_bn1	12.57	20.50	17.91
bn2_bn2	24.31	39.85	33.90
bn3_bn3	35.43	58.34	49.86
bn4_bn4	46.22	76.43	65.51
bn5_bn5	56.95	94.57	81.03
bn6_bn6	67.75	112.89	96.60

Table S125: Hartree-Fock, RPA, DFT-SAPT(electrostatic+exchange+induction) part of the interaction energies in kJ/mol, cp-corrected cc-pVQZ basis set for the BNx_BNx series.

QZ	method		
System	HF	DFT-SAPT ele	RPA exch
bn1_bn1	12.31	20.31	17.61
bn2_bn2	24.01	39.72	33.60
bn3_bn3	35.10	58.28	49.55
bn4_bn4	45.87	76.46	65.21
bn5_bn5	56.58	94.71	80.75
bn6_bn6	67.36		96.32

Table S126: Hartree-Fock, RPA, DFT-SAPT(electrostatic+exchange+induction) part of the interaction energies in kJ/mol, cp-corrected cc-pV5Z basis set for the BNx_BNx series.

5Z	method	
System	HF	DFT-SAPT ele
bn1_bn1	12.30	20.31
bn2_bn2	24.02	39.74
bn3_bn3	35.13	
bn4_bn4	45.90	
bn5_bn5	56.63	
bn6_bn6	67.43	

Table S127: Hartree-Fock part of the interaction energies in kJ/mol, cp-corrected aug-cc-pVnZ basis set for the BNx_BNx series.

System	aDZ	aTZ	aQZ	a5Z
bn1_bn1	12.69	12.37	12.29	12.28
bn2_bn2	24.68	24.14	24.02	24.00
bn3_bn3	36.04	35.28	35.12	35.10
bn4_bn4	47.05	46.08	45.89	45.86
bn5_bn5	58.00	56.84	56.62	56.58
bn6_bn6	69.02	67.66	67.41	67.37

Table S128: Hartree-Fock and RPA part of the interaction energies in kJ/mol, cp-uncorrected cc-pVDZ basis set for the acene PD series.

DZ	method	
System	HF	RPA exch
c1_c1_pd	11.89	12.39
c2_c2_pd	23.09	23.96
c3_c3_pd	33.81	35.18
c4_c4_pd	44.21	46.35
c5_c5_pd	55.05	58.08
c6_c6_pd	65.56	69.63

Table S129: Hartree-Fock and RPA part of the interaction energies in kJ/mol, cp-uncorrected cc-pVTZ basis set for the acene PD series.

TZ	method	
System	HF	RPA exch
c1_c1_pd	14.36	15.12
c2_c2_pd	28.04	29.47
c3_c3_pd	41.45	43.70
c4_c4_pd	54.74	57.92
c5_c5_pd	68.55	72.87
c6_c6_pd	82.02	87.60

Table S130: Hartree-Fock and RPA part of the interaction energies in kJ/mol, cp-uncorrected cc-pVQZ basis set for the acene PD series.

QZ	method	
System	HF	RPA exch
c1_c1_pd	15.39	16.26
c2_c2_pd	29.63	31.33
c3_c3_pd	43.62	46.29
c4_c4_pd	57.53	61.25
c5_c5_pd	71.95	76.93
c6_c6_pd	86.04	92.42

Table S131: Hartree-Fock and RPA part of the interaction energies in kJ/mol, cp-uncorrected cc-pV5Z basis set for the acene PD series.

5Z	method	
System	HF	RPA exch
c1_c1_pd	15.95	16.61
c2_c2_pd	30.48	
c3_c3_pd	44.74	
c4_c4_pd	58.93	
c5_c5_pd	73.65	
c6_c6_pd	88.03	

Table S132: Hartree-Fock part of the interaction energies in kJ/mol, cp-uncorrected aug-cc-pVnZ basis set for the acene PD series.

System	aDZ	aTZ	aQZ	a5Z
c1_c1_pd	10.62	15.18	15.86	16.07
c2_c2_pd	18.82	28.85	30.25	30.65
c3_c3_pd	26.41	42.29	44.40	44.95
c4_c4_pd	34.13	55.67	58.48	59.18
c5_c5_pd	42.22	69.57	73.08	73.93
c6_c6_pd	50.00	83.15	87.35	88.35

Table S133: Hartree-Fock and RPA part of the interaction energies in kJ/mol, cp-uncorrected cc-pVDZ basis set for the BNx_NBx series.

DZ	method	
System	HF	RPA exch
bn1_nb1	6.61	9.35
bn2_nb2	12.59	17.15
bn3_nb3	19.94	26.40
bn4_nb4	27.87	36.37
bn5_nb5	31.98	42.01
bn6_nb6	40.61	52.85

Table S134: Hartree-Fock and RPA part of the interaction energies in kJ/mol, cp-uncorrected cc-pVTZ basis set for the BNx_NBx series.

TZ	method	
System	HF	RPA exch
bn1_nb1	9.50	11.70
bn2_nb2	18.29	22.12
bn3_nb3	28.40	33.91
bn4_nb4	39.05	46.34
bn5_nb5	44.98	53.55
bn6_nb6	56.31	66.80

Table S135: Hartree-Fock and RPA part of the interaction energies in kJ/mol, cp-uncorrected cc-pVQZ basis set for the BNx_NBx series.

QZ	method	
System	HF	RPA exch
bn1_nb1	10.84	13.15
bn2_nb2	20.60	24.61
bn3_nb3	31.59	37.32
bn4_nb4	43.08	50.63
bn5_nb5	49.65	58.49
bn6_nb6	61.81	72.60

Table S136: Hartree-Fock part of the interaction energies in kJ/mol, cp-uncorrected cc-pV5Z basis set for the BNx_NBx series.

5Z	method
System	HF
bn1_nb1	11.66
bn2_nb2	21.88
bn3_nb3	33.27
bn4_nb4	45.16
bn5_nb5	51.98
bn6_nb6	64.52

Table S137: Hartree-Fock part of the interaction energies in kJ/mol, cp-uncorrected aug-cc-pVnZ basis set for the BNx_NBx series.

System	aDZ	aTZ	aQZ	a5Z
bn1_nb1	7.40	10.97	11.57	11.78
bn2_nb2	12.16	20.32	21.69	22.07
bn3_nb3	18.01	30.77	33.02	33.52
bn4_nb4	24.26	41.79	44.86	45.46
bn5_nb5	26.15	47.80	51.63	52.34
bn6_nb6	40.61	59.43	64.12	64.95

Table S138: Hartree-Fock and RPA part of the interaction energies in kJ/mol, cp-uncorrected cc-pVDZ basis set for the BNx_BNx series.

DZ	method	
System	HF	RPA exch
bn1_bn1	3.17	8.60
bn2_bn2	8.24	18.20
bn3_bn3	13.01	28.30
bn4_bn4	17.52	38.21
bn5_bn5	21.97	47.96
bn6_bn6	26.48	57.73

Table S139: Hartree-Fock and RPA part of the interaction energies in kJ/mol, cp-uncorrected cc-pVTZ basis set for the BNx_BNx series.

TZ	method	
System	HF	RPA exch
bn1_bn1	8.77	13.69
bn2_bn2	18.17	27.27
bn3_bn3	27.19	41.21
bn4_bn4	35.98	54.97
bn5_bn5	44.75	68.62
bn6_bn6	53.56	82.30

Table S140: Hartree-Fock and RPA part of the interaction energies in kJ/mol, cp-uncorrected cc-pVQZ basis set for the BNx_BNx series.

QZ	method	
System	HF	RPA exch
bn1_bn1	10.75	15.90
bn2_bn2	21.60	31.16
bn3_bn3	31.92	46.52
bn4_bn4	41.95	61.62
bn5_bn5	51.93	76.60
bn6_bn6	61.98	91.61

Table S141: Hartree-Fock part of the interaction energies in kJ/mol, cp-uncorrected cc-pV5Z basis set for the BNx_BNx series.

5Z	method
System	HF
bn1_bn1	12.06
bn2_bn2	23.68
bn3_bn3	34.68
bn4_bn4	45.36
bn5_bn5	55.99
bn6_bn6	66.68

Table S142: Hartree-Fock part of the interaction energies in kJ/mol, cp-uncorrected aug-cc-pVnZ basis set for the BNx_BNx series.

System	aDZ	aTZ	aQZ	a5Z
bn1_bn1	7.37	11.30	12.00	12.24
bn2_bn2	12.59	21.83	18.55	23.92
bn3_bn3	17.19	31.76	34.43	34.98
bn4_bn4	21.46	41.40	45.05	45.72
bn5_bn5	25.73	51.01	55.61	56.41
bn6_bn6	30.09	60.68	66.24	67.16

Electronic Structure Methods

In this section, we show all raw data for the different series using different methods (other than CCSD(T)) and basis sets for the acene PD, BNx_NBx and BNx_BNx series. For DFT-SAPT using the PBE0-AC functional as underlying method, the dispersion contribution (including exchange-dispersion) is reported.

Table S143: Interaction energies (in kJ/mol) for different methods for the acene PD series using a cp-corrected cc-pVDZ basis set.

	Cx_Cx PD		correlation								
System	Rings	HF	MP2	MP3	MP2.5	dRPA	LNO-CCSD(T)	DFT-SAPT	CCSD	CCSD(T)	CCSD(cT-fit)
							vvTight	disp			
c_c	1	16.66	-24.43	-15.36	-19.89	-16.98	-18.36	-17.11	-15.55	-18.28	-17.80
c2_c2	2	30.90	-55.31	-29.82	-42.56	-36.66	-39.35	-38.14	-32.77	-39.03	-37.80
c3_c3	3	44.75	-88.39	-43.25	-65.82	-57.21	-61.20	-60.05	-50.55	-60.61	-58.52
c4_c4	4	58.65	-122.66	-56.26	-89.46	-78.20	-83.40	-82.41	-68.72	-82.74	-79.74
c5_c5	5	72.88	-157.76	-68.66	-113.21	-99.73	-105.94	-105.48	-87.08	-105.15	-101.20
c6_c6	6	86.76	-192.63	-80.40	-136.51	-121.21	-128.33	-128.37	-105.28	-127.42	-122.51

Table S144: Interaction energies (in kJ/mol) for post-CCSD(T) methods for the acene PD series using a cp-corrected cc-pVDZ basis set.

DZ	Cx_Cx PD	correlation				
System	Rings	CCSD(T)-L	CCSDT-2	CCSDT-3	CCSDT	CCSDT(Q)
c_c	1	-18.07	-17.80	-17.81	-17.61	-17.99
c2_c2	2	-38.70	-37.98	-38.04		
c3_c3	3	-60.11				
c4_c4	4	-81.88				

Table S145: Interaction energies (in kJ/mol) for different methods for the acene PD series using a cp-corrected cc-pVTZ basis set.

	Cx_Cx PD		correlation								
System	Rings	HF	MP2	MP3	MP2.5	dRPA	LNO-CCSD(T)	DFT-SAPT	CCSD	CCSD(T)	CCSD(cT-fit)
							vvTight	disp			
c_c	1	16.32	-31.73	-19.82	-25.77	-22.02	-24.17	-23.38	-19.77	-23.87	-23.12
c2_c2	2	31.01	-70.34	-37.92	-54.13	-46.81	-51.14	-50.14	-41.42	-50.53	-48.72
c3_c3	3	45.39	-111.40	-54.79	-83.09	-72.59	-79.19	-77.70	-63.65	-78.08	-75.08
c4_c4	4	59.70	-153.64	-71.02	-112.33	-98.86	-107.56	-105.77	-86.20	-106.10	-101.85
c5_c5	5	74.55				-125.88		-134.64			
c6_c6	6	89.06				-152.73		-163.30			

Table S146: Interaction energies (in kJ/mol) for different methods for the acene PD series using a cp-corrected cc-pVQZ basis set.

	Cx_Cx PD		correlation								
System	Rings	HF	MP2	MP3	MP2.5	dRPA	LNO-CCSD(T)	DFT-SAPT	CCSD	CCSD(T)	CCSD(cT-fit)
							vvTight	disp			
c_c	1	16.24	-34.11	-21.70	-27.91	-23.96	-26.20	-25.70	-21.36	-26.03	-25.19
c2_c2	2	30.89	-74.85	-41.23	-58.04	-50.48	-54.92	-54.28	-44.35	-54.57	-52.55
c3_c3	3	45.26				-77.88		-83.61			
c4_c4	4	59.54				-105.83		-113.47			
c5_c5	5	74.36				-134.53		-144.16			
c6_c6	6	88.84									

Table S147: Interaction energies (in kJ/mol) for different methods for the acene PD series using a cp-corrected cc-pV5Z basis set.

	Cx_Cx PD		correlation								
System	Rings	HF	MP2	MP3	MP2.5	dRPA	LNO-CCSD(T)	DFT-SAPT	CCSD	CCSD(T)	CCSD(cT-fit)
							vvTight	disp			
c_c	1	16.12	-35.12			-24.92	-27.11	-26.75	-22.05	-26.97	-26.08
c2_c2	2	30.71					-56.54	-55.99			
c3_c3	3	45.04						-85.97			
c4_c4	4	59.29						-116.48			
c5_c5	5	74.07									
c6_c6	6	88.51									

Table S148: Interaction energies (in kJ/mol) for different methods for the acene PD series using a cp-corrected aug-cc-pVDZ basis set.

	Cx_Cx PD		correlation				
System	Rings	HF	MP2	LNO-CCSD(T)	CCSD	CCSD(T)	CCSD(cT-fit)
				vvTight			
c_c	1	16.20	-33.59	-25.94	-21.53	-25.90	-25.14
c2_c2	2	30.84	-73.71	-54.35	-44.57	-54.19	-52.35
c3_c3	3	45.23	-116.26	-83.80	-68.16	-83.35	-80.30
c4_c4	4	59.57	-160.03	-113.55	-92.08	-112.98	-108.68
c5_c5	5	74.40					
c6_c6	6	88.92					

Table S149: Interaction energies (in kJ/mol) for different methods for the acene PD series using a cp-corrected aug-cc-pVTZ basis set.

	Cx_Cx PD		correlation				
System	Rings	HF	MP2	LNO-CCSD(T)	CCSD	CCSD(T)	CCSD(cT-fit)
				vvTight			
c_c	1	16.12	-34.94	-27.11	-22.11	-26.98	-26.12
c2_c2	2	30.72	-76.15	-56.47	-45.57	-56.09	-54.05
c3_c3	3	45.05					
c4_c4	4	59.31					
c5_c5	5	74.09					
c6_c6	6	88.54					

Table S150: Interaction energies (in kJ/mol) for different methods for the acene PD series using a cp-corrected aug-cc-pVQZ basis set.

	Cx_Cx PD		correlation				
System	Rings	HF	MP2	LNO-CCSD(T)	CCSD	CCSD(T)	CCSD(cT-fit)
				vvTight			
c_c	1	16.11	-35.38	-27.51	-22.29	-27.28	-26.38
c2_c2	2	30.71		-57.33			
c3_c3	3	45.04					
c4_c4	4	59.29					
c5_c5	5	74.07					
c6_c6	6	88.52					

Table S151: Interaction energies (in kJ/mol) for different methods for the acene PD series using a cp-corrected aug-cc-pV5Z basis set.

	Cx_Cx PD		correlation				
System	Rings	HF	MP2	LNO-CCSD(T)	CCSD	CCSD(T)	CCSD(cT-fit)
				vvTight			
c_c	1	16.11	-35.54	-27.51	-22.34	-27.36	-26.46
c2_c2	2	30.70		-57.15			
c3_c3	3	45.03					
c4_c4	4	59.28					
c5_c5	5	74.05					
c6_c6	6	88.50					

Table S152: Interaction energies (in kJ/mol) for different methods for the BNx_NBx series using a cp-corrected cc-pVDZ basis set.

	BNx_NBx		correlation								
System	Rings	HF	MP2	MP3	MP2.5	dRPA	LNO-CCSD(T)	DFT-SAPT	CCSD	CCSD(T)	CCSD(cT-fit)
							vvTight	disp			
bn_nb	1	11.46	-13.56	-10.84	-12.20	-13.96	-13.12	-14.13	-11.00	-13.04	-12.82
bn2_nb2	2	21.66	-27.86	-20.92	-24.39	-27.55	-26.01	-28.16	-21.84	-26.01	-25.52
bn3_nb3	3	33.17	-43.06	-31.34	-37.20	-41.75	-39.59	-42.82	-33.19	-39.60	-38.83
bn4_nb4	4	45.20	-58.65	-41.99	-50.32	-56.34	-54.12	-57.87	-44.81	-53.53	-52.45
bn5_nb5	5	52.22	-71.11	-50.42	-60.77	-67.66	-65.49	-69.83	-54.04	-64.58	-63.27
bn6_nb6	6	64.94	-87.11	-61.38	-74.25	-82.69	-79.01	-85.38	-65.97	-78.88	-77.26

Table S153: Interaction energies (in kJ/mol) for post-CCSD(T) methods for the BNx_NBx series using a cp-corrected cc-pVDZ basis set.

DZ	BNx_NBx	correlation				
System	Rings	CCSD(T)-L	CCSDT-2	CCSDT-3	CCSDT	CCSDT(Q)
bn_nb	1	-12.96	-12.80	-12.88	-12.90	-12.98
bn2_nb2	2	-25.87	-25.51	-25.68		
bn3_nb3	3	-39.41				
bn4_nb4	4	-53.27				

Table S154: Interaction energies (in kJ/mol) for different methods for the BNx_NBx series using a cp-corrected cc-pVTZ basis set.

	BNx_NBx		correlation								
System	Rings	HF	MP2	MP3	MP2.5	dRPA	LNO-CCSD(T)	DFT-SAPT	CCSD	CCSD(T)	CCSD(cT-fit)
							vvTight	disp			
bn_nb	1	11.73	-17.75	-14.14	-15.95	-17.60	-16.91	-18.34	-14.10	-17.09	-16.76
bn2_nb2	2	22.01	-35.36	-26.55	-30.96	-33.96	-32.95	-35.39	-27.25	-33.14	-32.43
bn3_nb3	3	33.49	-53.95	-39.36	-46.66	-50.99	-49.66	-53.12	-40.96	-49.89	-48.78
bn4_nb4	4	45.48	-73.02	-52.47	-62.75	-68.40	-66.83	-71.28	-55.01	-67.05	-65.52
bn5_nb5	5	52.45				-81.83		-85.49			
bn6_nb6	6	65.11				-99.86		-104.26			

Table S155: Interaction energies (in kJ/mol) for different methods for the BNx_NBx series using a cp-corrected cc-pVQZ basis set.

	BNx_NBx		correlation								
System	Rings	HF	MP2	MP3	MP2.5	dRPA	LNO-CCSD(T)	DFT-SAPT	CCSD	CCSD(T)	CCSD(cT-fit)
							vvTight	disp			
bn_nb	1	11.77	-19.07	-15.40	-17.23	-19.05	-18.43	-19.91	-15.20	-18.57	-18.20
bn2_nb2	2	22.09	-37.58	-28.63	-33.10	-36.35	-35.28	-37.95	-29.04	-35.59	-34.80
bn3_nb3	3	33.58				-54.31	-52.87	-56.65			
bn4_nb4	4	45.56				-72.74		-75.81			
bn5_nb5	5	52.49				-86.89		-90.76			
bn6_nb6	6	65.13				-105.95					

Table S156: Interaction energies (in kJ/mol) for different methods for the BNx_NBx series using a cp-corrected cc-pV5Z basis set.

	BNx_NBx		correlation					
System	Rings	HF	MP2	LNO-CCSD(T)	DFT-SAPT	CCSD	CCSD(T)	CCSD(cT-fit)
				vvTight	disp			
bn_nb	1	11.81	-19.54	-18.95	-20.52	-15.59	-19.11	-18.72
bn2_nb2	2	22.12			-38.88			
bn3_nb3	3	33.60			-57.92			
bn4_nb4	4	45.57						
bn5_nb5	5	52.47						
bn6_nb6	6	65.10						

Table S157: Interaction energies (in kJ/mol) for different methods for the BNx_NBx series using a cp-corrected aug-cc-pVDZ basis set.

	BNx_NBx		correlation				
System	Rings	HF	MP2	LNO-CCSD(T)	CCSD	CCSD(T)	CCSD(cT-fit)
				vvTight			
bn_nb	1	11.93	-18.25	-17.95	-14.98	-18.05	-17.73
bn2_nb2	2	22.14	-36.12	-34.34	-28.67	-34.68	-34.00
bn3_nb3	3	33.54	-54.99	-51.49	-42.94	-52.02	-50.96
bn4_nb4	4	45.43		-68.94			
bn5_nb5	5	52.19					
bn6_nb6	6	64.94					

Table S158: Interaction energies (in kJ/mol) for different methods for the BNx_NBx series using a cp-corrected aug-cc-pVTZ basis set.

	BNx_NBx PD		correlation					
System	Rings	HF	MP2	LNO-CCSD(T)	CCSD	CCSD(T)	CCSD(cT-fit)
				vvTight			
bn_nb	1	11.81	-19.41	-18.98	-15.68	-19.17	-18.79
bn2_nb2	2	22.11	-38.06	-36.15	-29.79	-36.49	-35.71
bn3_nb3	3	33.59		-54.06			
bn4_nb4	4	45.55					
bn5_nb5	5	52.45					
bn6_nb6	6	65.07					

Table S159: Interaction energies (in kJ/mol) for different methods for the BNx_NBx series using a cp-corrected aug-cc-pVQZ basis set.

	BNx_NBx		correlation				
System	Rings	HF	MP2	LNO-CCSD(T)	CCSD	CCSD(T)	CCSD(cT-fit)
				vvTight			
bn_nb	1	11.81	-19.67	-19.25	-15.76	-19.32	-18.93
bn2_nb2	2	22.13					
bn3_nb3	3	33.61					
bn4_nb4	4	45.58					
bn5_nb5	5	52.48					
bn6_nb6	6	65.11					

Table S160: Interaction energies (in kJ/mol) for different methods for the BNx_BNx series using a cp-corrected cc-pVDZ basis set.

	BNx_BNx		correlation								
System	Rings	HF	MP2	MP3	MP2.5	dRPA	LNO-CCSD(T)	DFT-SAPT	CCSD	CCSD(T)	CCSD(cT-fit)
							vvTight	disp			
bn_bn	1	13.99	-17.56	-13.95	-15.76	-19.98	-16.58	-23.58	-13.97	-16.71	-16.40
bn2_bn2	2	26.51	-39.49	-29.15	-34.32	-41.99	-36.11	-47.99	-30.33	-36.40	-35.66
bn4_bn3	3	38.24	-62.75	-44.83	-53.79	-65.76	-56.86	-72.74	-47.56	-57.18	-55.97
bn4_bn4	4	49.55	-86.17	-60.57	-73.37	-89.77	-77.61	-97.37	-64.87	-78.07	-76.39
bn5_bn5	5	60.78	-109.63	-76.37	-93.00	-113.75	-98.71	-122.04	-82.21	-99.00	-96.85
bn6_bn6	6	72.07	-133.18	-92.23	-112.71	-137.78	-119.64	-146.82	-99.62	-120.01	-117.38

Table S161: Interaction energies (in kJ/mol) for post-CCSD(T) methods for the BNx_BNx series using a cp-corrected cc-pVDZ basis set.

DZ	BNx_BNx	correlation				
System	Rings	CCSD(T)-L	CCSDT-2	CCSDT-3	CCSDT	CCSDT(Q)
bn_bn	1	-16.60	-16.36	-16.49	-16.48	-16.65
bn2_bn2	2	-36.08	-35.51	-35.79		
bn3_bn3	3	-56.71				
bn4_bn4	4	-77.45				

Table S162: Interaction energies (in kJ/mol) for different methods for the BNx_BNx series using a cp-corrected cc-pVTZ basis set.

	BNx_BNx		correlation								
System	Rings	HF	MP2	MP3	MP2.5	dRPA	LNO-CCSD(T)	DFT-SAPT	CCSD	CCSD(T)	CCSD(cT-fit)
							vvTight	disp			
bn_bn	1	12.57	-24.48	-19.53	-22.01	-25.21	-22.99	-30.15	-19.29	-23.32	-22.86
bn2_bn2	2	24.31	-51.96	-38.78	-45.37	-51.35	-47.63	-59.68	-39.64	-48.12	-47.07
bn4_bn3	3	35.43	-80.62	-58.37	-69.49	-78.90	-73.41	-89.44	-60.68	-73.82	-72.15
bn4_bn4	4	46.22	-109.33	-77.93	-93.63	-106.52	-99.11	-119.04	-81.71	-99.52	-97.22
bn5_bn5	5	56.95				-134.18		-148.71			
bn6_bn6	6	67.75				-161.92		-178.51			

Table S163: Interaction energies (in kJ/mol) for different methods for the BNx_BNx series using a cp-corrected cc-pVQZ basis set.

	BNx_BNx		correlation								
System	Rings	HF	MP2	MP3	MP2.5	dRPA	LNO-CCSD(T)	DFT-SAPT	CCSD	CCSD(T)	CCSD(cT-fit)
							vvTight	disp			
bn_bn	1	12.31	-26.55	-21.47	-24.01	-27.35	-24.50	-32.34	-21.01	-25.55	-25.04
bn2_bn2	2	24.01	-55.55	-42.13	-48.84	-55.13	-51.47	-63.52	-42.60	-51.98	-50.84
bn4_bn3	3	35.10				-84.20	-78.51	-94.86			
bn4_bn4	4	45.87				-113.37		-126.03			
bn5_bn5	5	56.58				-142.55		-157.28			
bn6_bn6	6	67.36				-171.82					

Table S164: Interaction energies (in kJ/mol) for different methods for the BNx_BNx series using a cp-corrected cc-pV5Z basis set.

	BNx_BNx		correlation								
System	Rings	HF	MP2	MP3	MP2.5	dRPA	LNO-CCSD(T)	DFT-SAPT	CCSD	CCSD(T)	CCSD(cT-fit)
							vvTight	disp			
bn_bn	1	12.30	-27.35				-26.25	-33.26	-21.68	-26.42	-25.88
bn2_bn2	2	24.02						-65.03			
bn4_bn3	3	35.13									
bn4_bn4	4	45.90									
bn5_bn5	5	56.63									
bn6_bn6	6	67.43									

Table S165: Interaction energies (in kJ/mol) for different methods for the BNx_BNx series using a cp-corrected aug-cc-pVDZ basis set.

	BNx_BNx		correlation				
System	Rings	HF	MP2	LNO-CCSD(T)	CCSD	CCSD(T)	CCSD(cT-fit)
				vvTight			
bn_bn	1	12.69	-24.83	-23.76	-19.95	-24.03	-23.59
bn2_bn2	2	24.68	-52.21	-48.66	-40.62	-49.09	-48.09
bn4_bn3	3	36.04	-80.83	-74.48	-62.08	-75.15	-73.56
bn4_bn4	4	47.05		-100.10			
bn5_bn5	5	58.00					
bn6_bn6	6	69.02					

Table S166: Interaction energies (in kJ/mol) for different methods for the BNx_BNx series using a cp-corrected aug-cc-pVTZ basis set.

	BNx_BNx		correlation				
System	Rings	HF	MP2	LNO-CCSD(T)	CCSD	CCSD(T)	CCSD(cT-fit)
				vvTight			
bn_bn	1	12.37	-26.99	-25.98	-21.56	-26.24	-25.72
bn2_bn2	2	24.14	-56.10	-52.48	-43.42	-52.98	-51.84
bn4_bn3	3	35.28		-79.82			
bn4_bn4	4	46.08					
bn5_bn5	5	56.84					
bn6_bn6	6	67.66					

Table S167: Interaction energies (in kJ/mol) for different methods for the BNx_BNx series using a cp-corrected aug-cc-pVQZ basis set.

	BNx_BNx		correlation				
System	Rings	HF	MP2	LNO-CCSD(T)	CCSD	CCSD(T)	CCSD(cT-fit)
				vvTight			
bn_bn	1	12.29	-27.52	-26.48	-21.88	-26.67	-26.13
bn2_bn2	2	24.02					
bn4_bn3	3	35.12					
bn4_bn4	4	45.89					
bn5_bn5	5	56.62					
bn6_bn6	6	67.41					

Table S168: Interaction energies (in kJ/mol) for different methods for the acene PD series using cp-uncorrected cc-pVDZ basis set.

	Cx_Cx PD		correlation							
System	Rings	HF	MP2	MP3	MP2.5	dRPA	LNO-CCSD(T)	CCSD	CCSD(T)	CCSD(cT-fit)
							vvTight			
c_c	1	11.89	-28.24	-18.42	-23.33	-21.51	-21.85	-18.19	-21.65	-21.06
c2_c2	2	23.09	-64.41	-37.13	-50.77	-47.56	-47.86	-39.35	-47.30	-45.81
c3_c3	3	33.81	-102.92	-54.85	-78.88	-74.64	-74.70	-61.14	-73.88	-71.38
c4_c4	4	44.21	-142.74	-72.27	-107.51	-102.20	-101.92	-83.38	-101.06	-97.49
c5_c5	5	55.05	-183.62	-89.25	-136.44	-130.71	-129.90	-106.02	-128.78	-124.12
c6_c6	6	65.56	-224.10	-105.44	-164.77	-158.98	-157.54	-128.36	-156.20	-150.42

Table S169: Interaction energies (in kJ/mol) for post-CCSD(T) methods for the acene PD series using cp-uncorrected cc-pVDZ basis set.

DZ	Cx_Cx PD	correlation				
System	Rings	CCSD(T)-L	CCSDT-2	CCSDT-3	CCSDT	CCSDT(Q)
c_c	1	-21.54	-21.24	-21.25	-21.02	-21.45
c2_c2	2	-46.87	-46.09	-46.16		
c3_c3	3	-73.22				
c4_c4	4	-99.93				

Table S170: Interaction energies (in kJ/mol) for different methods for the acene PD series using cp-uncorrected cc-pVTZ basis set.

	Cx_Cx PD		correlation							
System	Rings	HF	MP2	MP3	MP2.5	dRPA	LNO-CCSD(T)	CCSD	CCSD(T)	CCSD(cT-fit)
							vvTight			
c_c	1	14.36	-34.84	-22.45	-28.64	-26.71	-26.92	-22.10	-26.78	-25.96
c2_c2	2	28.04	-77.52	-44.01	-60.77	-57.65	-57.68	-46.95	-57.31	-55.34
c3_c3	3	41.45	-122.81	-64.50	-93.66	-89.81	-89.45	-72.52	-88.89	-85.65
c4_c4	4	54.74	-169.31	-84.36	-126.84	-122.53	-121.73	-98.43	-120.95	-116.38
c5_c5	5	68.55				-156.22				
c6_c6	6	82.02				-189.66				

Table S171: Interaction energies (in kJ/mol) for different methods for the acene PD series using cp-uncorrected cc-pVQZ basis set.

	Cx_Cx PD		correlation							
System	Rings	HF	MP2	MP3	MP2.5	dRPA	LNO-CCSD(T)	CCSD	CCSD(T)	CCSD(cT-fit)
							vvTight			
c_c	1	15.39	-35.68	-22.80	-29.24	-26.58	-27.42	-22.33	-27.28	-26.38
c2_c2	2	29.63	-78.27	-43.68	-60.97	-56.25	-57.71	-46.58	-57.34	-55.23
c3_c3	3	43.62				-86.97				
c4_c4	4	57.53				-118.25				
c5_c5	5	71.95				-150.37				
c6_c6	6	86.04								

Table S172: Interaction energies (in kJ/mol) for different methods for the acene PD series using cp-uncorrected cc-pV5Z basis set.

	Cx_Cx PD		correlation							
System	Rings	HF	MP2	MP3	MP2.5	dRPA	LNO-CCSD(T)	CCSD	CCSD(T)	CCSD(cT-fit)
							vvTight			
c_c	1	15.95	-35.96			-26.46	-27.69	-22.53	-27.56	-26.65
c2_c2	2	30.48					-57.81			
c3_c3	3	44.74								
c4_c4	4	58.93								
c5_c5	5	73.65								
c6_c6	6	88.03								

Table S173: Interaction energies (in kJ/mol) for different methods for the acene PD series using cp-uncorrected aug-cc-pVDZ basis set.

	Cx_Cx PD		correlation				
System	Rings	HF	MP2	LNO-CCSD(T)	CCSD	CCSD(T)	CCSD(cT-fit)
				vvTight			
c_c	1	10.62	-42.35	-35.41	-29.52	-35.30	-34.44
c2_c2	2	18.82	-94.04	-76.12	-63.00	-75.86	-73.79
c3_c3	3	26.41	-148.94	-118.61	-97.66	-118.05	-114.65
c4_c4	4	34.13	-205.08	-161.57	-132.66	-160.75	-155.95
c5_c5	5	42.22					
c6_c6	6	50.00					

Table S174: Interaction energies (in kJ/mol) for different methods for the acene PD series using cp-uncorrected aug-cc-pVTZ basis set.

	Cx_Cx PD		correlation				
System	Rings	HF	MP2	LNO-CCSD(T)	CCSD	CCSD(T)	CCSD(cT-fit)
				vvTight			
c_c	1	15.18	-39.86	-31.66	-26.28	-31.53	-30.66
c2_c2	2	28.85	-87.25	-66.64	-54.90	-66.30	-64.26
c3_c3	3	42.29					
c4_c4	4	55.67					
c5_c5	5	69.57					
c6_c6	6	83.15					

Table S175: Interaction energies (in kJ/mol) for different methods for the acene PD series using cp-uncorrected aug-cc-pVQZ basis set.

	Cx_Cx PD		correlation				
System	Rings	HF	MP2	LNO-CCSD(T)	CCSD	CCSD(T)	CCSD(cT-fit)
				vvTight			
c_c	1	15.86	-37.33	-28.85	-23.57	-28.70	-27.79
c2_c2	2	30.25		-60.04			
c3_c3	3	44.40					
c4_c4	4	58.48					
c5_c5	5	73.08					
c6_c6	6	87.35					

Table S176: Interaction energies (in kJ/mol) for different methods for the acene PD series using cp-uncorrected aug-cc-pV5Z basis set.

	Cx_Cx PD		correlation				
System	Rings	HF	MP2	LNO-CCSD(T)	CCSD	CCSD(T)	CCSD(cT-fit)
				vvTight			
c_c	1	16.07	-36.42	-28.02	-22.83	-27.91	-26.99
c2_c2	2	30.65		-58.23			
c3_c3	3	44.95					
c4_c4	4	59.18					
c5_c5	5	73.93					
c6_c6	6	88.35					

Table S177: Interaction energies (in kJ/mol) for different methods for the BNx_NBx series using cp-uncorrected cc-pVDZ basis set.

	BNx_NBx		correlation							
System	Rings	HF	MP2	MP3	MP2.5	dRPA	LNO-CCSD(T)	CCSD	CCSD(T)	CCSD(cT-fit)
							vvTight			
bn_nb	1	6.61	-18.63	-15.60	-17.12	-21.60	-18.14	-15.59	-18.34	-18.07
bn2_nb2	2	12.59	-38.80	-31.19	-35.00	-43.84	-37.10	-31.76	-37.54	-36.94
bn3_nb3	3	19.94	-60.21	-47.45	-53.83	-67.28	-57.26	-48.78	-57.77	-56.81
bn4_nb4	4	27.87	-82.15	-64.07	-73.11	-91.40	-77.89	-66.20	-78.47	-77.14
bn5_nb5	5	31.98	-99.68	-77.34	-88.51	-110.62	-94.35	-80.14	-95.00	-93.39
bn6_nb6	6	40.61	-122.16	-94.42	-108.29	-135.45	-115.41	-98.01	-116.24	-114.25

Table S178: Interaction energies (in kJ/mol) for post-CCSD(T) methods for the BNx_NBx series using cp-uncorrected cc-pVDZ basis set.

DZ	BNx_NBx	correlation				
System	Rings	CCSD(T)-L	CCSDT-2	CCSDT-3	CCSDT	CCSDT(Q)
bn_nb	1	-18.24	-18.07	-18.15	-18.20	-18.29
bn2_nb2	2	-37.34	-36.95	-37.13		
bn3_nb3	3	-57.48				
bn4_nb4	4	-78.09				

Table S179: Interaction energies (in kJ/mol) for different methods for the BNx_NBx series using cp-uncorrected cc-pVTZ basis set.

	BNx_NBx		correlation							
System	Rings	HF	MP2	MP3	MP2.5	dRPA	LNO-CCSD(T)	CCSD	CCSD(T)	CCSD(cT-fit)
							vvTight			
bn_nb	1	9.50	-20.25	-16.17	-18.21	-21.24	-19.21	-15.99	-19.31	-18.93
bn2_nb2	2	18.29	-40.78	-31.09	-35.94	-42.12	-37.95	-31.54	-38.16	-37.37
bn3_nb3	3	28.40	-62.38	-46.53	-54.45	-63.85	-57.47	-47.77	-57.84	-56.61
bn4_nb4	4	39.05	-84.51	-62.33	-73.42	-86.23	-77.44	-64.39	-77.99	-76.30
bn5_nb5	5	44.98				-103.77				
bn6_nb6	6	56.31				-126.84				

Table S180: Interaction energies (in kJ/mol) for different methods for the BNx_NBx series using cp-uncorrected cc-pVQZ basis set.

	BNx_NBx		correlation							
System	Rings	HF	MP2	MP3	MP2.5	dRPA	LNO-CCSD(T)	CCSD	CCSD(T)	CCSD(cT-fit)
							vvTight			
bn_nb	1	10.84	-20.43	-16.31	-18.37	-21.09	-19.49	-16.06	-19.60	-19.19
bn2_nb2	2	20.60	-40.34	-30.54	-35.44	-41.07	-37.47	-30.88	-37.75	-36.91
bn3_nb3	3	31.59				-61.15	-56.25			
bn4_nb4	4	43.08				-82.09				
bn5_nb5	5	49.65				-98.27				
bn6_nb6	6	61.81				-119.88				

Table S181: Interaction energies (in kJ/mol) for different methods for the BNx_NBx series using cp-uncorrected cc-pV5Z basis set.

	BNx_NBx		correlation				
System	Rings	HF	MP2	LNO-CCSD(T)	CCSD	CCSD(T)	CCSD(cT-fit)
				vvTight			
bn_nb	1	11.66	-20.25	-19.47	-16.02	-19.60	-19.20
bn2_nb2	2	21.88					
bn3_nb3	3	33.27					
bn4_nb4	4	45.16					
bn5_nb5	5	51.98					
bn6_nb6	6	64.52					

Table S182: Interaction energies (in kJ/mol) for different methods for the BNx_NBx series using cp-uncorrected aug-cc-pVDZ basis set.

	BNx_NBx		correlation				
System	Rings	HF	MP2	LNO-CCSD(T)	CCSD	CCSD(T)	CCSD(cT-fit)
				vvTight			
bn_nb	1	7.40	-26.85	-27.10	-22.96	-27.20	-26.81
bn2_nb2	2	12.16	-55.38	-54.92	-46.62	-55.41	-54.56
bn3_nb3	3	18.01	-85.66	-84.56	-71.57	-85.10	-83.76
bn4_nb4	4	24.26		-115.01			
bn5_nb5	5	26.15					
bn6_nb6	6	40.61					

Table S183: **Interaction energies (in kJ/mol) for different methods for the BNx_NBx series using cp-uncorrected aug-cc-pVTZ basis set.**

	BNx_NBx		correlation				
System	Rings	HF	MP2	LNO-CCSD(T)	CCSD	CCSD(T)	CCSD(cT-fit)
				vvTight			
bn_nb	1	10.97	-23.55	-22.85	-19.24	-23.02	-22.62
bn2_nb2	2	20.32	-46.90	-44.45	-37.42	-44.74	-43.92
bn3_nb3	3	30.77		-67.48			
bn4_nb4	4	41.79					
bn5_nb5	5	47.80					
bn6_nb6	6	59.43					

Table S184: Interaction energies (in kJ/mol) for different methods for the BNx_NBx series using cp-uncorrected aug-cc-pVQZ basis set.

	BNx_NBx		correlation				
System	Rings	HF	MP2	LNO-CCSD(T)	CCSD	CCSD(T)	CCSD(cT-fit)
				vvTight			
bn_nb	1	11.57	-21.24	-19.60	-16.81	-20.47	-20.06
bn2_nb2	2	21.69					
bn3_nb3	3	33.02					
bn4_nb4	4	44.86					
bn5_nb5	5	51.63					
bn6_nb6	6	64.12					

Table S185: Interaction energies (in kJ/mol) for different methods for the BNx_BNx series using cp-uncorrected cc-pVDZ basis set.

	BNx_BNx		correlation							
System	Rings	HF	MP2	MP3	MP2.5	dRPA	LNO-CCSD(T)	CCSD	CCSD(T)	CCSD(cT-fit)
							vvTight			
bn_bn	1	3.17	-27.00	-22.39	-24.69	-31.54	-25.76	-21.80	-25.82	-25.38
bn2_bn2	2	8.24	-59.79	-47.58	-53.68	-68.08	-56.29	-47.70	-56.72	-55.72
bn4_bn3	3	13.01	-94.14	-73.50	-83.82	-107.18	-88.44	-74.78	-89.11	-87.50
bn4_bn4	4	17.52	-128.61	-99.44	-114.02	-146.51	-120.59	-101.89	-121.54	-119.32
bn5_bn5	5	21.97	-163.09	-125.42	-144.25	-185.77	-152.93	-129.02	-153.98	-151.15
bn6_bn6	6	26.48	-197.68	-151.49	-174.58	-225.13	-185.23	-156.23	-186.53	-183.08

Table S186: Interaction energies (in kJ/mol) for post-CCSD(T) methods for the BNx_BNx series using cp-uncorrected cc-pVDZ basis set.

DZ	BNx_BNx	correlation				
System	Rings	CCSD(T)-L	CCSDT-2	CCSDT-3	CCSDT	CCSDT(Q)
bn_bn	1	-25.68	-25.41	-25.53	-25.55	-25.70
bn2_bn2	2	-56.33	-55.67	-55.94		
bn3_bn3	3	-88.50				
bn4_bn4	4	-120.73				

Table S187: Interaction energies (in kJ/mol) for different methods for the BNx_BNx series using cp-uncorrected cc-pVTZ basis set.

	BNx_BNx		correlation							
System	Rings	HF	MP2	MP3	MP2.5	dRPA	LNO-CCSD(T)	CCSD	CCSD(T)	CCSD(cT-fit)
							vvTight			
bn_bn	1	8.77	-29.52	-23.51	-26.51	-31.83	-27.47	-22.99	-27.72	-27.16
bn2_bn2	2	18.17	-61.94	-46.93	-54.43	-65.38	-56.75	-47.35	-57.24	-56.02
bn4_bn3	3	27.19	-95.63	-70.84	-83.24	-100.70	-87.15	-72.56	-87.82	-85.92
bn4_bn4	4	35.98	-129.31	-94.69	-112.00	-136.21	-117.42	-97.71	-118.36	-115.76
bn5_bn5	5	44.75				-171.70				
bn6_bn6	6	53.56				-207.29				

Table S188: Interaction energies (in kJ/mol) for different methods for the BNx_BNx series using cp-uncorrected cc-pVQZ basis set.

	BNx_BNx		correlation							
System	Rings	HF	MP2	MP3	MP2.5	dRPA	LNO-CCSD(T)	CCSD	CCSD(T)	CCSD(cT-fit)
							vvTight			
bn_bn	1	10.75	-28.87	-22.97	-25.92	-30.54	-27.05	-22.45	-27.28	-26.71
bn2_bn2	2	21.60	-60.11	-45.24	-52.68	-62.45	-55.06	-45.61	-55.56	-54.32
bn4_bn3	3	31.92				-95.13	-84.04			
bn4_bn4	4	41.95				-128.22				
bn5_bn5	5	51.93				-161.30				
bn6_bn6	6	61.98				-194.47				

Table S189: Interaction energies (in kJ/mol) for different methods for the BNx_BNx series using cp-uncorrected cc-pV5Z basis set.

	BNx_BNx		correlation				
System	Rings	HF	MP2	LNO-CCSD(T)	CCSD	CCSD(T)	CCSD(cT-fit)
				vvTight			
bn_bn	1	12.06	-28.42	-26.94	-22.30	-27.15	-26.59
bn2_bn2	2	23.68					
bn4_bn3	3	34.68					
bn4_bn4	4	45.36					
bn5_bn5	5	55.99					
bn6_bn6	6	66.68					

Table S190: Interaction energies (in kJ/mol) for different methods for the BNx_BNx series using cp-uncorrected aug-cc-pVDZ basis set.

	BNx_BNx		correlation				
System	Rings	HF	MP2	LNO-CCSD(T)	CCSD	CCSD(T)	CCSD(cT-fit)
				vvTight			
bn_bn	1	7.37	-36.01	-35.62	-30.29	-35.87	-35.33
bn2_bn2	2	12.59	-77.25	-75.38	-63.91	-75.95	-74.73
bn4_bn3	3	17.19	-120.55	-117.03	-99.06	-117.83	-115.90
bn4_bn4	4	21.46		-159.04			
bn5_bn5	5	25.73					
bn6_bn6	6	30.09					

Table S191: Interaction energies (in kJ/mol) for different methods for the BNx_BNx series using cp-uncorrected aug-cc-pVTZ basis set.

	BNx_BNx		correlation				
System	Rings	HF	MP2	LNO-CCSD(T)	CCSD	CCSD(T)	CCSD(cT-fit)
				vvTight			
bn_bn	1	11.30	-32.69	-31.28	-26.45	-31.53	-30.99
bn2_bn2	2	21.83	-68.65	-64.27	-54.25	-64.68	-63.49
bn4_bn3	3	31.76		-98.56			
bn4_bn4	4	41.40					
bn5_bn5	5	51.01					
bn6_bn6	6	60.68					

Table S192: Interaction energies (in kJ/mol) for different methods for the BNx_BNx series using cp-uncorrected aug-cc-pVQZ basis set.

	BNx_BNx		correlation				
System	Rings	HF	MP2	LNO-CCSD(T)	CCSD	CCSD(T)	CCSD(cT-fit)
				vvTight			
bn_bn	1	12.00	-29.69	-28.07	-23.32	-28.25	-27.68
bn2_bn2	2	18.55					
bn4_bn3	3	34.43					
bn4_bn4	4	45.05					
bn5_bn5	5	55.61					
bn6_bn6	6	66.24					

Table S193: Slopes (in kJ/mol per ring) and Intercepts (in kJ/mol) for Figure 3.

Subfigure		Method	Slope	Intercept
a	DZ cp-corrected	MP2	-33.79	11.4
		MP3	-12.99	-3.5
		MP2.5	-23.39	4.0
		dRPA	-20.90	4.8
		DFT-SAPT	-22.31	6.1
		CCSD	-17.99	3.0
		CCSD(T)	-21.89	4.4
b	DZ cp-corrected	MP2	-14.66	1.1
		MP3	-10.05	-1.0
		MP2.5	-12.36	0.1
		dRPA	-13.67	-0.5
		DFT-SAPT	-14.18	-0.1
		CCSD	-10.94	-0.2
		CCSD(T)	-13.11	-0.1
c	DZ cp-corrected	MP2	-23.20	6.4
		MP3	-15.68	2.0
		MP2.5	-19.44	4.2
		dRPA	-23.67	4.7
		DFT-SAPT	-24.66	1.2
		CCSD	-17.18	3.7
		CCSD(T)	-20.72	4.6
d	DZ cp-uncorrected	MP2	-39.34	13.3
		MP3	-17.40	-2.0
		MP2.5	-28.37	5.7
		dRPA	-27.55	7.2
		CCSD	-22.09	4.6
		CCSD(T)	-26.98	6.3
e	DZ cp-uncorrected	MP2	-20.64	2.0
		MP3	-15.69	-0.1
		MP2.5	-18.16	0.9
		dRPA	-22.68	1.0
		CCSD	-16.42	0.7
		CCSD(T)	-19.50	1.0
f	DZ cp-uncorrected	MP2	-34.22	8.1
		MP3	-25.86	3.9
		MP2.5	-30.04	6.0
		dRPA	-38.87	8.7
		CCSD	-26.95	5.8
		CCSD(T)	-32.22	7.2

Assessment of the Results when extrapolating to Larger Molecules

Tables S194, S195, S196, S197, S198, S199 summarize extrapolated results for cp-corrected and cp-uncorrected slopes in CBS for acene PD, BNx_NBx and BNx_BNx systems.

Table S194: Best available CP-Corrected Correlation Energy Slopes (kJ/mol per Ring), including post-CCSD(T) Values, of the Acene PD Dimers[a]

		Method							
				CCSD(T)			post-CCSD(T)		
Slopes	Basis Set	MP2	CCSD	`Tight`	`VeryTight`	canonical	CCSDT-2	CCSDT-3	$CCSD(T)_\lambda$
1→2	DZ(d,s)	-29.99	-16.65			-20.11	-19.49	-19.54	-19.94
1→2	DZ	-30.88	-17.22	-21.17	-21.11	-20.76	-20.18	-20.23	-20.64
1→4	DZ	-32.78	-17.73	-22.69	-21.85	-21.50	*-20.93*	*-20.97*	-21.28
1→6	DZ	-33.79	-17.99	-23.08	-22.19	-21.89	*-21.32*	*-21.36*	*-21.68*
1→2	TZ	-38.61	-21.65	-26.97	-27.08	-26.66	*-25.86*[b]	*-25.93*[b]	*-26.49*[b]
1→4	TZ	-40.68	-22.15	-28.72	-27.95	-27.43	*-26.63*[b]	*-26.69*[b]	*-27.13*[b]
1→6	TZ	*-41.69*	*-22.41*	-29.50	-28.37	*-27.82*	*-27.02*[b]	*-27.08*[b]	*-27.52*[b]
1→2	{T,Q}Z	-42.28	-23.98	-31.88	-30.18	-29.91	*-29.10*[b]	*-29.17*[b]	*-29.74*[b]
1→4	{T,Q}Z	*-44.35*	*-24.49*	-31.99	-31.01	*-30.67*	*-29.87*[b]	*-29.93*[b]	*-30.37*[b]
1→6	{T,Q}Z	*-45.63*	*-24.83*	-32.40	-31.72	*-31.40*	*-30.60*[b]	*-30.66*[b]	*-31.10*[b]
1→2	{Q,5}Z	-42.20	*-24.07*	-31.47	-30.41	*-30.07*	*-29.27*[b]	*-29.33*[b]	*-29.90*[b]
1→4	{Q,5}Z	*-44.23*	*-24.55*	-32.01	-31.16	*-30.92*	*-30.12*[b]	*-30.18*[b]	*-30.62*[b]
1→6	{Q,5}Z	*-45.40*	*-24.86*	-32.45	-31.75	*-31.51*	*-30.71*[b]	*-30.77*[b]	*-31.21*[b]

[a] Numbers in italics are estimates. [b] The post-CCSD(T) slopes have been scaled by a factor of 1.4 to account for the small cc-pVDZ basis set size — see discussion in[S1] and in its Supporting Information.

Table S195: Best available Correlation Energy Slopes (kJ/mol per Ring), including post-CCSD(T) Values, of the Acene PD Dimers[a]

		Method							
				CCSD(T)			post-CCSD(T)		
Slopes	Basis Set	MP2	CCSD	`Tight`	`VeryTight`	canonical	CCSDT-2	CCSDT-3	$CCSD(T)_\lambda$
1→2	DZ(d,s)	-35.22	-20.53			-24.97	-24.25	-24.30	-24.73
1→2	DZ	-36.17	-21.16	-26.68	-26.16	-25.65	-24.85	-24.90	-25.33
1→4	DZ	-38.20	-21.73	-27.78	-26.85	-26.48	*-25.68*	*-25.74*	-26.15
1→6	DZ	-39.34	-22.09	-28.19	-27.32	-26.98	*-26.19*	*-26.24*	*-26.65*
1→2	TZ	-42.68	-24.85	-31.52	-30.90	-30.53	*-29.42*[b]	*-29.49*[b]	*-30.10*[b]
1→4	TZ	-44.87	-25.45	-32.46	-31.81	-31.41	*-30.30*[b]	*-30.37*[b]	*-30.95*[b]
1→6	TZ	*-46.00*	*-25.81*	-33.12	-32.38	*-31.91*	*-30.80*[b]	*-30.87*[b]	*-31.46*[b]
1→2	{T,Q}Z	-42.53	-23.81	-31.01	-30.19	-29.72	*-28.61*[b]	*-28.68*[b]	*-29.29*[b]
1→4	{T,Q}Z	*-44.72*	*-24.42*	-31.61	-30.96	*-30.60*	*-29.49*[b]	*-29.56*[b]	*-30.14*[b]
1→6	{T,Q}Z	*-45.91*	*-24.74*	-32.20	-31.59	*-31.28*	*-30.17*[b]	*-30.24*[b]	*-30.82*[b]
1→2	{Q,5}Z	-42.30	*-23.80*	-30.88	-30.24	*-29.81*	*-28.70*[b]	*-28.77*[b]	*-29.38*[b]
1→4	{Q,5}Z	*-44.17*	*-24.16*	-32.20	-30.96	*-30.48*	*-29.37*[b]	*-29.44*[b]	*-30.03*[b]
1→6	{Q,5}Z	*-45.34*	*-24.49*	-32.44	-31.57	*-31.09*	*-29.98*[b]	*-30.05*[b]	*-30.63*[b]

[a] Numbers in italics are estimates. [b] The post-CCSD(T) slopes have been scaled by a factor of 1.4 to account for the small cc-pVDZ basis set size — see discussion in[S1] and in its Supporting Information.

Equations for estimation slopes for acene PD series are as follows:

$$E^{\mathrm{canonical}}_{\mathrm{cc-pVTZ}}(1 \rightarrow 6) \approx E^{\mathrm{canonical}}_{\mathrm{cc-pVTZ}}(1 \rightarrow 4) + E^{\mathrm{canonical}}_{\mathrm{cc-pVDZ}}(1 \rightarrow 6) - E^{\mathrm{canonical}}_{\mathrm{cc-pVDZ}}(1 \rightarrow 4) \tag{1}$$

$$E^{\mathrm{canonical}}_{\mathrm{cc-pVQZ}}(1 \rightarrow 4) \approx E^{\mathrm{canonical}}_{\mathrm{cc-pVQZ}}(1 \rightarrow 2) + E^{\mathrm{canonical}}_{\mathrm{cc-pVTZ}}(1 \rightarrow 4) - E^{\mathrm{canonical}}_{\mathrm{cc-pVTZ}}(1 \rightarrow 2) \tag{2}$$

$$E^{\mathrm{canonical}}_{\mathrm{cc-pVQZ}}(1 \rightarrow 6) \approx E^{\mathrm{canonical}}_{\mathrm{cc-pVQZ}}(1 \rightarrow 4) + E^{\mathrm{LNO,vTight}}_{\mathrm{cc-pVQZ}}(1 \rightarrow 6) - E^{\mathrm{LNO,vTight}}_{\mathrm{cc-pVQZ}}(1 \rightarrow 4) \tag{3}$$

$$E^{\mathrm{canonical}}_{\mathrm{cc-pV5Z}}(1 \rightarrow 2) \approx E^{\mathrm{canonical}}_{\mathrm{cc-pVQZ}}(1 \rightarrow 2) + E^{\mathrm{LNO,vTight}}_{\mathrm{cc-pV5Z}}(1 \rightarrow 2) - E^{\mathrm{LNO,vTight}}_{\mathrm{cc-pVQZ}}(1 \rightarrow 2) \tag{4}$$

$$E^{\mathrm{canonical}}_{\mathrm{cc-pV5Z}}(1 \rightarrow 4) \approx E^{\mathrm{canonical}}_{\mathrm{cc-pV5Z}}(1 \rightarrow 2) + E^{\mathrm{LNO,vTight}}_{\mathrm{cc-pV5Z}}(1 \rightarrow 4) - E^{\mathrm{LNO,vTight}}_{\mathrm{cc-pV5Z}}(1 \rightarrow 2) \tag{5}$$

$$E^{\mathrm{LNO,vTight}}_{\mathrm{cc-pV5Z}}(1\to 6) \approx E^{\mathrm{LNO,vTight}}_{\mathrm{cc-pV5Z}}(1\to 4) + E^{\mathrm{LNO,vTight}}_{\mathrm{cc-pVQZ}}(1\to 6) - E^{\mathrm{LNO,vTight}}_{\mathrm{cc-pVQZ}}(1\to 4) \quad (6)$$

$$E^{\mathrm{canonical}}_{\mathrm{cc-pV5Z}}(1\to 6) \approx E^{\mathrm{canonical}}_{\mathrm{cc-pV5Z}}(1\to 4) + E^{\mathrm{LNO,vTight}}_{\mathrm{cc-pV5Z}}(1\to 6) - E^{\mathrm{LNO,vTight}}_{\mathrm{cc-pV5Z}}(1\to 4) \quad (7)$$

Table S196: Best available CP-Corrected Correlation Energy Slopes (kJ/mol per Ring), including post-CCSD(T) Values, of the BNx_NBx Dimers[a]

		Method							
				CCSD(T)			post-CCSD(T)		
Slopes	Basis Set	MP2	CCSD	`Tight`	`VeryTight`	canonical	CCSDT-2	CCSDT-3	CCSD(T)$_\lambda$
1→2	DZ(d,s)	-13.77	-10.50			-12.57	-12.33	-12.42	-12.53
1→2	DZ	-14.30	-10.83	-12.69	-12.88	-12.97	-12.71	-12.80	-12.91
1→4	DZ	-15.05	-11.28	-14.02	-13.65	-13.51	*-13.25*	*-13.34*	-13.45
1→6	DZ	-14.66	-10.94	-13.83	-13.20	-13.11	*-12.85*	*-12.94*	*-13.05*
1→2	TZ TZ	-17.62	-13.15	-16.93	-16.03	-16.05	*-15.69*[b]	*-15.82*[b]	*-15.97*[b]
1→4	TZ	-18.44	-13.64	-17.64	-16.65	-16.66	*-16.30*[b]	*-16.43*[b]	*-16.58*[b]
1→6	TZ	*-17.91*	*-13.18*	-16.92	-16.13	*-16.13*	*-15.77*[b]	*-15.90*[b]	*-16.05*[b]
1→2	{T,Q}Z	-19.17	-14.36	-17.78	-17.53	-17.72	*-17.36*[b]	*-17.48*[b]	*-17.63*[b]
1→4	{T,Q}Z	*-20.00*	*-14.85*	-18.52	-18.15	*-18.33*	*-17.97*[b]	*-18.09*[b]	*-18.25*[b]
1→6	{T,Q}Z	*-19.97*	*-15.42*	-18.14	-18.21	*-17.80*	*-17.44*[b]	*-17.57*[b]	*-17.72*[b]
1→2	{Q,5}Z	-19.09	*-14.29*	-17.72	-17.53	*-17.65*	*-17.28*[b]	*-17.41*[b]	*-17.56*[b]
1→4	{Q,5}Z	*-19.92*	*-14.78*	-18.14	-18.09	*-18.26*	*-17.89*[b]	*-18.02*[b]	*-18.18*[b]
1→6	{Q,5}Z	*-18.90*	*-13.64*	-17.53	-17.90	*-17.66*	*-17.29*[b]	*-17.42*[b]	*-17.58*[b]

[a] Numbers in italics are estimates. [b] The post-CCSD(T) slopes have been scaled by a factor of 1.4 to account for the small cc-pVDZ basis set size — see discussion in[S1] and in its Supporting Information.

Table S197: Best available Correlation Energy Slopes (kJ/mol per Ring), including post-CCSD(T) Values, of the BNx_NBx Dimers[a]

		Method							
				CCSD(T)			post-CCSD(T)		
Slopes	Basis Set	MP2	CCSD	`Tight`	`VeryTight`	canonical	CCSDT-2	CCSDT-3	$CCSD(T)_\lambda$
1→2	DZ(d,s)	-19.81	-16.10			-19.07	-18.78	-18.88	-19.00
1→2	DZ	-20.17	-16.17	-19.57	-18.95	-19.19	-18.88	-18.98	-19.10
1→4	DZ	-21.19	-16.88	-20.09	-19.94	-20.06	*-19.75*	*-19.85*	-19.97
1→6	DZ	-20.63	-16.42	-19.63	-19.41	-19.50	*-19.19*	*-19.29*	*-19.41*
1→2	TZ	-20.53	-15.55	-19.00	-18.77	-18.85	*-18.41*[b]	*-18.55*[b]	*-18.72*[b]
1→4	TZ	-21.44	-16.14	-19.86	-19.44	-19.57	*-19.13*[b]	*-19.27*[b]	*-19.44*[b]
1→6	TZ	*-20.79*	*-15.61*	-19.22	-18.83	*-18.96*	*-18.52*[b]	*-18.66*[b]	*-18.83*[b]
1→2	{T,Q}Z	-19.44	-14.29	-17.73	-17.50	-17.64	*-17.21*[b]	*-17.34*[b]	*-17.52*[b]
1→4	{T,Q}Z	*-20.35*	*-14.88*	-18.48	-18.17	*-18.36*	*-17.93*[b]	*-18.06*[b]	*-18.24*[b]
1→6	{T,Q}Z	*-19.66*	*-14.20*	-17.94	-17.71	*-17.75*	*-17.31*[b]	*-17.45*[b]	*-17.62*[b]
1→2	{Q,5}Z	-19.07	*-14.20*	-17.81	-17.49	*-17.61*	*-17.17*[b]	*-17.31*[b]	*-17.48*[b]
1→4	{Q,5}Z	*-19.97*	*-14.80*	-18.38	-18.02	*-18.33*	*-17.89*[b]	*-18.03*[b]	*-18.20*[b]
1→6	{Q,5}Z	*-19.35*	*-14.42*	-17.81	-17.49	*-17.79*	*-17.35*[b]	*-17.48*[b]	*-17.66*[b]

[a] Numbers in italics are estimates. [b] The post-CCSD(T) slopes have been scaled by a factor of 1.4 to account for the small cc-pVDZ basis set size — see discussion in[S1] and in its Supporting Information.

Equations for estimation slopes for BNx_NBx series are as follows:

$$E^{\mathrm{canonical}}_{\mathrm{cc-pVTZ}}(1 \rightarrow 6) \approx E^{\mathrm{canonical}}_{\mathrm{cc-pVTZ}}(1 \rightarrow 4) + E^{\mathrm{LNO,vTight}}_{\mathrm{cc-pVTZ}}(1 \rightarrow 6) - E^{\mathrm{LNO,vTight}}_{\mathrm{cc-pVTZ}}(1 \rightarrow 4) \tag{8}$$

$$E^{\mathrm{canonical}}_{\mathrm{cc-pVQZ}}(1 \rightarrow 4) \approx E^{\mathrm{canonical}}_{\mathrm{cc-pVQZ}}(1 \rightarrow 2) + E^{\mathrm{canonical}}_{\mathrm{cc-pVTZ}}(1 \rightarrow 4) - E^{\mathrm{canonical}}_{\mathrm{cc-pVTZ}}(1 \rightarrow 2) \tag{9}$$

$$E^{\mathrm{canonical}}_{\mathrm{cc-pVQZ}}(1 \rightarrow 6) \approx E^{\mathrm{canonical}}_{\mathrm{cc-pVQZ}}(1 \rightarrow 4) + E^{\mathrm{canonical}}_{\mathrm{cc-pVTZ}}(1 \rightarrow 6) - E^{\mathrm{canonical}}_{\mathrm{cc-pVTZ}}(1 \rightarrow 4) \tag{10}$$

$$E^{\mathrm{canonical}}_{\mathrm{aug-cc-pVQZ}}(1 \rightarrow 2) \approx E^{\mathrm{canonical}}_{\mathrm{cc-pVQZ}}(1 \rightarrow 2) + E^{\mathrm{LNO,vTight}}_{\mathrm{aug-cc-pVQZ}}(1 \rightarrow 2) - E^{\mathrm{LNO,vTight}}_{\mathrm{cc-pVQZ}}(1 \rightarrow 2) \tag{11}$$

$$E^{\mathrm{canonical}}_{\mathrm{cc-pV5Z}}(1 \rightarrow 2) \approx E^{\mathrm{canonical}}_{\mathrm{cc-pVQZ}}(1 \rightarrow 2) + E^{\mathrm{LNO,vTight}}_{\mathrm{cc-pV5Z}}(1 \rightarrow 2) - E^{\mathrm{LNO,vTight}}_{\mathrm{cc-pVQZ}}(1 \rightarrow 2) \tag{12}$$

$$E^{\text{canonical}}_{\text{cc-pV5Z}}(1 \to 4) \approx E^{\text{canonical}}_{\text{cc-pV5Z}}(1 \to 2) + E^{\text{canonical}}_{\text{cc-pVTZ}}(1 \to 4) - E^{\text{canonical}}_{\text{cc-pVTZ}}(1 \to 2) \tag{13}$$

$$E^{\text{LNO,vTight}}_{\text{cc-pV5Z}}(1 \to 6) \approx E^{\text{LNO,vTight}}_{\text{cc-pV5Z}}(1 \to 4) + E^{\text{LNO,vTight}}_{\text{cc-pVQZ}}(1 \to 6) - E^{\text{LNO,vTight}}_{\text{cc-pVQZ}}(1 \to 4) \tag{14}$$

$$E^{\text{canonical}}_{\text{cc-pV5Z}}(1 \to 6) \approx E^{\text{canonical}}_{\text{cc-pV5Z}}(1 \to 4) + E^{\text{LNO,Tight}}_{\text{cc-pV5Z}}(1 \to 6) - E^{\text{LNO,Tight}}_{\text{cc-pV5Z}}(1 \to 4) \tag{15}$$

Table S198: Best available CP-Corrected Correlation Energy Slopes (kJ/mol per Ring), including post-CCSD(T) Values, of the BNx_BNx Dimers[a]

		Method							
				CCSD(T)			post-CCSD(T)		
Slopes	Basis Set	MP2	CCSD	`Tight`	`VeryTight`	canonical	CCSDT-2	CCSDT-3	CCSD(T)$_\lambda$
1→2	DZ(d,s)	-20.98	-15.55			-18.79	-18.35	-18.50	-18.68
1→2	DZ	-21.93	-16.36	-19.42	-19.46	-19.69	-19.15	-19.30	-19.48
1→4	DZ	-22.91	-16.99	-21.02	-20.29	-20.49	*-19.95*	*-20.10*	-20.32
1→6	DZ	-23.20	-17.18	-21.55	-20.58	-20.72	*-20.18*	*-20.33*	*-20.55*
1→2	TZ	-27.48	-20.36	-24.24	-24.59	-24.80	*-24.05*[b]	*-24.26*[b]	*-24.51*[b]
1→4	TZ	-28.32	-20.83	-25.57	-25.34	-25.43	*-24.68*[b]	*-24.89*[b]	*-25.20*[b]
1→6	TZ	*-28.61*	*-21.02*	-25.86	-25.53	*-25.66*	*-24.91*[b]	*-25.12*[b]	*-25.43*[b]
1→2	{T,Q}Z	-30.12	-22.48	-27.23	-27.30	-27.62	*-26.87*[b]	*-27.07*[b]	*-27.33*[b]
1→4	{T,Q}Z	*-30.95*	*-22.96*	-27.81	-27.78	*-28.25*	*-27.50*[b]	*-27.70*[b]	*-28.01*[b]
1→6	{T,Q}Z	*-31.01*	*-22.95*	-28.13	-28.15	*-28.53*	*-27.78*[b]	*-27.98*[b]	*-28.30*[b]
1→2	{Q,5}Z	-30.09	*-22.37*	-28.23	-27.25	*-27.52*	*-26.77*[b]	*-26.98*[b]	*-27.23*[b]
1→4	{Q,5}Z	*-30.55*	*-22.66*	-28.25	-27.68	*-27.92*	*-27.17*[b]	*-27.38*[b]	*-27.69*[b]
1→6	{Q,5}Z	*-30.83*	*-22.86*	-28.20	-27.98	*-28.26*	*-27.51*[b]	*-27.71*[b]	*-28.02*[b]

[a] Numbers in italics are estimates. [b] The post-CCSD(T) slopes have been scaled by a factor of 1.4 to account for the small cc-pVDZ basis set size — see discussion in[S1] and in its Supporting Information.

Table S199: Best available Correlation Energy Slopes (kJ/mol per Ring), including post-CCSD(T) Values, of the BNx_BNx Dimers[a]

		Method							
				CCSD(T)			post-CCSD(T)		
Slopes	Basis Set	MP2	CCSD	`Tight`	`VeryTight`	canonical	CCSDT-2	CCSDT-3	CCSD(T)$_\lambda$
1→2	DZ(d,s)	-32.19	-25.65			-30.57	-30.03	-30.18	-30.42
1→2	DZ	-32.79	-25.91	-30.72	-30.42	-30.91	-30.26	-30.40	-30.65
1→4	DZ	-33.92	-26.74	-31.65	-31.57	-31.95	*-31.31*	*-31.45*	-31.73
1→6	DZ	-34.22	-26.95	-31.97	-31.90	-32.22	*-31.58*	*-31.72*	*-32.00*
1→2	TZ	-32.42	-24.36	-29.49	-29.26	-29.52	*-28.61*[b]	*-28.81*[b]	*-29.15*[b]
1→4	TZ	-33.31	-24.94	-30.19	-29.97	-30.25	*-29.35*[b]	*-29.55*[b]	*-29.94*[b]
1→6	TZ	*-33.61*	*-25.15*	-30.31	-30.14	*-30.52*	*-29.62*[b]	*-29.81*[b]	*-30.21*[b]
1→2	{T,Q}Z	-30.39	-22.28	-27.41	-27.07	-27.38	*-26.48*[b]	*-26.68*[b]	*-27.02*[b]
1→4	{T,Q}Z	*-31.28*	*-22.85*	-27.79	-27.64	*-28.12*	*-27.22*[b]	*-27.41*[b]	*-27.81*[b]
1→6	{T,Q}Z	*-31.23*	*-22.69*	-27.92	-27.70	*-28.20*	*-27.29*[b]	*-27.49*[b]	*-27.89*[b]
1→2	{Q,5}Z	-29.90	*-22.17*	-27.25	-27.01	*-27.30*	*-26.39*[b]	*-26.59*[b]	*-26.93*[b]
1→4	{Q,5}Z	*-30.38*	*-22.39*	-27.82	-27.40	*-27.58*	*-26.68*[b]	*-26.88*[b]	*-27.27*[b]
1→6	{Q,5}Z	*-30.64*	*-22.63*	-27.98	-27.67	*-27.95*	*-27.05*[b]	*-27.25*[b]	*-27.64*[b]

[a] Numbers in italics are estimates. [b] The post-CCSD(T) slopes have been scaled by a factor of 1.4 to account for the small cc-pVDZ basis set size — see discussion in[S1] and in its Supporting Information.

Equations for estimation slopes for BNx_BNx series are as follows:

$$E^{\text{canonical}}_{\text{cc-pVTZ}}(1 \to 6) \approx E^{\text{canonical}}_{\text{cc-pVTZ}}(1 \to 4) + E^{\text{canonical}}_{\text{cc-pVDZ}}(1 \to 6) - E^{\text{canonical}}_{\text{cc-pVDZ}}(1 \to 4) \quad (16)$$

$$E^{\text{canonical}}_{\text{cc-pVQZ}}(1 \to 4) \approx E^{\text{canonical}}_{\text{cc-pVQZ}}(1 \to 2) + E^{\text{canonical}}_{\text{cc-pVTZ}}(1 \to 4) - E^{\text{canonical}}_{\text{cc-pVTZ}}(1 \to 2) \quad (17)$$

$$E^{\text{canonical}}_{\text{cc-pVQZ}}(1 \to 6) \approx E^{\text{canonical}}_{\text{cc-pVQZ}}(1 \to 2) + E^{\text{LNO,vTight}}_{\text{cc-pVQZ}}(1 \to 6) - E^{\text{LNO,vTight}}_{\text{cc-pVQZ}}(1 \to 2) \quad (18)$$

$$E^{\text{canonical}}_{\text{cc-pV5Z}}(1 \to 2) \approx E^{\text{canonical}}_{\text{cc-pVQZ}}(1 \to 2) + E^{\text{LNO,vTight}}_{\text{cc-pV5Z}}(1 \to 2) - E^{\text{LNO,vTight}}_{\text{cc-pVQZ}}(1 \to 2) \quad (19)$$

$$E^{\text{canonical}}_{\text{cc-pV5Z}}(1 \to 4) \approx E^{\text{canonical}}_{\text{cc-pV5Z}}(1 \to 2) + E^{\text{LNO,vTight}}_{\text{cc-pV5Z}}(1 \to 4) - E^{\text{LNO,vTight}}_{\text{cc-pV5Z}}(1 \to 2) \quad (20)$$

$$E^{\mathrm{LNO,vTight}}_{\mathrm{cc-pV5Z}}(1 \rightarrow 6) \approx E^{\mathrm{LNO,vTight}}_{\mathrm{cc-pV5Z}}(1 \rightarrow 4) + E^{\mathrm{LNO,vTight}}_{\mathrm{cc-pVQZ}}(1 \rightarrow 6) - E^{\mathrm{LNO,vTight}}_{\mathrm{cc-pVQZ}}(1 \rightarrow 4) \quad (21)$$

$$E^{\mathrm{canonical}}_{\mathrm{cc-pV5Z}}(1 \rightarrow 6) \approx E^{\mathrm{canonical}}_{\mathrm{cc-pV5Z}}(1 \rightarrow 4) + E^{\mathrm{LNO,vTight}}_{\mathrm{cc-pV5Z}}(1 \rightarrow 6) - E^{\mathrm{LNO,vTight}}_{\mathrm{cc-pV5Z}}(1 \rightarrow 4) \quad (22)$$

Table S200: Counter-poise corrected CCSD(T) and LNO-CCSD(T) correlation contributions to interaction energies of Benzene and Naphthalene PD dimers at various basis sets.

	CCSD(T)		LNO-CCSD(T)			
	canonical		vvTight		vTight	
	Benzene	Naphtalene	Benzene	Naphtalene	Benzene	Naphtalene
DZ	-18.28	-39.03	-18.36	-39.35	-18.42	-39.54
TZ	-23.87	-50.53	-24.17	-51.14	-24.22	-51.30
QZ	-26.03	-54.57	-26.20	-54.92	-26.28	-55.15
{T,Q}Z	-27.61	-57.51	-27.68	-57.68	-27.79	-57.97
5Z	-26.97		-27.11	-56.54	-27.15	-56.77
{Q,5}Z	-27.95		-28.07	-58.23	-28.07	-58.48

Table S201: Counter-poise uncorrected CCSD(T) and LNO-CCSD(T) correlation contributions to interaction energies of Benzene and Naphthalene PD dimers at various basis sets.

	CCSD(T)		LNO-CCSD(T)			
	canonical		vvTight		vTight	
	Benzene	Naphtalene	Benzene	Naphtalene	Benzene	Naphtalene
DZ	-21.65	-47.30	-21.85	-47.86	-21.90	-48.07
TZ	-26.78	-57.31	-26.92	-57.68	-26.96	-57.86
QZ	-27.28	-57.34	-27.42	-57.71	-27.50	-57.99
{T,Q}Z	-27.64	-57.36	-27.79	-57.74	-27.90	-58.09
5Z	-27.56		-27.69	-57.81	-27.76	-58.13
{Q,5}Z	-27.86		-27.97	-57.90	-28.03	-58.27

Table S202: Full CCSD(T) interaction energies of Benzene and Naphthalene PD dimers at various basis set extrapolations. Estimates from LNO-CCSD(T) are in italic.

cp-corrected	Benzene	Naphtalene
{T,Q}Z cor + {Q,5}Z HF	-11.62	-26.98
{Q,5}Z cor + {Q,5}Z HF	-11.96	*-27.54*
cp-uncorrected		
{T,Q}Z cor + {Q,5}Z HF	-11.10	-25.99
{Q,5}Z cor + {Q,5}Z HF	-11.32	*-26.15*

Table S203: Best calculated estimates for the CCSD(T)$_\lambda$ interaction energies of Benzene and Naphthalene dimers.

cp-corrected	Benzene	Naphtalene
CCSD(T)	-11.96	-27.54
CCSD(T)$_\lambda$ difference	0.21	0.33
CCSD(T)+ scaled CCSD(T)$_\lambda$ difference	-11.67	-27.08
cp-uncorrected	Benzene	Naphtalene
CCSD(T)	-11.32	-26.15
CCSD(T)$_\lambda$ difference	0.12	0.43
CCSD(T)+ scaled CCSD(T)$_\lambda$ difference	-11.15	-25.55

Table S204: Counter-poise corrected CCSD(T) and LNO-CCSD(T) correlation contributions to interaction energies of BNx_NBx-Borazine and BNx_NBx-Naphthalene dimers at various basis sets.

	CCSD(T)		LNO-CCSD(T)			
	canonical		vvTight		vTight	
	BN1_NB1	BN2_NB2	BN1_NB1	BN2_NB2	BN1_NB1	BN2_NB2
DZ	-13.04	-26.01	-13.12	-26.01	-13.07	-25.95
TZ	-17.09	-33.14	-16.91	-32.95	-16.78	-32.82
QZ	-18.57	-35.59	-18.43	-35.28	-18.27	-35.16
{T,Q}Z	-19.65	-37.37	-19.54	-36.98	-19.35	-36.87
5Z	-19.11		-18.95	-36.40	-18.84	-36.04
{Q,5}Z	-19.68		-19.48	-37.58	-19.43	-36.96

Table S205: Counter-poise uncorrected CCSD(T) and LNO-CCSD(T) correlation contributions to interaction energies of BNx_NBx-Borazine and BNx_NBx-Naphthalene dimers at various basis sets.

	CCSD(T)		LNO-CCSD(T)			
	canonical		vvTight		vTight	
	BN1_NB1	BN2_NB2	BN1_NB1	BN2_NB2	BN1_NB1	BN2_NB2
DZ	-18.34	-37.54	-18.14	-37.10	-18.06	-37.01
TZ	-19.31	-38.16	-19.21	-37.95	-19.03	-37.80
QZ	-19.60	-37.75	-19.49	-37.47	-19.34	-37.37
{T,Q}Z	-19.81	-37.45	-19.69	-37.12	-19.56	-37.06
5Z	-19.60		-19.47	-37.42	-19.35	-37.12
{Q,5}Z	-19.61		-19.45	-37.36	-19.36	-36.85

Table S206: Full CCSD(T) interaction energies of BNx_NBx-Borazine and BNx_NBx-Naphthalene dimers at various basis set extrapolations. Estimates from LNO-CCSD(T) are in italic.

cp-corrected	BN1_NB1	BN2_NB2
{T,Q}Z cor + {Q,5}Z HF	-7.81	-15.21
{Q,5}Z cor + {Q,5}Z HF	-7.84	*-15.30*
cp-uncorrected		
{T,Q}Z cor + {Q,5}Z HF	-7.28	-14.22
a{Q,5}Z cor + {Q,5}Z HF	-7.09	*-14.01*

Table S207: Best calculated estimates for the CCSD(T)$_\lambda$ interaction energies of BNx_NBx-Borazine and BNx_NBx-Naphthalene dimers.

cp-corrected	BN1_NB1	BN2_NB2
CCSD(T)	-7.84	-15.30
CCSD(T)$_\lambda$ difference	0.08	0.14
CCSD(T)+ scaled CCSD(T)$_\lambda$ difference	-7.72	-15.10
cp-uncorrected	Benzene	Naphtalene
CCSD(T)	-7.09	-14.01
CCSD(T)$_\lambda$ difference	0.10	0.20
CCSD(T)+ scaled CCSD(T)$_\lambda$ difference	-6.94	-13.73

Table S208: Counter-poise corrected CCSD(T) and LNO-CCSD(T) correlation contributions to interaction energies of BNx_BNx-Borazine and BNx_BNx-Naphthalene dimers at various basis sets.

	CCSD(T)		LNO-CCSD(T)			
	canonical		vvTight		vTight	
	BN1_BN1	BN2_BN2	BN1_BN1	BN2_BN2	BN1_BN1	BN2_BN2
DZ	-16.71	-36.40	-16.58	-36.11	-16.40	-35.86
TZ	-23.32	-48.12	-22.99	-47.63	-22.76	-47.36
QZ	-25.55	-51.98	-24.50	-51.47	-25.05	-51.20
{T,Q}Z	-27.18	-54.80	-25.60	-54.27	-26.71	-54.01
5Z	-26.42		-26.25	-53.24	-26.04	-52.73
{Q,5}Z	-27.32		-28.09	-55.10	-27.08	-54.32

Table S209: Counter-poise uncorrected CCSD(T) and LNO-CCSD(T) correlation contributions to interaction energies of BNx_BNx-Borazine and BNx_BNx-Naphthalene dimers at various basis sets.

	CCSD(T)		LNO-CCSD(T)			
	canonical		vvTight		vTight	
	BN1_BN1	BN2_BN2	BN1_BN1	BN2_BN2	BN1_BN1	BN2_BN2
DZ	-25.82	-56.72	-25.76	-56.29	-25.55	-55.98
TZ	-27.72	-57.24	-27.47	-56.75	-27.21	-56.47
QZ	-27.28	-55.56	-27.05	-55.06	-26.83	-54.82
{T,Q}Z	-26.96	-54.34	-26.75	-53.83	-26.55	-53.62
5Z	-27.15		-26.94	-54.77	-26.73	-54.24
{Q,5}Z	-27.01		-26.82	-54.46	-26.62	-53.63

Table S210: Full CCSD(T) interaction energies of BNx_BNx-Borazine and BNx_BNx-Naphthalene dimers at various basis set extrapolations. Estimates from LNO-CCSD(T) are in italic.

cp-corrected	BN1_BN1	BN2_BN2
{T,Q}Z cor + {Q,5}Z HF	-14.90	-30.77
{Q,5}Z cor + {Q,5}Z HF	-15.04	*-31.60*
cp-uncorrected		
{T,Q}Z cor + {Q,5}Z HF	-13.52	-28.48
{Q,5}Z cor + {Q,5}Z HF	-13.57	*-29.11*

Table S211: Best calculated estimates for the CCSD(T)$_\lambda$ interaction energies of BNx_BNx-Borazine and BNx_BNx-Naphthalene dimers.

cp-corrected	BN1_BN1	BN2_BN2
CCSD(T)	-15.04	-31.60
CCSD(T)$_\lambda$ difference	0.11	0.32
CCSD(T)+ scaled CCSD(T)$_\lambda$ difference	-14.88	-31.15
cp-uncorrected	BN1_BN1	BN2_BN2
CCSD(T)	-13.57	-29.11
CCSD(T)$_\lambda$ difference	0.14	0.40
CCSD(T)+ scaled CCSD(T)$_\lambda$ difference	-13.38	-28.55

Tables S2 and S2 shows how noncovalent interaction energy of parallel displaced benzene dimer converges toward to the canonical-CCSD(T)/CBS along LNO-CCSD(T) thresholds and hierarchical number of cc-pVnZ.

Results for Coronene Systems

In this section we show all NCI for coronene systems, such as coronene parallel displaced dimer (C2C2_PD), coronene sandwich-structured dimer (C2C2) and circumcoronene sandwich-structured dimer (C3C3).

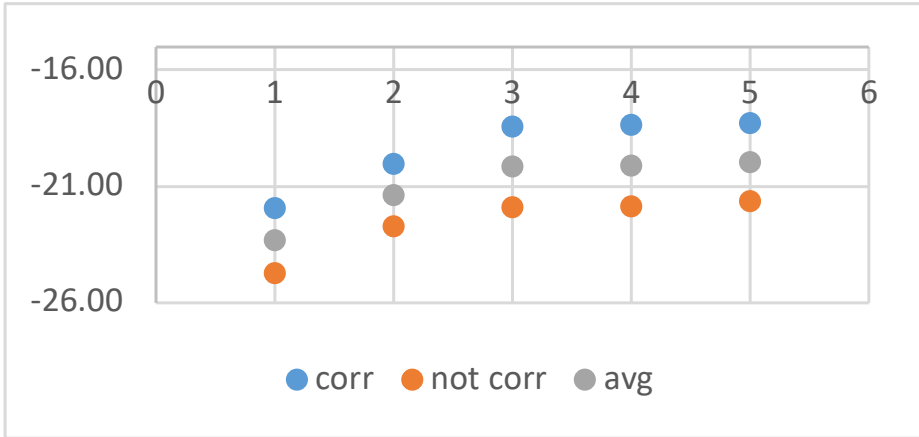

(a) DZ energies

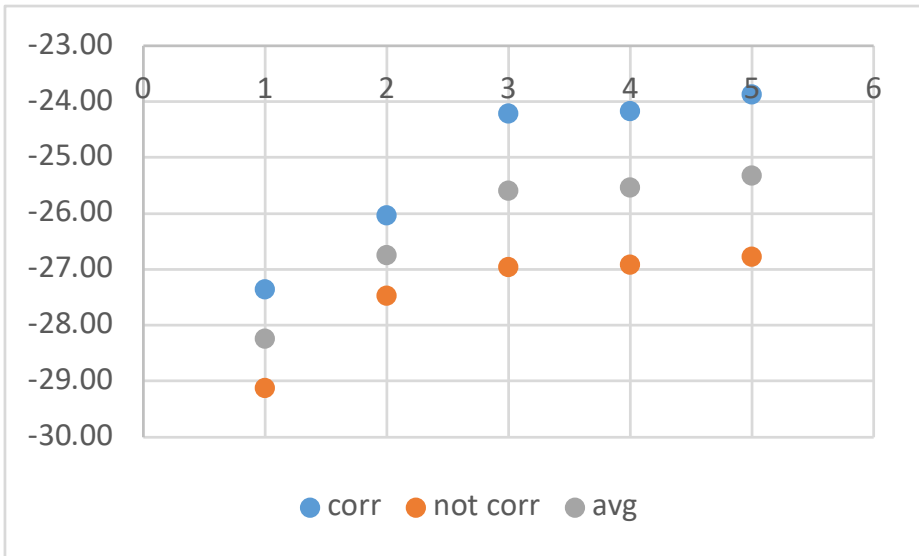

(b) TZ energies

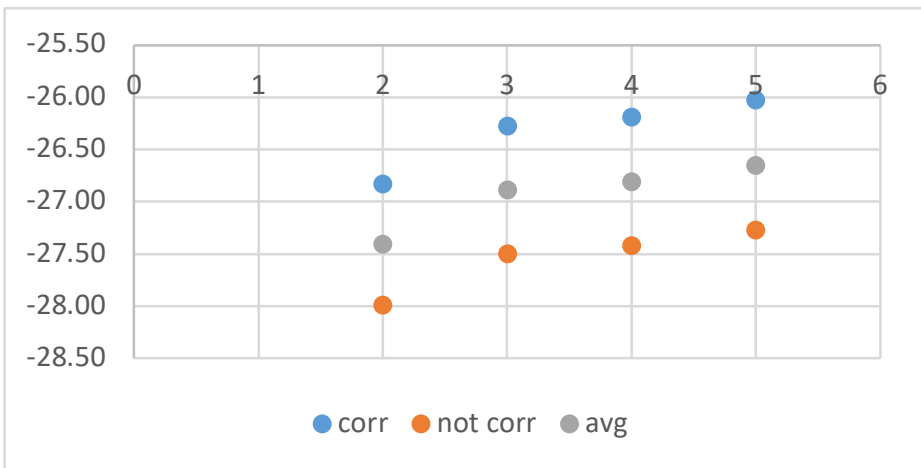

(c) QZ

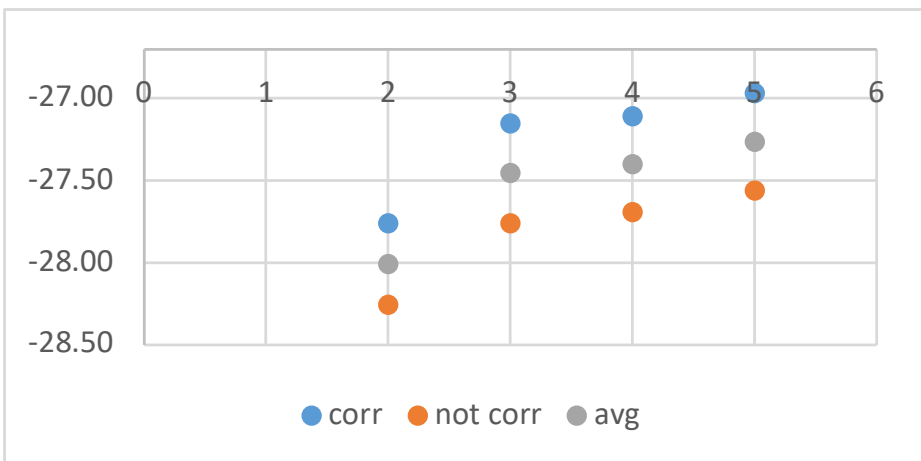

(d) 5Z energies

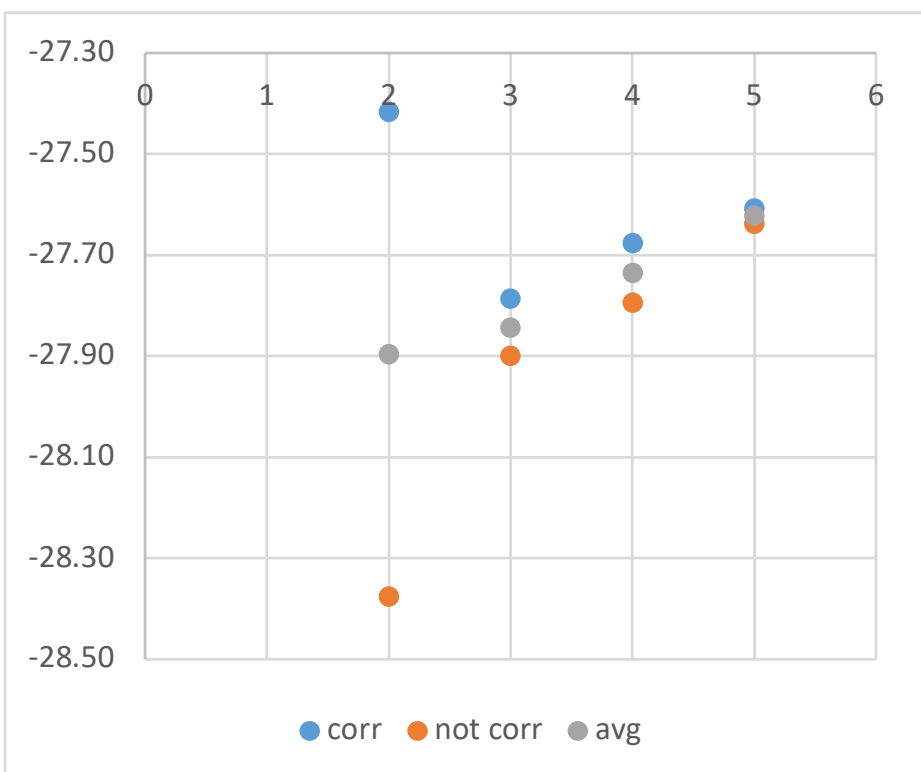

(e) {T,Q}Z energies

Figure S2: cc-pVnZ Interaction energies (kJ/mol) for Benzene PD derived from various LNO-CCSD(T) thresholds in as a function (Normal→Tight→vTight→vvTight→canonical).

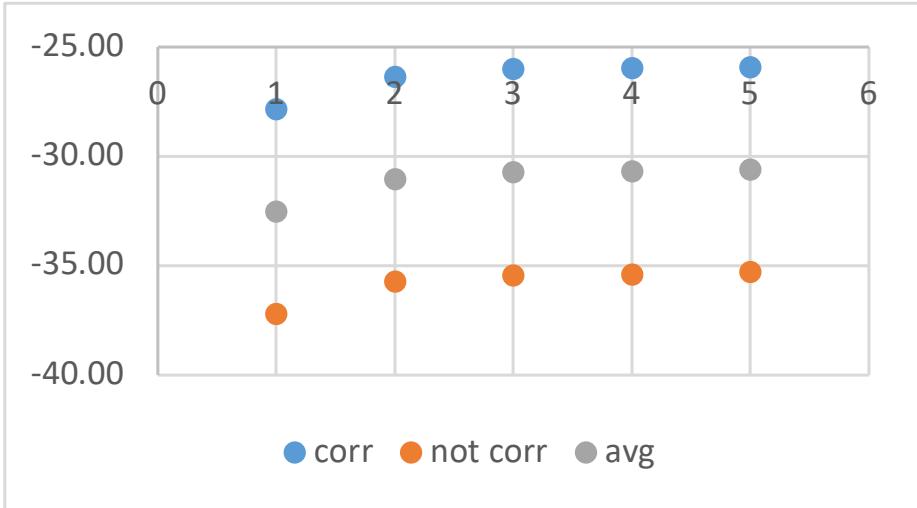

(a) DZ energies

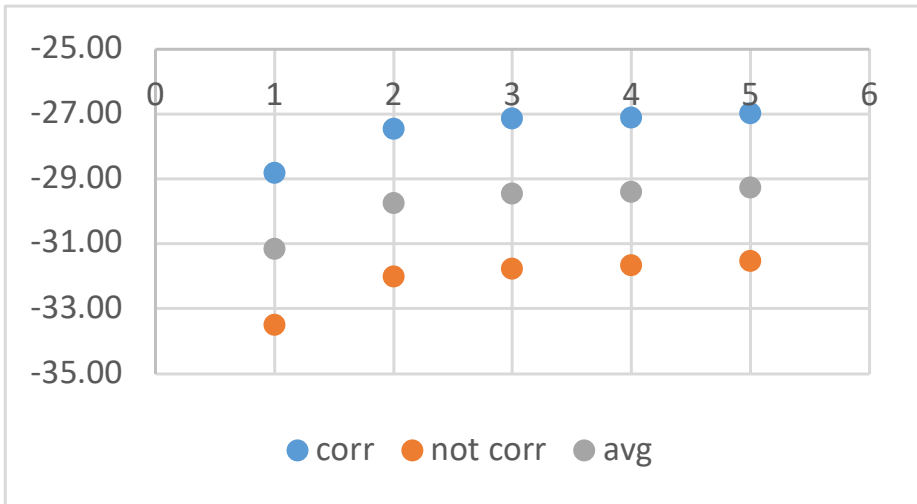

(b) TZ energies

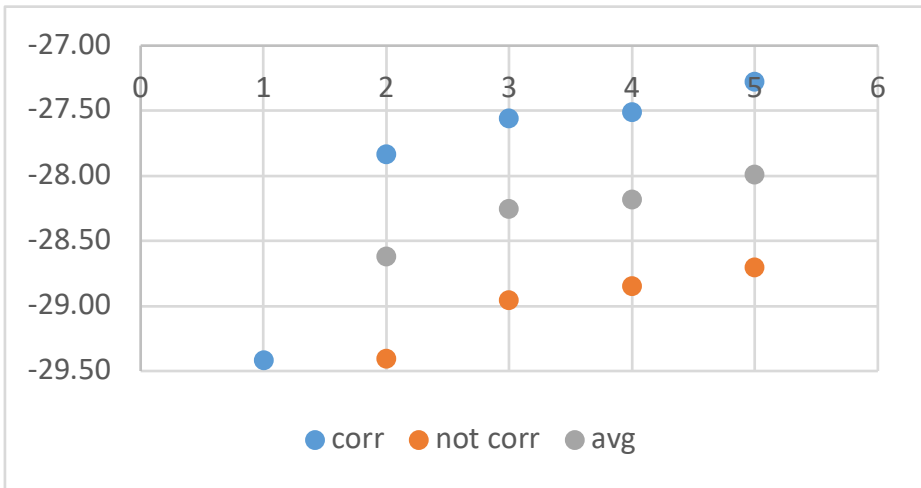

(c) QZ

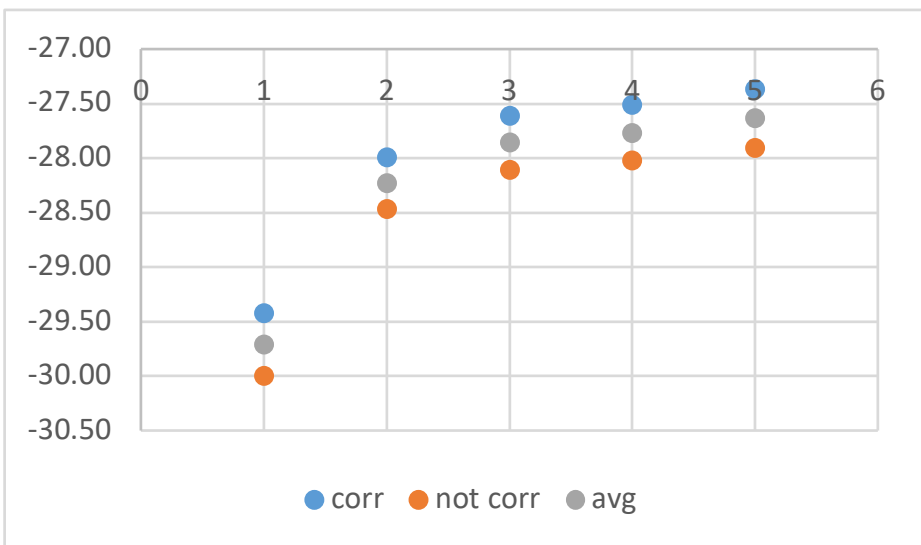

(d) 5Z energies

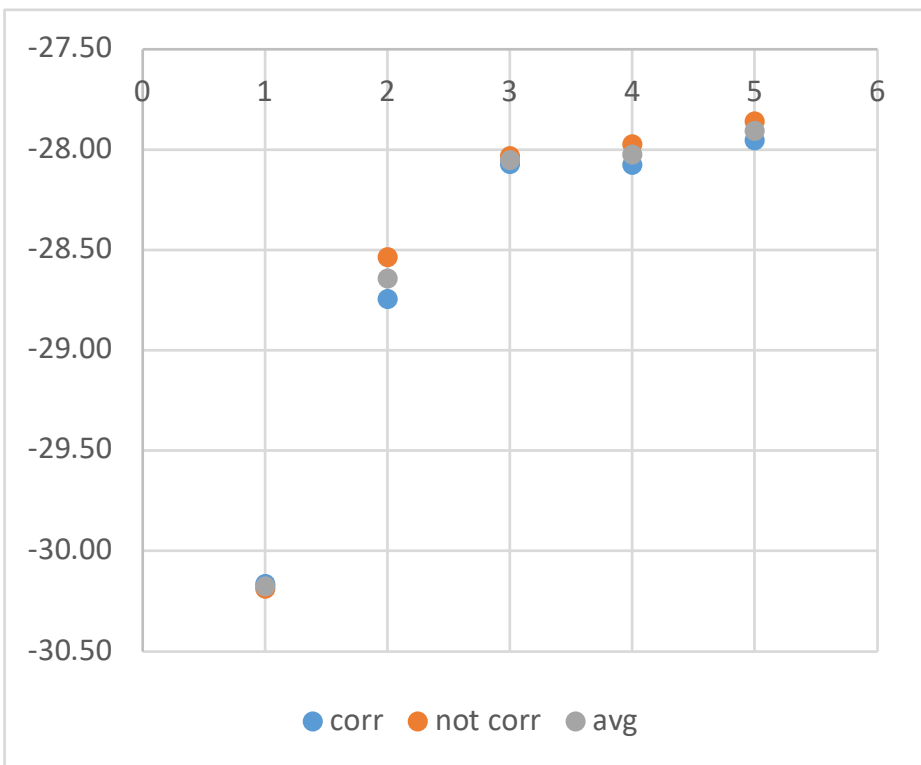

(e) {T,Q}Z energies

Figure S3: aug-cc-pVnZ Interaction energies (kJ/mol) for Benzene PD derived from various LNO-CCSD(T) thresholds in as a function (Normal→Tight→vTight→vvTight→canonical).

Table S212: Hartree-Fock part of the interaction energies in kJ/mol, cp-corrected basis sets for the coronene systems.

Basis	System		
	C2C2_PD	C2C2	C3C3
DZ	65.56	72.39	160.24
TZ	67.27	72.88	164.93
aTZ	66.84		
QZ	67.11	72.82	164.88
aug-QZ	66.81	72.64	
5Z	66.81	72.64	
aug-5Z	66.80	72.63	

Table S213: Hartree-Fock part of the interaction energies in kJ/mol, cp-uncorrected basis sets for the coronene systems.

Basis	System		
	C2C2_PD	C2C2	C3C3
DZ	48.83	59.10	131.36
TZ	61.52	67.85	154.49
aTZ	62.09		
QZ	64.75	70.87	160.94
aug-QZ	65.77	71.72	
5Z	66.40	72.30	
aug-5Z	66.66	72.51	

Table S214: Correlation contributions of post-HF methods to the Interaction energies (in kJ/mol) for Coronenes using cp-corrected cc-pVDZ basis set.

DZ	Method				
System	MP2	CCSD	CCSD(T)	CCSD(cT-fit)	CCSD(T)$_\lambda$
C2C2_PD	-170.50	-90.10	-109.48	-104.98	-105.55
C2C2	-150.91	-81.54	-98.96	-95.03	-98.19

Table S215: Correlation contributions of post-HF methods to the Interaction energies (in kJ/mol) for Coronenes using cp-uncorrected cc-pVDZ basis set.

DZ	Method				
System	MP2	CCSD	CCSD(T)	CCSD(cT-fit)	CCSD(T)$_\lambda$
C2C2_PD	-195.39	-108.03	-131.93	-126.72	-128.55
C2C2	-168.94	-94.47	-115.23	-110.77	-114.27

Table S216: Correlation contributions of LNO-CCSD, including MP2corr, to the Interaction energies (in kJ/mol) for Coronenes using cp-corrected basis sets.

	CORONENE PD				CORONENE SANDWICH			
Basis set	Normal	Tight	vTight	vvTight	Normal	Tight	vTight	vvTight
DZ	-97.33	-94.55	-92.67	-92.29	-129.97	-86.70	-85.01	-84.83
TZ		-119.30	-115.96			-106.45	-103.54	
QZ		-123.79	-122.06			-110.20	-108.91	
aug-QZ		-125.99				-112.46		
aug-5Z		-125.54				-112.35		

Table S217: Correlation contributions of LNO-CCSD, including MP2corr, to the Interaction energies (in kJ/mol) for Coronenes using cp-uncorrected basis sets.

	CORONENE PD				CORONENE SANDWICH			
Basis set	Normal	Tight	vTight	vvTight	Normal	Tight	vTight	vvTight
DZ	-111.41	-112.01	-110.74	-110.13	-99.12	-99.16	-98.35	-97.85
TZ		-132.01	-131.21			-116.08	-115.46	
QZ		-129.40	-128.44			-114.85	-113.83	
aug-QZ		-132.58				-118.96		
aug-5Z		-127.41				-113.81		

Table S218: Correlation contributions of LNO-CCSD(T), including MP2corr, to the Interaction energies (in kJ/mol) for Coronenes using cp-corrected basis sets.

	CORONENE PD				CORONENE SANDWICH			
Basis set	Normal	Tight	vTight	vvTight	Normal	Tight	vTight	vvTight
DZ	-131.44	-118.43	-112.66	-111.45	-119.76	-109.51	-103.57	-102.51
TZ		-149.96	-142.98			-134.58	-128.09	
QZ		-157.08	-151.81			-140.98	-135.66	
aug-QZ		-160.76				-144.20		
aug-5Z		-159.65				-144.19		

Table S219: Correlation contributions of LNO-CCSD(T), including MP2corr, to the Interaction energies (in kJ/mol) for Coronenes using cp-uncorrected basis sets.

	CORONENE PD				CORONENE SANDWICH			
Basis set	Normal	Tight	vTight	vvTight	Normal	Tight	vTight	vvTight
DZ	-152.68	-140.63	-135.64	-133.95	-138.34	-125.73	-120.62	-118.98
TZ		-166.49	-162.00			-147.18	-142.84	
QZ		-164.49	-159.79			-147.18	-141.82	
aug-QZ		-167.92				-151.75		
aug-5Z		-162.25				-146.20		

Table S220: Best available CP-Corrected Correlation Energy Slopes (kJ/mol per Ring) of the C2C2_PD Dimer[a]

		Method					
				CCSD(T)			
Slopes	Basis Set	MP2	CCSD	Tight	VeryTight	canonical	CCSD(T)$_\lambda$
1→7	DZ	-24.35	-12.43	-16.40	-15.71	-15.20	*-15.06*
1→7	TZ	-29.84	*-15.55*	-20.65	-19.79	*-19.29*	*-19.10*[b]
1→7	QZ	-31.16	*-16.30*	-21.71	-20.92	*-20.41*	*-20.23*[b]
1→7	5Z	-31.55	*-16.44*	-21.98	*-21.19*	*-20.69*	*-20.50*[b]
1→7	{T,Q}Z	-32.12	*-16.86*	-22.48	-21.74	*-21.24*	*-21.05*[b]
1→7	{Q,5}Z	-31.96	*-16.59*	-22.27	*-21.48*	*-20.97*	*-20.78*[b]

[a] Numbers in italics are estimates. [b] The post-CCSD(T) slopes have been scaled by a factor of 1.4 to account for the small cc-pVDZ basis set size — see discussion in[S1] and in its Supporting Information.

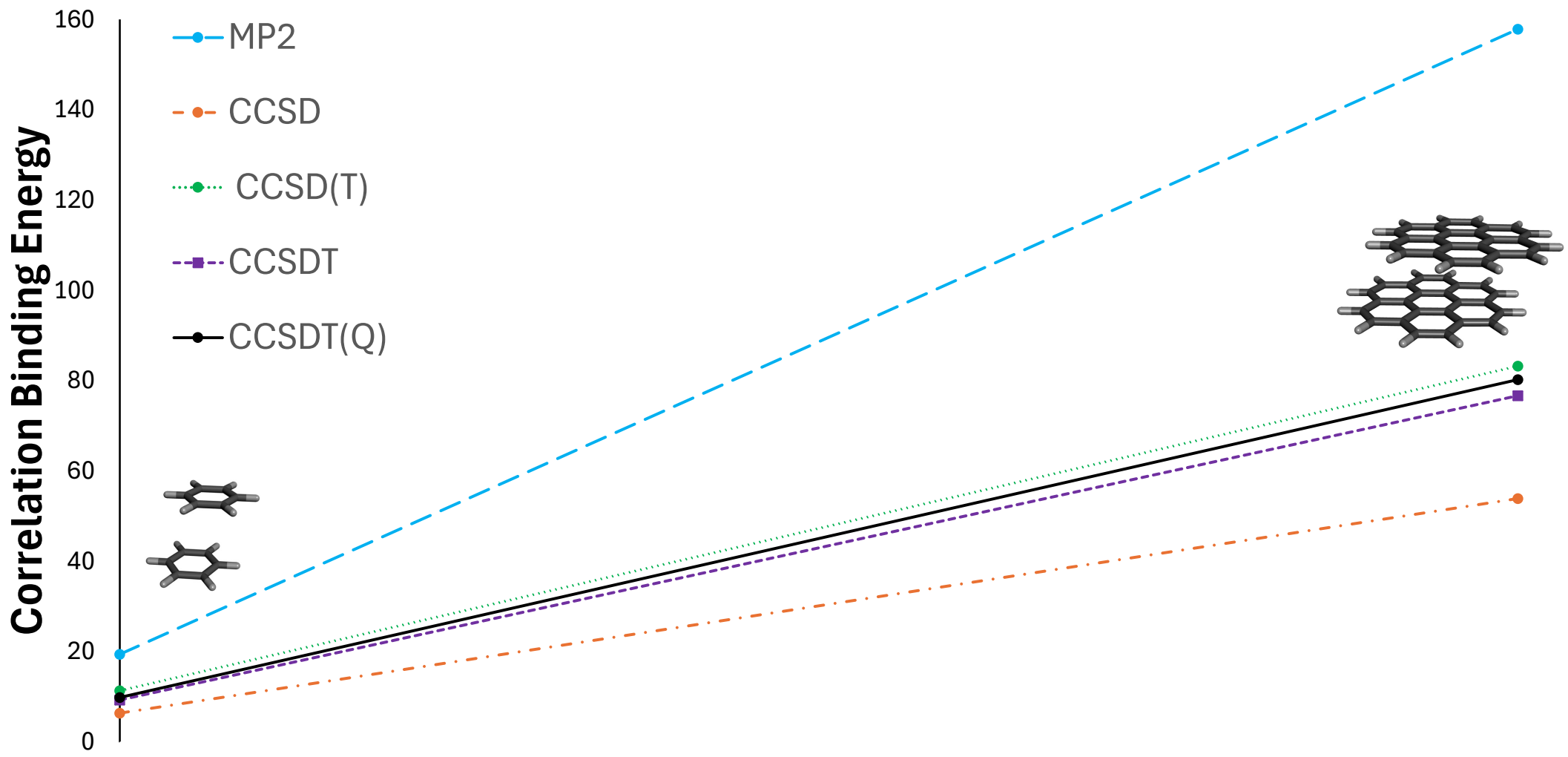

Figure S4: cp-corrected NCI energies in complete basis set (kJ/mol) derived from various methods for the parallel-displaced benzene and coronene dimers.

Table S221: Best available Correlation Energy Slopes (kJ/mol per Ring) of the C2C2_PD Dimer[a]

		Method					
				CCSD(T)			
Slopes	Basis Set	MP2	CCSD	Tight	VeryTight	canonical	CCSD(T)$_\lambda$
1→7	DZ	-27.86	-14.97	-19.65	-18.96	-18.38	*-18.19*
1→7	TZ	-32.69	*-17.72*	-23.17	-22.51	*-21.93*	*-21.66*[b]
1→7	QZ	-32.51	*-17.20*	-22.75	-22.05	*-21.47*	*-21.20*[b]
1→7	5Z	-32.34	*-16.99*	-22.53	*-21.82*	*-21.25*	*-20.98*[b]
1→7	{T,Q}Z	-32.38	*-16.83*	-22.44	-21.71	*-21.14*	*-20.87*[b]
1→7	{Q,5}Z	-32.15	*-16.76*	-22.29	*-21.59*	*-21.01*	*-20.74*[b]

[a] Numbers in italics are estimates. [b] The post-CCSD(T) slopes have been scaled by a factor of 1.4 to account for the small cc-pVDZ basis set size — see discussion in[S1] and in its Supporting Information.

Table S222: Interaction energies for the coronene PD dimer at the different level of theory in complete basis set. All values are reported in kJ/mol.

Level of Theory	Interaction energy
MP2	-161.6 (cp-corr {T,Q}Z)
MP2	-163.5 (cp-uncorr {T,Q}Z)
MP2	-159.3 (cp-corr a{T,Q}Z)
MP2	-152.8 (cp-uncorr a{T,Q}Z)
MP2	-161.4 (cp-corr {Q,5}Z)
MP2	-161.0 (cp-uncorr {Q,5}Z)
MP2	-159.2 (cp-corr a{Q,5}Z)
MP2	-154.3 (cp-uncorr a{Q,5}Z)
CCSD	-57.0 (cp-corr {T,Q}Z)
CCSD	-56.6 (cp-uncorr {T,Q}Z)
CCSD	-55.7 (cp-corr {Q,5}Z)
CCSD	-55.0 (cp-uncorr {Q,5}Z)
LNO-CCSD(T)-Tight	-95.8 (cp-corr {Q,5}Z)
LNO-CCSD(T)-Tight	-94.2 (cp-uncorr {Q,5}Z)
LNO-CCSD(T)-vTight	-90.6 (cp-corr {Q,5}Z)
LNO-CCSD(T)-vTight	-89.4 (cp-uncorr {Q,5}Z)
CCSD(T)	-87.4 (cp-corr {Q,5}Z)
CCSD(T)	-85.7 (cp-uncorr {Q,5}Z)
CCSD(T)$_\lambda$	-86.0 (cp-corr {Q,5}Z)
CCSD(T)$_\lambda$	-83.8 (cp-uncorr {Q,5}Z)
CCSDT	-80.8 (cp-corr {Q,5}Z)
CCSDT	-77.5 (cp-uncorr {Q,5}Z)
CCSDT(Q)	-84.4 (cp-corr {Q,5}Z)
CCSDT(Q)	-81.8 (cp-uncorr {Q,5}Z)

Table S223: Best available CP-Corrected Correlation Energy Slopes (kJ/mol per Ring) of the C2C2 sandwich-structured Dimer[a]

		Method					
				CCSD(T)			
Slopes	Basis Set	MP2	CCSD	`Tight`	`VeryTight`	canonical	CCSD(T)$_\lambda$
1→7	DZ	-21.81	-11.42	-15.50	-14.63	-13.95	-13.84
1→7	TZ	-26.42	*-14.10*	-19.00	-18.10	*-17.42*	*-17.28*[b]
1→7	QZ	-27.67	*-14.73*	-19.85	-19.11	*-18.44*	*-18.29*[b]
1→7	5Z	-28.09	*-14.91*	-20.15	*-19.41*	*-18.73*	*-18.59*[b]
1→7	{T,Q}Z	-28.59	*-15.19*	-20.48	-19.85	*-19.18*	*-19.03*[b]
1→7	{Q,5}Z	-28.52	*-15.10*	-20.46	*-19.72*	*-19.04*	*-18.90*[b]

[a] Numbers in italics are estimates. [b] The post-CCSD(T) slopes have been scaled by a factor of 1.4 to account for the small cc-pVDZ basis set size — see discussion in[S1] and in its Supporting Information.

Table S224: Best available Correlation Energy Slopes (kJ/mol per Ring) of the C2C2 sandwich-structured Dimer[a]

		Method					
				CCSD(T)			
Slopes	Basis Set	MP2	CCSD	`Tight`	`VeryTight`	canonical	CCSD(T)$_\lambda$
1→7	DZ	-24.53	-13.41	-18.02	-17.24	-16.43	-16.30
1→7	TZ	-28.74	*-15.74*	-20.87	-20.27	*-19.47*	*-19.28*[b]
1→7	QZ	-28.79	*-15.41*	-20.76	-20.01	*-19.20*	*-19.02*[b]
1→7	5Z	-28.73	*-15.26*	-20.60	*-19.85*	*-19.04*	*-18.86*[b]
1→7	{T,Q}Z	-28.82	*-15.17*	-20.69	-19.82	*-19.01*	*-18.82*[b]
1→7	{Q,5}Z	-28.66	*-15.10*	-20.43	*-19.68*	*-18.87*	*-18.69*[b]

[a] Numbers in italics are estimates. [b] The post-CCSD(T) slopes have been scaled by a factor of 1.4 to account for the small cc-pVDZ basis set size — see discussion in[S1] and in its Supporting Information.

Equations for estimation 1→7 slopes both parallel displaced and sandwich-structured Coronenes are as follows:

$$E^{\text{canonical}}_{\text{cc-pVTZ}}(1 \to 7) \approx E^{\text{canonical}}_{\text{cc-pVDZ}}(1 \to 7) + E^{\text{LNO,vTight}}_{\text{cc-pVTZ}}(1 \to 7) - E^{\text{LNO,vTight}}_{\text{cc-pVDZ}}(1 \to 7) \tag{23}$$

$$E^{\text{canonical}}_{\text{cc-pVQZ}}(1 \to 7) \approx E^{\text{canonical}}_{\text{cc-pVDZ}}(1 \to 7) + E^{\text{LNO,vTight}}_{\text{cc-pVQZ}}(1 \to 7) - E^{\text{LNO,vTight}}_{\text{cc-pVDZ}}(1 \to 7) \tag{24}$$

$$E^{\text{LNO,vTight}}_{\text{cc-pV5Z}}(1 \to 7) \approx E^{\text{LNO,vTight}}_{\text{cc-pVQZ}}(1 \to 7) + E^{\text{LNO,Tight}}_{\text{cc-pV5Z}}(1 \to 7) - E^{\text{LNO,Tight}}_{\text{cc-pVQZ}}(1 \to 7) \tag{25}$$

$$E^{\text{canonical}}_{\text{cc-pV5Z}}(1 \to 7) \approx E^{\text{canonical}}_{\text{cc-pVDZ}}(1 \to 7) + E^{\text{LNO,vTight}}_{\text{cc-pV5Z}}(1 \to 7) - E^{\text{LNO,vTight}}_{\text{cc-pVDZ}}(1 \to 7) \tag{26}$$

Table S225: Correlation contributions of LNO-CCSD and LNO-CCSD(T) methods to the Interaction energies (in kJ/mol) for Circumcoronene, including MP2 correlation part

Method-Threshold	Basis set			
	DZ cp-corr	TZ cp-corr	DZ cp-uncorr	TZ cp-uncorr
LNO-CCSD-Normal	-237.08	-286.95	-261.95	-302.38
LNO-CCSD(T)-Normal	-335.15	-398.63	-375.17	-427.55
LNO-CCSD-Tight	-224.98	-278.08	-256.62	-297.84
LNO-CCSD(T)-Tight	-286.51	-347.93	-333.79	-385.06

Figure S5 proofs how correlation part of LNO-CCSD(T)-Tight Method aligns well with a linear trend.

Strong Correlation Diagnostic Dimers

We performed a strong correlation diagnostic for the acene PD, BNx_NBx and BNx_BNx series based on total atomization energy (TAE) calculations obtained with the cc-pVTZ basis set, both with the exchange part of TPSS as well as TPSS@HF, as written in[S2]
We used the formulas from the paper:

$$\%TAE_X[\Delta X, TPSS] = 100\% \times \frac{(TAE[X, TPSS@HF] - TAE[X, HF])}{TAE[CCSD(T)]} \tag{27}$$

and

$$\%TAE_X[\Delta X, KS - HF(TPSS)] = 100\% \times \frac{(TAE[X, TPSS@TPSS] - TAE[X, TPSS@HF]}{TAE[CCSD(T)]} \tag{28}$$

In equation 27, the difference in the total atomization energies between the TPSS exchange using a HF exchange wavefunction ($TAE[X, TPSS@HF]$) and the Hartree-Fock exchange part ($TAE[X, HF]$) is considered, divided by the total atomization energy of CCSD(T). In equation 28, the Hartree-Fock exchange part was replaced by the TPSS exchange at the optimized TPSS wavefunction.
The results indicate a minimal static correlation less than 2 % for BNx_NBx and BNx_BNx series (for example, water has a larger strong correlation diagnostic) and around 5 % for parallel displaced

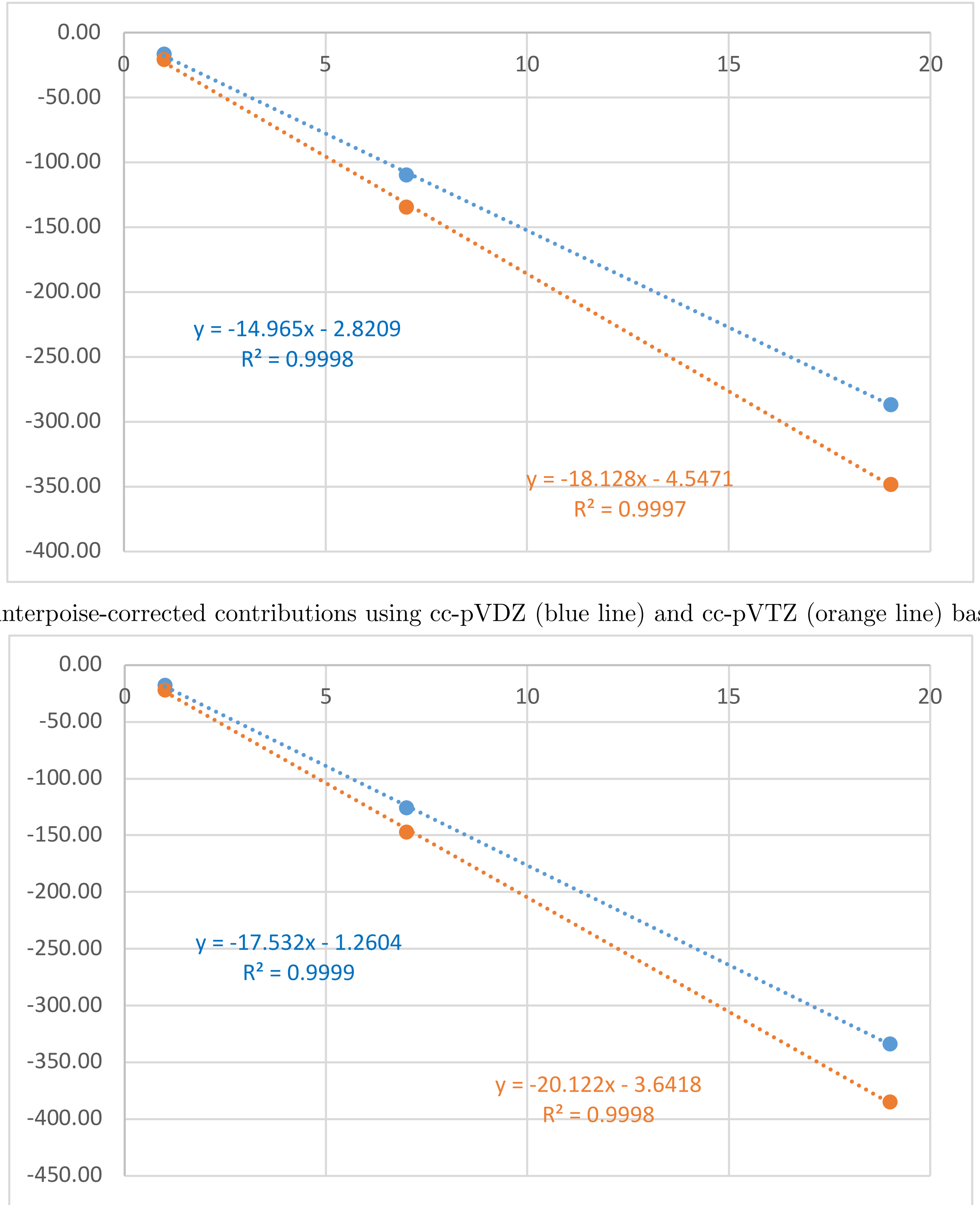

(a) Counterpoise-corrected contributions using cc-pVDZ (blue line) and cc-pVTZ (orange line) basis sets.

(b) Counterpoise-uncorrected contributions using cc-pVDZ (blue line) and cc-pVTZ (orange line) basis sets.

Figure S5: Correlation contributions to interaction energies in kJ/mol vs. number of rings at the LNO-CCSD(T) level with Tight threshold including MP2 corrections.

acene series, not changing when the chain get bigger.

Table S226: Total Atomization energies (in kJ/mol) for the acene PD dimers using a cc-pVTZ basis set.

TZ	Cx_Cx_pd		TAEs				
System	Rings	X_TPSS@TPSS	X_TPSS@HF	HF	HF	CCSD(T)	LNO-CCSD(T)
				ORCA	MRCC		Tight
c1_c1_pd	1	-5849.94	-5282.00	-8554.40	-8553.82	-11157.10	-11159.23
c2_c2_pd	2	-9475.22	-8565.09	-13430.26	-13429.25	-17634.82	-17639.63
c3_c3_pd	3	-13086.05	-11833.90	-18264.00	-18262.56	-24083.56	-24089.97
c4_c4_pd	4	-16688.27	-15093.03	-23076.80	-23074.94	-30519.42	-30527.26
c5_c5_pd	5	-20295.74	-18357.90	-27877.32	-27875.04		-36957.56
c6_c6_pd	6	-23898.00	-21614.22	-32670.75	-32668.05		-43384.80

Table S227: Strong Correlation Diagnostic (in %) derived from TAEs of the acene PD dimers calculated using a cc-pVTZ basis set.

TZ	Cx_Cx_pd				
System	Rings	(TPSS@HF-HF)	(TPSS@HF-HF)	(TPSS@TPSS-TPSS@HF)	(TPSS@TPSS-TPSS@HF)
		CCSD(T)	LNO-CCSD(T) Tight	CCSD(T)	LNO-CCSD(T) Tight
c1_c1_pd	1	-29.33	-29.32	5.09	5.09
c2_c2_pd	2	-27.59	-27.58	5.16	5.16
c3_c3_pd	3	-26.70	-26.69	5.20	5.20
c4_c4_pd	4	-26.16	-26.15	5.23	5.23
c5_c5_pd	5		-25.76		5.24
c6_c6_pd	6		-25.48		5.26

Table S228: Total Atomization energies (in kJ/mol) for the BNx_NBx dimers using a cc-pVTZ basis set.

TZ	BNx_NBx		TAEs				
System	Rings	X_TPSS@TPSS	X_TPSS@HF	HF	HF	CCSD(T)	LNO-CCSD(T)
				ORCA	MRCC		Tight
bn1_nb1	1	-5326.54	-5153.44	-7584.61	-7584.11	-9934.35	-9937.49
bn2_nb2	2	-8705.03	-8447.02	-11998.43	-11997.60	-15742.88	-15746.81
bn3_nb3	3	-12084.08	-11739.81	-16408.67	-16407.51	-21550.69	-21555.34
bn4_nb4	4	-15447.30	-15018.48	-20818.07	-20816.58	-27359.30	-27364.68
bn5_nb5	5	-18838.97	-18323.12	-25232.42	-25230.61		-33173.60
bn6_nb6	6	-22219.13	-21620.35	-29641.05	-29638.91		-38982.06

Table S229: Strong Correlation Diagnostic (in %) derived from TAEs of the BNx_NBx dimers calculated using a cc-pVTZ basis set.

TZ	BNx_NBx				
System	Rings	(TPSS@HF-HF)	(TPSS@HF-HF)	(TPSS@TPSS-TPSS@HF)	(TPSS@TPSS-TPSS@HF)
		CCSD(T)	LNO-CCSD(T) Tight	CCSD(T)	LNO-CCSD(T) Tight
bn1_nb1	1	-24.47	-24.46	1.74	1.74
bn2_nb2	2	-22.56	-22.55	1.64	1.64
bn3_nb3	3	-22.20	-21.66	1.64	1.60
bn4_nb4	4	-21.20	-21.19	1.57	1.57
bn5_nb5	5		-20.83		1.55
bn6_nb6	6		-20.58		1.54

Table S230: Total Atomization energies (in kJ/mol) for the BNx_BNx dimers using a cc-pVTZ basis set.

TZ	BNx_BNx		TAEs				
System	Rings	X_TPSS@TPSS	X_TPSS@HF	HF	HF	CCSD(T)	LNO-CCSD(T)
				ORCA	MRCC		Tight
bn1_bn1	1	-5322.51	-5158.48	-7585.23	-7584.73	-9943.58	-9946.15
bn2_bn2	2	-8692.36	-8455.83	-11998.23	-11997.40	-15762.19	-15765.33
bn3_bn3	3	-12062.23	-11753.55	-16409.28	-16408.11	-21581.80	-21585.31
bn4_bn4	4	-15431.48	-15051.34	-20820.52	-20819.03	-27402.28	-27406.03
bn5_bn5	5	-18801.41	-18348.99	-25231.70	-25229.88		-33226.59
bn6_bn6	6	-22169.85	-21646.22	-29642.74	-29640.59		-39047.26

Table S231: Strong Correlation Diagnostic (in %) derived from TAEs of the BNx_BNx dimers calculated using a cc-pVTZ basis set.

TZ	BNx_BNx				
System	Rings	(TPSS@HF-HF)	(TPSS@HF-HF)	(TPSS@TPSS-TPSS@HF)	(TPSS@TPSS-TPSS@HF)
		CCSD(T)	LNO-CCSD(T) Tight	CCSD(T)	LNO-CCSD(T) Tight
bn1_bn1	1	-24.41	-24.40	1.65	1.65
bn2_bn2	2	-22.47	-22.47	1.50	1.50
bn3_bn3	3	-21.57	-21.57	1.43	1.43
bn4_bn4	4	-21.05	-21.05	1.39	1.39
bn5_bn5	5		-20.71		1.36
bn6_bn6	6		-20.48		1.34

Table S232: Total Atomization energies (in kJ/mol) for the Coronene dimers using a cc-pVTZ basis set.

TZ			TAEs				
System	Rings	X_TPSS@TPSS	X_TPSS@HF	HF	HF	CCSD(T)	LNO-CCSD(T)
				ORCA	MRCC		Tight
cc_cc_pd	7	-21600.25	-19480.43	-29477.01	-29474.48		-39084.80
cc_cc	7	-21923.04	-19812.17	-29484.65	-29482.12		-39051.13
ccc_ccc	19	-48242.85	-43608.21	-62807.41	-62801.73		-83738.88

Table S233: Total Atomization energies (in kJ/mol) for the Cx_Cx sandwich-structured dimers using a cc-pVTZ basis set.

TZ	Cx_Cx		TAEs				
System	Rings	X_TPSS@TPSS	X_TPSS@HF	HF	HF	CCSD(T)	LNO-CCSD(T)
				ORCA	MRCC		Tight
c1_c1	1	-5856.80	-5284.65	-8555.53	-8554.97	-11149.11	-11154.98
c2_c2	2	-9488.99	-8569.53	-13433.21	-13432.20	-17620.39	-17630.47
c3_c3	3	-13106.36	-11838.51	-18269.02	-18267.58	-24066.35	-24075.53
c4_c4	4	-16717.44	-15100.14	-23084.34	-23082.48	-30495.88	-30506.54
c5_c5	5	-20326.20	-18358.11	-27887.76	-27885.48		-36931.49
c6_c6	6	-23933.86	-21613.72	-32683.92	-32681.22		-43353.19

Table S234: Strong Correlation Diagnostic (in %) derived from TAEs of the Cx_Cx sandwich-structured dimers calculated using a cc-pVTZ basis set.

TZ	Cx_Cx				
System	Rings	(TPSS@HF-HF)	(TPSS@HF-HF)	(TPSS@TPSS-TPSS@HF)	(TPSS@TPSS-TPSS@HF)
		CCSD(T)	LNO-CCSD(T) Tight	CCSD(T)	LNO-CCSD(T) Tight
c1_c1	1	-29.34	-29.32	5.13	5.13
c2_c2	2	-27.60	-27.59	5.22	5.22
c3_c3	3	-26.72	-26.71	5.27	5.27
c4_c4	4	-26.18	-26.17	5.30	5.30
c5_c5	5		-25.80		5.33
c6_c6	6		-25.53		5.35

Table S235: Total Atomization energies (in kJ/mol) for the Alkapolyene relaxed dimers using a cc-pVTZ basis set.

TZ	Cx_Cx		TAEs				
System	D-Bonds	X_TPSS@TPSS	X_TPSS@HF	HF	HF	CCSD(T)	LNO-CCSD(T)
				ORCA	MRCC		Tight
c2_c2	1	-2164.49	-1955.47	-3578.32	-3578.17	-4609.47	-4611.11
c4_c4	2	-4069.25	-3680.69	-6363.62	-6363.28	-8265.18	-8268.28
c6_c6	3	-5983.00	-5413.82	-9151.72	-9151.18	-11928.47	-11933.24
c8_c8	4	-7898.37	-7148.78	-11940.16	-11939.42	-15597.18	-15600.00
c10_c10	5	-9813.71	-8883.36	-14729.41	-14728.48	-19264.56	-19267.28
c12_c12	6	-11729.74	-10618.98	-17519.25	-17518.13	-22932.30	-22934.85
c14_c14	7	-13645.08	-12354.20	-20307.34	-20306.02	-26600.31	-26602.54

Table S236: Strong Correlation Diagnostic (in %) derived from TAEs of the Alkapolyene relaxed dimers calculated using a cc-pVTZ basis set.

TZ	Cx_Cx				
System	D-Bonds	(TPSS@HF-HF)	(TPSS@HF-HF)	(TPSS@TPSS-TPSS@HF)	(TPSS@TPSS-TPSS@HF)
		CCSD(T)	LNO-CCSD(T) Tight	CCSD(T)	LNO-CCSD(T) Tight
c2_c2	1	-35.21	-35.19	4.53	4.53
c4_c4	2	-32.46	-32.45	4.70	4.70
c6_c6	3	-31.34	-31.32	4.77	4.77
c8_c8	4	-30.72	-30.71	4.81	4.81
c10_c10	5	-30.35	-30.34	4.83	4.83
c12_c12	6	-30.09	-30.09	4.84	4.84
c14_c14	7	-29.90	-29.90	4.85	4.85

Table S237: Total Atomization energies (in kJ/mol) for the Alkapolyene fixed dimers using a cc-pVTZ basis set.

TZ	Cx_Cx		TAEs				
System	D-Bonds	X_TPSS@TPSS	X_TPSS@HF	HF	HF	CCSD(T)	LNO-CCSD(T)
				ORCA	MRCC		Tight
2_c2	1	-2164.49	-1955.47	-3578.32	-3578.17	-4609.47	-4611.11
c4_c4	2	-4065.87	-3675.25	-6367.29	-6366.94	-8264.53	-8267.66
c6_c6	3	-5978.85	-5405.66	-9158.88	-9158.34	-11926.66	-11931.38
c8_c8	4	-7889.17	-7133.08	-11951.27	-11950.54	-15594.07	-15596.71
c10_c10	5	-9803.19	-8864.34	-14743.98	-14743.05	-19260.06	-19262.53
c12_c12	6	-11716.08	-10594.91	-17536.74	-17535.61	-22926.40	-22928.68
c14_c14	7	-13627.88	-12324.08	-20329.50	-20328.18	-26592.94	-26594.86

Table S238: Strong Correlation Diagnostic (in %) derived from TAEs of the Alkapolyene fixed dimers calculated using a cc-pVTZ basis set.

TZ	Cx_Cx				
System	D-Bonds	(TPSS@HF-HF)	(TPSS@HF-HF)	(TPSS@TPSS-TPSS@HF)	(TPSS@TPSS-TPSS@HF)
		CCSD(T)	LNO-CCSD(T) Tight	CCSD(T)	LNO-CCSD(T) Tight
c2_c2	1	-35.21	-35.19	4.53	4.53
c4_c4	2	-32.57	-32.56	4.73	4.72
c6_c6	3	-31.47	-31.46	4.81	4.80
c8_c8	4	-30.90	-30.89	4.85	4.85
c10_c10	5	-30.53	-30.52	4.87	4.87
c12_c12	6	-30.28	-30.28	4.89	4.89
c14_c14	7	-30.10	-30.10	4.90	4.90

We calculated $TAE_X[(T)]$ (Cluster 3 in Table 1 in Ref. S2) to confirm the observation above.

Table S239: $TAE_X[(T)]$ Correlation Diagnostic for different series calculated using a cc-pVTZ basis set.

Subunits	Cx_Cx_PD	Cx_Cx	Polyenes relax.	Polyenes fix.	BNx_BNx	BNx_NBx
1	1.93	1.92	1.25	1.25	1.64	1.62
2	2.16	2.14	1.55	1.54	1.78	1.76
3	2.28	2.26	1.68	1.67	1.85	1.82
4	2.36	2.35	1.76	1.74	1.89	1.86
5	1.73*	1.71*	1.81	1.79	1.18*	1.15*
6	1.77*	1.76*	1.84	1.82	1.20*	1.16*
7			1.87	1.85		

$*$ cc-pVDZ results

Additionally, from MOLPRO output, we gathered T_1 and D_1 diagnostic from calculations at the PNO-LCCSD(T) level.

Table S240: T1 Diagnostic for different series calculated using a cc-pVTZ basis set.

Subunits	Cx_Cx_PD	Cx_Cx	Polyenes relax.	BNx_BNx	BNx_NBx
1	0.00975	0.00961*	0.01045	0.01405	0.01415
2	0.01009	0.00999*	0.01093	0.01397	0.01409
3	0.01046	0.01037*	0.01111	0.01399	0.01397
4	0.01082	0.01067*	0.01122	0.01403	0.01397
5	0.01107*	0.01091*	0.01129	0.01521*	0.01524*
6	0.01133*	0.01111*	0.01135	0.01522*	0.01523*
7			0.01138		

∗ cc-pVDZ results

Table S241: D1 Diagnostic for different series calculated using a cc-pVTZ basis set.

Subunits	Cx_Cx_PD	Cx_Cx	Polyenes relax.	BNx_BNx	BNx_NBx
1	0.02593	0.02685*	0.03176	0.04543	0.04502
2	0.02756	0.02767*	0.03437	0.04612	0.04603
3	0.03064	0.03062*	0.03440	0.04646	0.04552
4	0.03407	0.03384*	0.03694	0.04672	0.04616
5	0.03720*	0.03629*	0.03770	0.05170*	0.05131*
6	0.03884*	0.03820*	0.03782	0.05189*	0.05107*
7			0.03803		

∗ cc-pVDZ results

HOMO-LUMO gaps for all systems

Table S242: HOMO-LUMO gaps for Polyacenes, in eV

Num. of Rings	Cx_Cx_pd dimers	Cx_Cx SANDWICH dimers	polyacenes monomers
1	12.15	11.97	12.70
2	10.07	9.57	10.32
3	8.44	7.90	8.64
4	7.30	6.74	7.49
5	6.48	5.89	6.64
6	5.86	5.25	6.00

Table S243: HOMO-LUMO gaps for Borazine-like Polyacenes, in eV

Num. of Rings	BNx_NBx dimers	BNx_BNx dimers	Borazine-acenes monomers
1	14.40	14.38	15.11
2	13.50	13.78	14.27
3	13.01	13.47	13.83
4	12.78	13.33	13.61
5	12.70	13.28	13.50
6	12.63	13.26	13.43

Table S244: HOMO-LUMO gaps for Alkapolyenes, in eV

Num. of Double Bonds	Polyenes relaxed dimers	Polyenes fixed dimers	polyenes monomers
1	13.96	13.96	14.51
2	11.37	11.59	11.98
3	9.92	10.19	10.53
4	9.00	9.31	9.62
5	8.39	8.72	9.01
6	7.97	8.29	8.58
7	7.63	7.99	8.26

Table S245: HOMO-LUMO gaps for Coronenes, in eV

	dimer	monomer
Coronene PD	8.22	8.76
Cororнene sand	7.98	8.76
Circumcoronene sand	5.99	6.77

Revised Figure 2c for Previous Paper

During the preparation of the present paper, we noticed incorrectness in Figure 2c of our previously published article, specifically for the **LNO-CCSD(T)-vTight** line. The error was limited to the Figure 2c and do not affect the conclusions reported in previous study. A corrected version of Figure 2c is provided below. This updated Figure supersedes the originally published version and should be used for reference in conjunction with both studies.

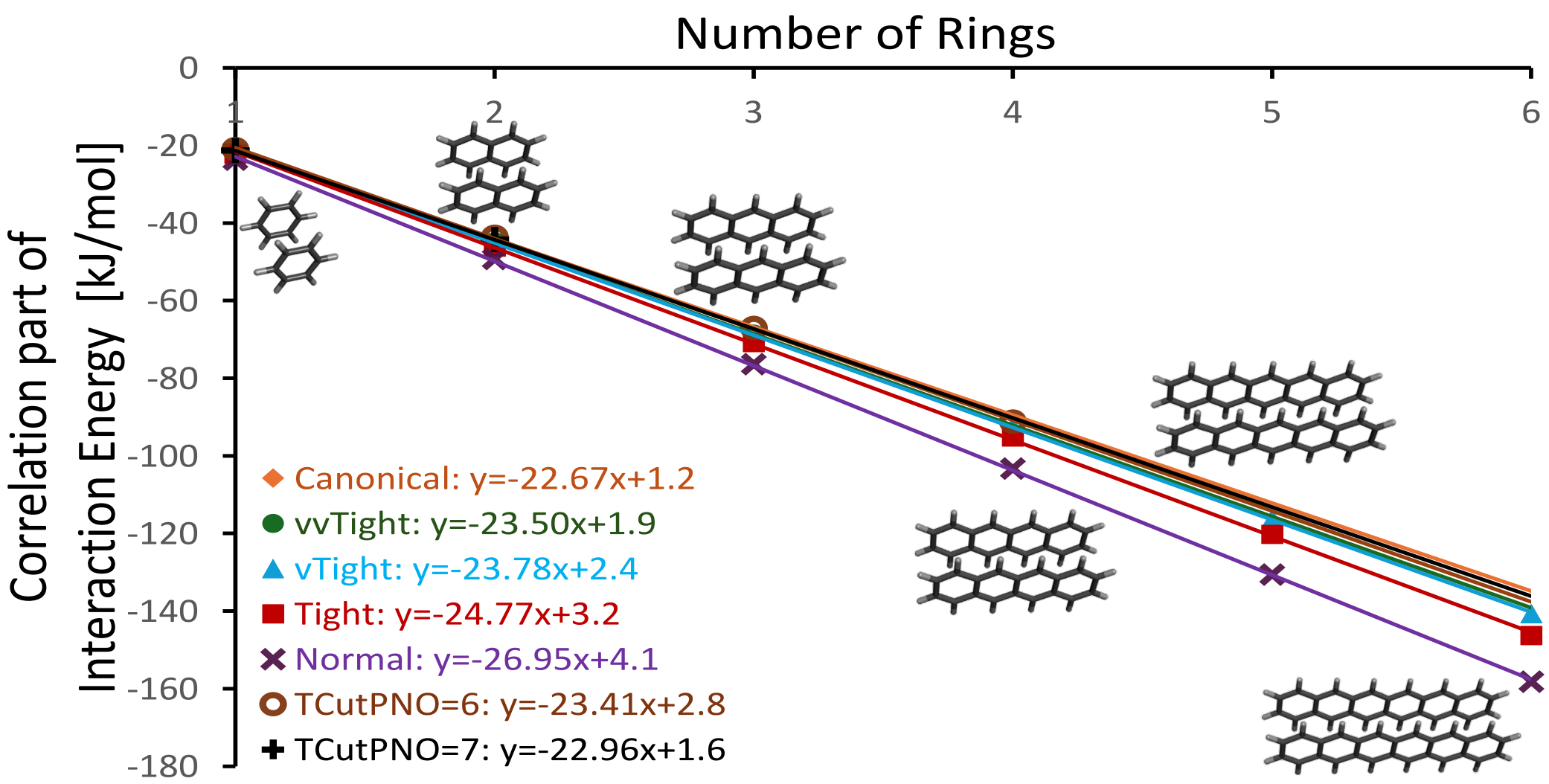

Figure S6: Correlation energies (kJ/mol) derived from various coupled cluster methods in as a function of system size (per number of rings) for the sandwich-structured acene series in cp-corrected aug-cc-pVTZ basis set.

Examples of input files

Table S246: Input example for the benzene PD dimer MOLPRO 2024

```
gthresh,energy=1d-9
geomtyp=xyz
geom={
include BenzeneDimerPD.xyz          ! dimer structure
}
basis=cc-pvtz                       !basis
df-hf                               !density-fitted Hartree-Fock
{pno-lccsd(t), DOMOPT=TIGHT}        !correlation calculation
```

Table S247: Input example for the benzene PD dimer ORCA 5.0.4

```
! DLPNO-CCSD(T1) cc-pVTZ def2/J cc-pVTZ/JK cc-pVTZ/C   RIJCOSX VeryTightSCF TightPNO NoPop defGRID3
%pal nprocs 12 end
%maxcore 12000
* xyz 0 1
GEOMETRY HERE
*
%mdci TCutPNO 1e-6 end
```

Table S248: Input example for the benzene PD dimer MRCC

```
calc=LNO-CCSD(T)
basis=cc-pVQZ
mem=330000MB
localcc=2024
lcorthr=vTight
localcorrsymm=on
ccprog=ccsd
charge=0
mult=1
geom=xyz
12

GEOMETRY HERE
```

References

(S1) Fishman, V.; Lesiuk, M.; Martin, J. M.; Daniel Boese, A. Another angle on benchmarking noncovalent interactions. *Journal of Chemical Theory and Computation* **2025**, *21*, 2311–2324.

(S2) Martin, J. M. L.; Santra, G.; Semidalas, E. An exchange-based diagnostic for static correlation. *AIP Conference Proceedings* **2022**, *2611*, 020014.
